# Genomic variations in the 3′‐termini of *Rice stripe virus* in the rotation between vector insect and host plant

**DOI:** 10.1111/nph.15246

**Published:** 2018-06-08

**Authors:** Wan Zhao, Zhongtian Xu, Xiaoming Zhang, Meiling Yang, Le Kang, Renyi Liu, Feng Cui

**Affiliations:** ^1^ State Key Laboratory of Integrated Management of Pest Insects and Rodents Institute of Zoology Chinese Academy of Sciences Beijing 100101 China; ^2^ Shanghai Center for Plant Stress Biology Chinese Academy of Sciences Shanghai 201602 China; ^3^ University of Chinese Academy of Sciences Beijing 100049 China; ^4^ Center for Agroforestry Mega Data Science and FAFU‐UCR Joint Center for Horticultural Biology and Metabolomics Haixia Institute of Science and Technology Fujian Agriculture and Forestry University Fuzhou 350002 China

**Keywords:** genomic variation, host plant, *Rice stripe virus*, RNA virus, vector insect

## Abstract

- A large number of plant RNA viruses circulate between plants and insects. For RNA viruses, host alternations may impose a differential selective pressure on viral populations and induce variations in viral genomes. Here, we report the variations in the 3′‐terminal regions of the multiple‐segment RNA virus *Rice stripe virus* (RSV) that were discovered through *de novo* assembly of the genome using RNA sequencing data from infected host plants and vector insects.
- The newly assembled RSV genome contained 16‐ and 15‐nt extensions at the 3′‐termini of two genome segments compared with the published reference RSV genome. Our study demonstrated that these extensional sequences were consistently observed in two RSV isolates belonging to distinct genetic subtypes in RSV‐infected rice, wheat and tobacco. Moreover, the *de novo* assembled genome of *Southern rice black‐streaked dwarf virus* also contained 3′‐terminal extensions in five RNA segments compared with the reference genome.
- Time course experiments confirmed that the 3′‐terminal extensions of RSV were enriched in the vector insects, were gradually eliminated in the host plant and potentially affected viral replication.
- These findings indicate that variations in the 3′‐termini of viral genomes may be different adaptive strategies for plant RNA viruses in insects and plants.

## Introduction

Most plant viruses depend on specific plant‐feeding insects as the primary vector for transmission (Bragard *et al*., 2013). Persistent‐propagative plant viruses have a more complicated interaction with their vector insects than non‐persistent viruses because of their characteristics of replication and systemic invasion of the vector insect tissues before transmission via the salivary glands (Ammar el *et al*., 2009; Bragard *et al*., 2013). Vector insects are usually indispensable for the pathogenicity of persistent‐propagative plant viruses, which suggests that alterations in the viral genome may be induced when the viruses are incubated in the vector insects. Therefore, comparisons of the viral genomes in vector insects and host plants may provide important clues to understand the adaptation strategies of a plant virus in the two hosts.

As a typical persistent‐propagative plant virus, *Rice stripe virus* (RSV) causes rice stripe disease, which is one of the most serious rice diseases in Eastern Asian countries (Falk & Tsai, 1998; Xiong *et al*., 2008). In addition to rice, RSV also infects wheat, maize, oat, weeds and even tobacco (*Nicotiana benthamiana*) under experimental conditions (Falk & Tsai, 1998; Xiong *et al*., 2008; Sun *et al*., 2011). RSV is a single‐stranded RNA virus and belongs to the *Tenuivirus* genus. The published reference genome of RSV is 17 144 nucleotides (nt) and contains four RNA segments: RNA1 (8969 nt), RNA2 (3514 nt), RNA3 (2504 nt) and RNA4 (2157 nt) (Takahashi *et al*., 1993; Toriyama *et al*., 1994). RNA1 is negative‐sense and encodes RNA‐dependent RNA polymerase (RdRp). The other three segments are ambisense and encode NS2, NSvc2 (a putative membrane glycoprotein), NS3 (a gene silencing suppressor), CP (a nucleocapsid protein), SP (a disease‐specific protein) and NSvc4 (a movement protein) (Cho *et al*., 2013). The 5′‐ and 3′‐terminal sequences of the four RNA segments were first determined through two‐dimensional mobility shift analysis of the first 18 bases (Takahashi *et al*., 1990). The 18 or 20 nucleotides at the 3′‐ and 5′‐ends of the four RNA segments are conserved, and each segment may form a panhandle structure because of the complementary terminal sequences, and this structure is a potential recognition landmark for RNA polymerase (Takahashi *et al*., 1990). Based on the nucleotide sequence of the *CP* gene, RSV isolates from China can be divided into two subtypes: the populations from Eastern China (represented by Jiangsu Province) belong to subtype I, and the populations from Southwest China (represented by Yunnan Province) belong to subtype II (Wei *et al*., 2009).

RSV is efficiently transmitted between plants in the field by the small brown planthopper *Laodelphax striatellus* (Falk & Tsai, 1998). Although RSV can replicate in both vector insects and host plants, different symptoms are induced in the two types of host. Instead of producing severe diseases as it does in host plants, RSV seems to live in harmony with planthoppers without causing intense immune responses or inducing obvious damage (Zhang *et al*., 2010; Zhao *et al*., 2016a). Moreover, the RSVs from planthoppers and from rice plants exhibit different pathogenicities. The insect‐derived RSV destroys the chloroplast structure and induces a typical yellow stripe symptom, whereas the plant‐derived RSV only causes chlorosis in rice leaves (Zhao *et al*., 2016b). Thus, it is highly possible that genomic and/or population diversifications may occur during the spread of RSV between vector insects and host plants because of the distinct living conditions and selective pressures.

To investigate the genomic variations in the RSV populations that originate from vector insects and host plants, we sequenced the viral genomes in viruliferous planthoppers and RSV‐infected rice, and performed *de novo* genome assembly. Sixteen‐ and 15‐nt extension sequence variations at the 3′‐termini of the RNA1 and RNA2 segments were observed in different RSV isolates and RSV‐infected host plants. The variations in the ratios of the RNA segments with the 3′‐extension sequences were explored during the viral rotation between the vector insects and host plants.

## Materials and Methods

### Virus isolates and culture of insects and plants

The viruliferous and non‐viruliferous small brown planthopper strains used in this study were established from a field population collected in Hai'an, Jiangsu Province, China. The viruliferous strain contained the Jiangsu isolate (JSHA) of RSV. The planthoppers were reared separately on 2–3‐cm seedlings of rice (*Oryza sativa* Huangjinqing) in glass incubators in the laboratory, as described previously (Zhao *et al*., 2016b). To maintain the RSV‐carrying frequency of the viruliferous strain at no less than 90%, non‐viruliferous individuals were discriminated and eliminated via dot enzyme‐linked immunosorbent assay (dot‐ELISA) using the monoclonal anti‐CP antibody every 3 months (Zhao *et al*., 2016b). Rice, wheat (*Triticum aestivum* jingdong 22) and tobacco (*Nicotiana benthamiana*) were grown in an insect‐free incubator at 25°C with 16 h of light daily. Another four RSV isolates were collected from RSV‐infected small brown planthoppers and rice plants in Kunming, Chuxiong and Yuxi of Yunnan Province in August 2017, and were stored at −80°C.

### RNA extraction, RNA‐Seq library construction and sequencing

Total RNAs were isolated from 20 fifth‐instar viruliferous planthopper nymphs or 20 fourth‐instar planthopper nymphs that had acquired RSV 5 d previously, and from 100 mg of RSV‐infected rice leaves (with the typical stripe symptoms) using TRIzol reagent (Invitrogen, Carlsbad, CA, USA) in accordance with the manufacturer's instructions. The concentrations and qualities of the RNA samples were measured using a NanoDrop spectrophotometer (Thermo Scientific, Waltham, MA, USA) and through gel electrophoresis. Four replicates each were prepared for the viruliferous planthoppers and RSV‐infected rice samples. One replicate was prepared for the planthoppers that had acquired RSV 5 d before sampling. Sequencing was performed in two batches. The first batch included the planthoppers that had acquired RSV 5 d previously, one viruliferous planthopper sample and one RSV‐infected rice sample. The second batch included the remaining three replicates of the viruliferous planthoppers and RSV‐infected rice leaves.

Five‐microgram samples of total RNA from the planthoppers and rice leaves were treated with a Ribo‐Zero rRNA Removal Kit (human/mouse/rat) or a Ribo‐Zero rRNA Removal Kit (plant leaf) (Epicentre, Madison, WI, USA), respectively, to deplete the rRNA. The retrieved RNA was fragmented by adding First‐Strand Master Mix (Invitrogen). First‐strand cDNA was generated with random hexamers with First‐Strand Master Mix and SuperScript II reverse transcription (Invitrogen). Second‐strand cDNA was generated using Second‐Strand Master Mix (Invitrogen) and dNTPs. The ends of the purified cDNA were repaired using End Repair Mix (Invitrogen). After adding ‘A’ bases or adapters to the 3′‐ends or both ends, the cDNA fragments were purified and then enriched by PCR. cDNA pair‐end libraries were then constructed following standard Illumina protocols and were sequenced with the Illumina HiSeq™ 4000 platform (Illumina, San Diego, CA, USA). Approximately 12 gigabases (Gb) of 101‐bp paired‐end raw data were generated for each library. The sequencing data were deposited in the Short Read Archive of the National Center for Biotechnology Information (NCBI) with the accession number SRP108307.

### *De novo* assembly of the RSV and *Southern rice black‐streaked dwarf virus* (SRBSDV) genomes

The qualities of the raw reads were evaluated with FastQC (http://www.bioinformatics.babraham.ac.uk/projects/fastqc). To obtain clean reads, low‐quality regions and adapter sequences were removed using SolexQA (v.2.2) and Cutadapt (v.1.3) (Martin, 2011), respectively. Reads shorter than 25 bases were discarded.

Clean reads from the first batches of the planthopper and rice libraries were *de novo* assembled to generate the transcriptome using Trinity 2.2.0 (−min_kmer_cov 2 −kmer 25 −min_contig_length 200). To obtain the RSV genome sequences, the assembled transcripts were compared with the reference RSV genome sequences from GenBank (NC_003755.1 for RNA1, NC_003754.1 for RNA2, NC_003776.1 for RNA3 and NC_003753.1 for RNA 4) using Blastn with an E‐value cutoff of 1e^−20^. Another assembly strategy was utilized to verify the assembly results. Clean reads were first mapped to the reference RSV genome sequences using Bwa (Li & Durbin, 2009) to retrieve the virus reads, which were then assembled using Trinity 2.2.0. Both strategies produced similar assembly results. The nucleotide sequences of the newly assembled RSV genome were deposited in NCBI with the accession numbers MF287953–MF287956. Each segment of the newly assembled RSV genome was aligned with that of the reference RSV genome from GenBank using ClustalW (Larkin *et al*., 2007).

The SRBSDV genome sequencing data were retrieved from the published work of Wang *et al*. (2016a). Two versions of the genome were assembled with SRBSDV reads from the viruliferous strains with high viral titres (HVT) and medium viral titres (MVT), and the resulting genome assemblies were compared with the published reference genome from GenBank (from NC_014708.1 to NC_014717.1) using the same methods as those used in the RSV genome assembly and comparison.

### Touchdown reverse transcription‐polymerase chain reaction (RT‐PCR) for determination of the 3′‐terminal sequences

Two micrograms of total RNA from the planthoppers or plant leaves were prepared for first‐strand cDNA synthesis using a Superscript III First‐Strand Synthesis System (Invitrogen) with random primers for the time course experiment or specific primers that were designed according to the newly assembled RSV genomic sequences RNA1‐3′R, RNA2‐3′R, RNA3‐3′R and RNA4‐3′R. The 3′‐terminal sequences of the four RNA segments were then amplified with touchdown RT‐PCR with the primer pairs RNA1‐3′F and RNA1‐3′R, RNA2‐3′F and RNA2‐3′R, RNA3‐3′F and RNA3‐3′R, and RNA4‐3′F and RNA4‐3′R. The primer sequences are listed in Supporting Information Table S1. The touchdown PCR protocol was as follows: 94°C for 3 min; 10 cycles of 94°C for 30 s, 68°C for 30 s (−1°C each cycle) and 72°C for 1 min; 25 cycles of 94°C for 30 s, 58°C for 30 s and 72°C for 1 min; and, finally, 72°C for 5 min.

### RT‐PCR

Two micrograms of total RNA from RSV‐infected planthoppers or plant leaves were prepared for first‐strand cDNA synthesis using a Superscript III First‐Strand Synthesis System (Invitrogen) with random primers. The fragments from the inner regions of RNA1 and RNA2 were amplified to measure the total RNA levels of RNA1 and RNA2 using primers RNA1‐inner‐F and RNA1‐inner‐R and primers RNA2‐inner‐F and RNA2‐inner‐R, respectively. The transcript levels of the *actin* genes of the small brown planthopper (Jiao *et al*., 2017), rice (AK100267) (Chern *et al*., 2013), wheat (TC234027) (Tenea *et al*., 2011) and tobacco (AY594294) (Liu *et al*., 2012) were quantified with the primers actin‐insect‐F and actin‐insect‐R, actin‐rice‐F and actin‐rice‐R, actin‐wheat‐F and actin‐wheat‐R, and actin‐tobacco‐F and actin‐tobacco‐R, respectively, and were used as internal controls to normalize the levels of the total input RNAs. The primer sequences are listed in Table S1. The PCR protocol was as follows: 94°C for 3 min; 30 cycles of 94°C for 30 s, 55°C for 30 s and 72°C for 30 s; and, finally, 72°C for 5 min.

### 5′‐Rapid amplification of cDNA ends (5′‐RACE) assays

The RNAs extracted from the viruliferous planthoppers and RSV‐infected rice leaves were employed to analyse the 5′‐ends of the viral RNA segments using the SMARTer^®^ RACE 5′/3′ Kit (Takara, Mountain View, CA, USA). The first‐strand cDNA templates from 5′‐RACE were synthesized following the instructions provided in the user manual. Two micrograms of RNA were combined with 2 μM of Random Primer Mix and incubated at 72°C for 3 min. After cooling to 4°C over 2 min, SMARTer II A Oligonucleotide, Master Mix, RNase Inhibitor and SMARTScribe Reverse Transcriptase were added to reach a final volume of 20 μl. The reaction was incubated at 42°C for 90 min and then heated at 70°C for 10 min.

Two rounds of nested PCR were conducted using an Applied Biosystems^®^ ProFlex™ Thermal Cycler (Thermo Fisher Scientific, Bartlesville, OK, USA). The first round of amplification included the 5′‐first‐strand cDNA, PCR Master Mix, 10 × UPM (from the kit) and 10 pmol of 5′‐gene‐specific primer (5′‐GPS) for the 5′‐end of each RNA (i.e. RNA1‐GSP, RNA2‐GSP, RNA3‐GSP or RNA4‐GSP, Table S1). The second round of amplification included 1 μl of a 1 : 50 dilution of the product from the first round of PCR, PCR Master Mix, 10 × UPS (from the kit) and 10 pmol of nested 5′‐GPS (i.e. RNA1‐nested GSP, RNA2‐nested GSP, RNA3‐nested GSP or RNA4‐nested GSP, Table S1). The amplification reaction was conducted under the following conditions: 94°C for 3 min; 10 cycles of 94°C for 30 s and 68°C for 30 s (−1°C each cycle); 72°C for 2 min; 25 cycles of 94°C for 30 s, 58°C for 30 s and 72°C for 2 min; and, finally, 72°C for 5 min. The PCR products were purified and inserted into a pEASY‐Blunt Zero Cloning vector (CB501‐01, Transgen, Beijing, China) for sequencing.

### Time course analyses of the viral 3′‐terminal extensions containing RNA1 and RNA2 in the planthoppers and plants

Non‐viruliferous fourth‐instar nymphs were fed on an artificial diet containing the JSHA isolate of RSV crude preparations from the infected rice leaves, as described previously (Zhao *et al*., 2016a). After feeding on RSV for 8 h, the nymphs were transferred to healthy rice seedlings and then collected after 2, 5, 8, 10, 12, 14 and 22 d. Four replicates and five insects per replicate at each time point were prepared.

Five viruliferous fifth‐instar nymphs were fed on one healthy rice seedling or wheat seedling for 4 h and then removed. The rice seedlings were collected after 1, 2, 3, 4, 5, 10 or 15 d of culture at 25°C. The wheat seedlings were collected after 1, 3, 5, 10, 15 or 30 d of culture at 25°C. Three replicates and three leaves per replicate were prepared.

The JSHA isolates of the RSV crude preparations were extracted from 100 viruliferous planthoppers in 40 ml of 0.01 M PBS buffer (pH 7.2), as described previously (Zhao *et al*., 2016b). After the quantification of RSV in the crude preparations using ELISA at the CP level, moderate amounts of RSV were mechanically inoculated into the leaves of tobacco seedlings at the six‐ to seven‐leaf stage, as described previously (Wei *et al*., 2009). The inoculated local leaves were collected after 1, 3, 5, 10, 15 or 30 d of culture at 25°C. Three replicates and two leaves per replicate were prepared.

RNA1 and RNA2 with the extended 3′‐terminal sequences were identified by amplification of the 3′‐non‐translated regions containing the extended terminal sequences using touchdown RT‐PCR. The fragments from the inner regions of RNA1 and RNA2 were amplified to measure the total RNA levels of RNA1 and RNA2, respectively, and were analysed in 1% DNA gel after a five‐fold dilution. The ratios of RNA1 or RNA2 to the extended 3′‐terminal sequences were determined by associating the relative grey values of the extended 3′‐terminal sequences to those of the RNA levels of RNA1 and RNA2 using ImageJ (National Institutes of Health, Bethesda, MD, USA). The differences were statistically evaluated with one‐way ANOVA for multiple comparisons with Spss 17.0 software. The values are represented as the means ± SEs.

### Quantification of the replication levels of RNA1 and RNA2 from insect‐derived and plant‐derived RSV in rice plants

Comparable amounts of the JSHA isolates of RSV from the viruliferous planthoppers and the infected rice leaves with obvious stripe symptoms were microinjected into the leaves of healthy 2‐wk‐old rice. The inoculated rice seedlings were cultured at 25°C and collected every day until 5 d. Eight replicates and three leaves in each replicate were prepared.

The replications of RSV RNA1 and RNA2 were determined by the quantification of the RNA levels of the 5′‐non‐translated region of RNA1 with the primers RNA1‐5′NTR‐F and RNA1‐5′NTR‐R or by comparison of the intergenic region of RNA2 with the primers RNA2‐IR‐F and RNA2‐IR‐R using quantitative real‐time PCR on a Light Cycler 480 II (Roche, Basel, Switzerland). The transcript level of the rice *ubiquitin* (AK061988) was quantified with the primers ubq‐F and ubq‐R as an internal control. The primer sequences are listed in Table S1. The thermal cycling conditions were as follows: 95°C for 10 min; 45 cycles of 95°C for 10 s, 58°C for 20 s and 72°C for 20 s; and, finally, 65°C for 1 min. The RNA level of each RSV segment relative to that of *ubiquitin* is reported as the mean ± SE. A Student's *t*‐test was performed to evaluate the difference between the two means using Spss 17.0. The RNA1 and RNA2 with the extended 3′‐terminal sequences were determined using touchdown RT‐PCR as described earlier in the Materials and Methods section.

### Purification of the RSV ribonucleoprotein particles

RSV crude preparations were extracted from 10 g of rice leaves infected with JSHA or KMXD isolates of RSV in 40 ml of 0.01 M PBS buffer (pH 7.2) containing a protease inhibitor cocktail (ThermoFisher Scientific, Waltham, MA, USA). After centrifugation at 1344 ***g*** for 5 min and 8400 ***g*** for 15 min at 4°C, the supernatant containing RSV was collected and incubated with RNase A Solution (Promega) at room temperature for 30 min to remove the RNAs from host cells and viral mRNAs. To precipitate the RSV particles, the mixtures were adjusted to 8% polyethylene glycol 6000 (Lablead) and 0.1 M NaCl, and then stirred in ice for 1 h. After centrifugation at 12 000 ***g*** for 1 h at 4°C, the pellets were dissolved in 0.01 M PBS buffer and then purified with Dynabeads^®^ Protein G (Novex^®^ by Thermo Fisher Scientific) based on immunoaffinity chromatography, as described by Chand *et al*. (2016). Fifty microlitres of Protein G beads were prepared with 10 μg anti‐CP monoclonal antibody, which was bound and then incubated with 1000 μl of viral crude preparations at 4°C overnight. The antibody–CP–viral ribonucleoprotein complex was dissociated from the beads with elution buffer (Novex^®^, Thermo Fisher Scientific). The purified ribonucleoprotein particles were then diluted with 0.01 M PBS, mounted on grids and stained with 4% uranyl acetate (Ishikawa *et al*., 1989). The grids were examined using a JEM‐1400 transmission electron microscope (Jeol, Tokyo, Japan) for virion observation.

### Phylogenetic analysis of the RSV isolates

The *CP* gene was amplified with RT‐PCR performed on Jiangsu JSHA isolates and the four isolates from Yunnan using the primers CP‐RT‐F and CP‐RT‐R (Table S1), as described previously (Wei *et al*., 2009). The *CP* sequences obtained were deposited in NCBI with accession numbers MG680206 (isolate JHSA), MG680207 (isolate KMXD), MG680208 (isolate CXYL), MG680209 (isolate CXYH) and MG680210 (isolate YXLC).

The PCR products were inserted into pEASY‐Blunt Zero Cloning Vectors (TransGen, Beijing, China) and transformed into *Escherichia coli* T1 (TransGen) for sequencing. The *CP* sequences obtained from the five isolates and from another 75 RSV isolates reported by other laboratories (Kakutani *et al*., 1991; Zhu *et al*., 1991; Qu *et al*., 1997; Wei *et al*., 2009) were included in the phylogenetic analysis. The RNA3 of *Maize stripe virus* (S40180 in GenBank) was used as an outgroup. Multiple nucleotide sequence alignments were performed using ClustalW (Thompson *et al*., 1994). The phylogenetic tree was constructed using the neighbour‐joining method with the Kimura two‐parameter model implemented in Mega 5.0. Gaps were treated as a fifth character state. Evaluations of the statistical confidence in nodes were based on 1000 bootstrap replicates. Branches with < 50% bootstrap values were collapsed.

## Results

### *De novo* assembly of the RSV genome using RNA sequencing data from vector insects and host plants

**Figure 1:**
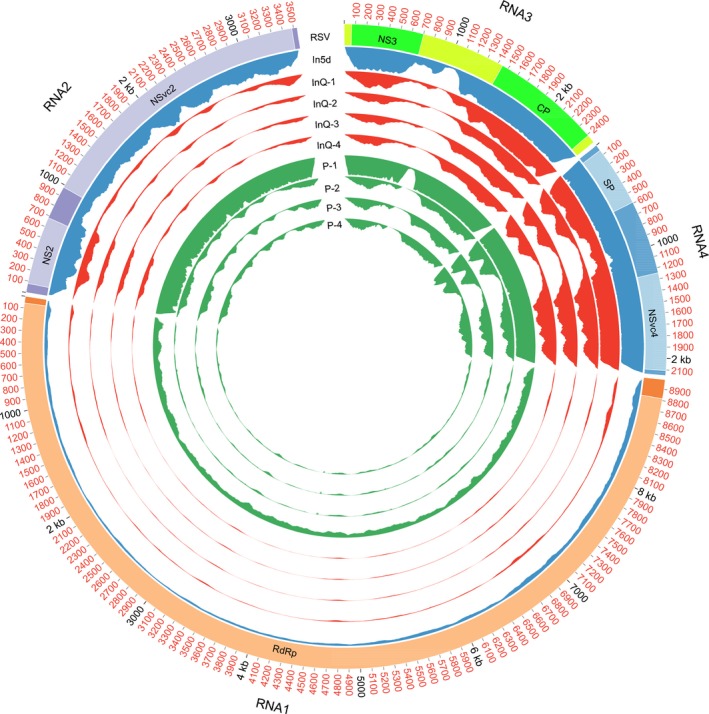
Circular representations of the *Rice stripe virus* (RSV) read coverages along the viral genome in different samples. The outer scale marks the genomic positions. The first track indicates the genome annotations. The heatmaps on tracks 2–10 represent the viral read coverages in different samples. In5d, the small brown planthoppers (*Laodelphax striatellus*) that had acquired RSV 5 d previously. InQ, the four replicates of the viruliferous small brown planthoppers. P, the four replicates of the RSV‐infected rice (*Oryza sativa* Huangjinqing) with obvious stripe symptoms.

To assemble the RSV genome, we performed deep sequencing of the transcriptomes of the small brown planthoppers that had acquired RSV Jiangsu isolate (JSHA) 5 d previously, the viruliferous planthoppers and RSV‐infected rice. Totals of 115 586 652 clean reads from the planthoppers that had acquired RSV 5 d previously, 742 362 522 from viruliferous planthoppers and 779 708 192 from rice were obtained (Table S2). Following mapping to the published RSV reference genome (Hamamatsu *et al*., 1993; Toriyama *et al*., 1994), 953 253 clean reads (0.82% of total clean reads) from the planthoppers that had acquired RSV 5 d previously, 1368 886 (0.18% of total clean reads) from viruliferous planthoppers and 1780 260 (0.23% of total clean reads) from rice were retrieved as RSV reads (Table S2). Higher ratios of RSV reads were captured from the first sequencing batch, i.e. the planthoppers that had acquired RSV 5 d previously (In5d, 0.82%), the first replicate of viruliferous planthoppers (InQ‐1, 0.49%) and rice samples (P‐1, 1.39%) than from the other replicates in the second sequencing batch (Fig. 1; Table S2). These differences were probably a result of the varied viral loads between the samples, or the different rRNA removal efficiencies in the two independent sequencing batches. Thus, the virus reads from the first sequencing batch were *de novo* assembled to generate the genomes of RSV from vector insects and host plants. Because the assembled genome from the host plants was longer than the other two assembled genomes from the vector insect samples, it was used as the new reference to map back RSV reads and for comparison with the published genome. After mapping back the RSV reads, the average coverage depths of the four RNA segments of RSV in the first sequencing batch ranged from 936 to 7552 times for the planthoppers that had acquired RSV 5 d previously, from 377 to 6714 times for the viruliferous planthoppers, and from 2192 to 7627 times for the infected rice (Table S3); all of these coverage depths were sufficient for effective genome assembly.

### The newly assembled RSV genome contains 16‐ and 15‐nt extensions at the 3′‐termini of two genome segments

The newly assembled genome of RSV was 17 155 nt, which was larger than the published reference genome (17 144 nt) (Takahashi *et al*., 1993; Toriyama *et al*., 1994). The lengths of the four RNA segments from RNA1 to RNA4 in the newly assembled genome were 8985, 3532, 2488 and 2150 nt, respectively. RNA1 and RNA2 were longer than the corresponding sequences in the reference genome, which were 8969 and 3514 nt, respectively. The extended nucleotide sequence at the 3′‐terminus of RNA1 was a 16‐nt (5′‐CATGAACATGCAAGGG‐3′) sequence that was sequenced four times. This sequence was identical to the 8874–8889‐nt fragment in the upstream region of RNA1. The extended nucleotide sequence at the 3′‐terminus of RNA2 was a 15‐nt (5′‐AGCATGTCTCAAAGT‐3′) sequence that was sequenced 11 times. Similarly, the 15‐nt extension sequence was identical to the 3241–3255‐nt fragment in the upstream region of RNA2. The assembled RNA3 and RNA4 were shorter than the corresponding published reference sequences, which were 2504 and 2157 nt, respectively (Zhu *et al*., 1991, 1992). The truncated nucleotide sequences occurred in intergenic regions and the 3′‐termini.

To verify the 3′‐termini of the four RNA segments, touchdown RT‐PCR was performed using terminal sequence‐specific primers. Fragments of 784, 521, 793 and 795 nt were amplified from the 3′‐termini of RNA1 to RNA4, respectively, and sequenced by Sanger sequencing. The existence of the 16‐ and 15‐nt extensions at the 3′‐termini of RNA1 and RNA2 was confirmed (Fig. S1). The 3′‐termini of RNA3 and RNA4 were found to be the same as those of the published reference genome and not shorter than the presented assembled genome (Fig. S1). The 5′‐terminal sequences of the four RNA segments were verified by 5′‐RACE experiments and proven to be the same as those of the published reference genome (Fig. S2).

**Figure 2:**
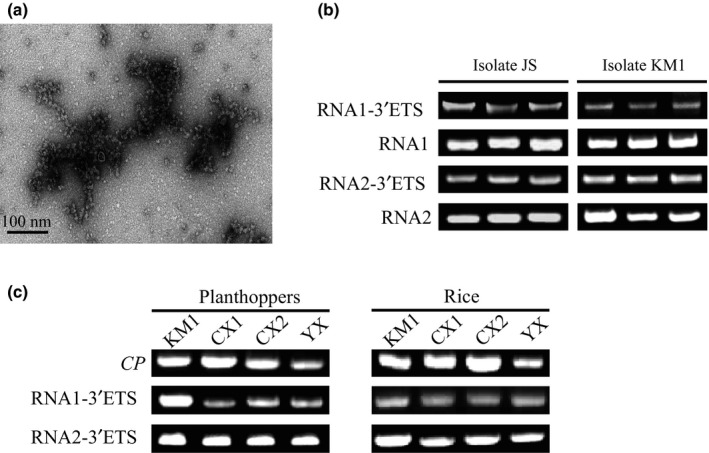
Determination of the 3′‐terminal extensions of *Rice stripe virus* (RSV) RNA1 and RNA2 from purified viral ribonucleoprotein particles and infected samples. (a) Purified RSV ribonucleoprotein particles of the JSHA isolate stained with 4% uranyl acetate and observed under a transmission electron microscope. (b) Amplification of the extended 3′‐terminal sequences of RNA1 and RNA2 from the purified ribonucleoprotein particles of JSHA and KMXD isolates through touchdown reverse transcription‐polymerase chain reaction (RT‐PCR). The total amounts of viral RNA1 and RNA2 (amplifying fragments from the inner regions) were amplified as internal controls. (c) Amplification of the extended 3′‐terminal sequences of RNA1 and RNA2 from four Yunnan isolates through touchdown RT‐PCR.

**Figure 3:**
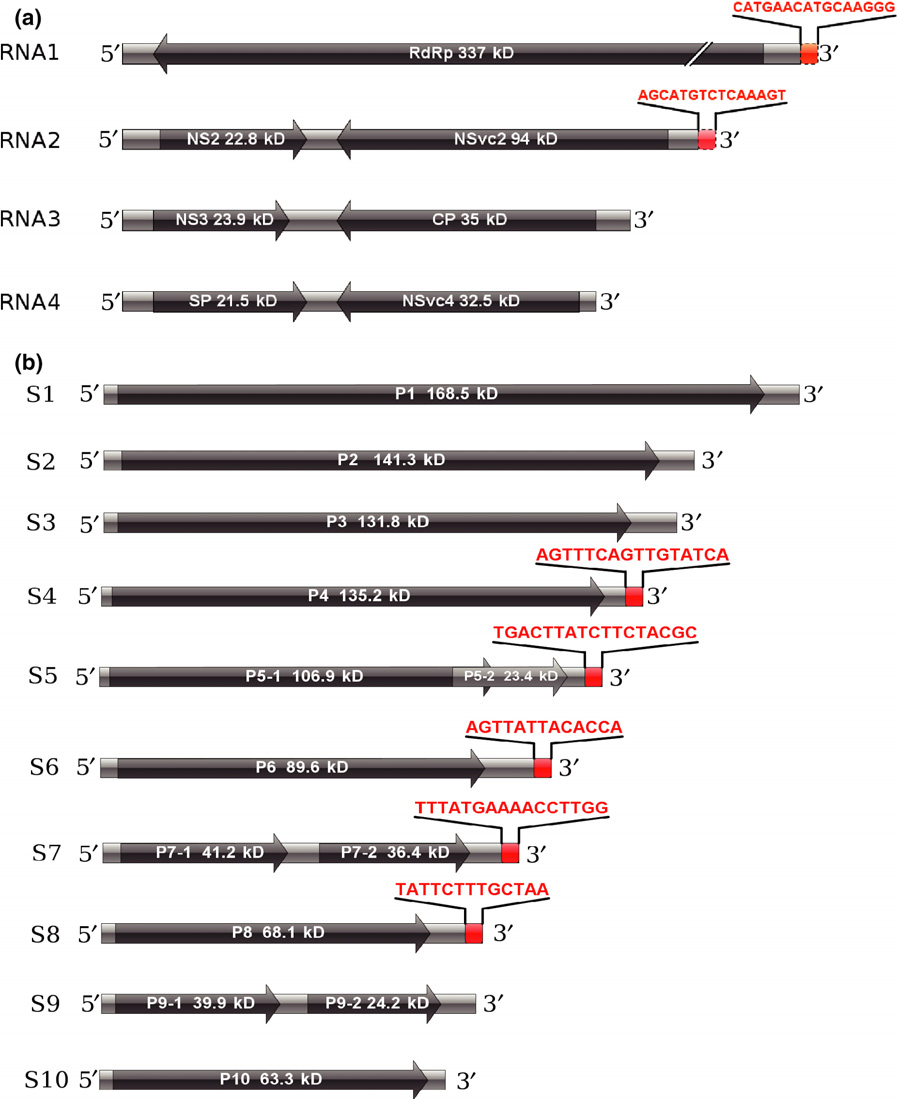
Schematic representation of the newly assembled *Rice stripe virus* (RSV) and *Southern rice black‐streaked dwarf virus* (SRBSDV) genomes. (a) The RSV genome that was obtained by *de novo* assembly and verified on the terminal sequences by 3′‐end reverse transcription‐polymerase chain reaction (RT‐PCR) amplification, Sanger sequencing and 5′‐rapid amplification of cDNA ends (5′‐RACE). The extended 3′‐terminal sequences in RNA1 and RNA2 are marked in red. (b) The *de novo* assembled sense strands of the SRBSDV genome. The sequencing data were retrieved from the work of Wang *et al*. (2016a). The extended 3′‐terminal sequences in RNA segments 4–8 are marked in red.

To exclude interference from the viral mRNA, the RSV ribonucleoprotein particles were purified using the anti‐CP monoclonal antibody from the JSHA isolate‐infected rice seedlings, and the viral genomic RNA was then extracted from the ribonucleoprotein particles for 3′‐terminal sequence determination. Filamentous ribonucleoprotein particles were clearly observed under transmission electron microscopy (Fig. 2a). Based on the touchdown RT‐PCR and Sanger sequencing results, the 16‐ and 15‐nt extensions at the 3′‐termini of RNA1 and RNA2, respectively, were detected in the RNA that was associated with the purified ribonucleoprotein particles (Fig. 2b), which strongly supports the notion that the 16‐ and 15‐nt extensions were present in the viral genomic RNA and not the viral mRNA. A schematic representation of the RSV genome that was obtained from the *de novo* assembly and verified on the terminal sequences is presented in Fig. 3(a).

### The 3′‐terminal extensions exist in different RSV isolates

The genomic 3′‐terminal extensions were observed in the RSV Jiangsu isolate (JSHA), which belongs to subtype I. To determine whether this phenomenon was common in different isolates of RSV, four Yunnan isolates (CXYH, CXYL, YXLC and KMXD) belonging to subtype II were tested (Fig. S3). The 16‐ and 15‐nt extensions at the 3′‐termini of RNA1 and RNA2 were successfully identified in the four Yunnan isolates from infected planthoppers and rice plants (Fig. 2c). Furthermore, these extension sequences were also verified from the purified RSV ribonucleoprotein of the KMXD isolate (Fig. 2b). Therefore, we conclude that the 16‐ and 15‐nt extensions at the 3′‐termini of RNA1 and RNA2 broadly exist in different isolates of RSV from different regions.

### The 3′‐terminal extensions are also found in the genome of SRBSDV

Next, we explored whether the existence of the 3′‐terminal extensions was a common phenomenon in plant RNA viruses. We searched the public databases and found the transcriptomic data from samples infected by SRBSDV. SRBSDV is a double‐stranded, multiple‐segment RNA virus that infects a variety of plants, such as rice and maize (Zhou *et al*., 2013). The genome of SRBSDV contains 10 segments that are named S1–S10 (Wang *et al*., 2016a). We *de novo* assembled the SRBSDV genome using the transcriptomic sequencing data that were generated from cDNA libraries constructed using random primers (Wang *et al*., 2016a). Compared with the published reference genome (from NC_014708.1 to NC_014717.1 in GenBank), the *de novo* assembled SRBSDV genome contained 3′‐terminal extension sequences of 13 to 17 nt in length on RNA segments 4–8, and each extension sequence was identical or nearly identical to a fragment in the upstream region of the same RNA segment (Figs 3b, S4). These characteristics are similar to those of RSV, which suggests that the existence of the 3′‐terminal extension sequences may be a general feature of RNA viruses.

### The 3′‐terminal extensions of RSV are enriched in the vector insects and gradually eliminated in the host plants

**Figure 4:**
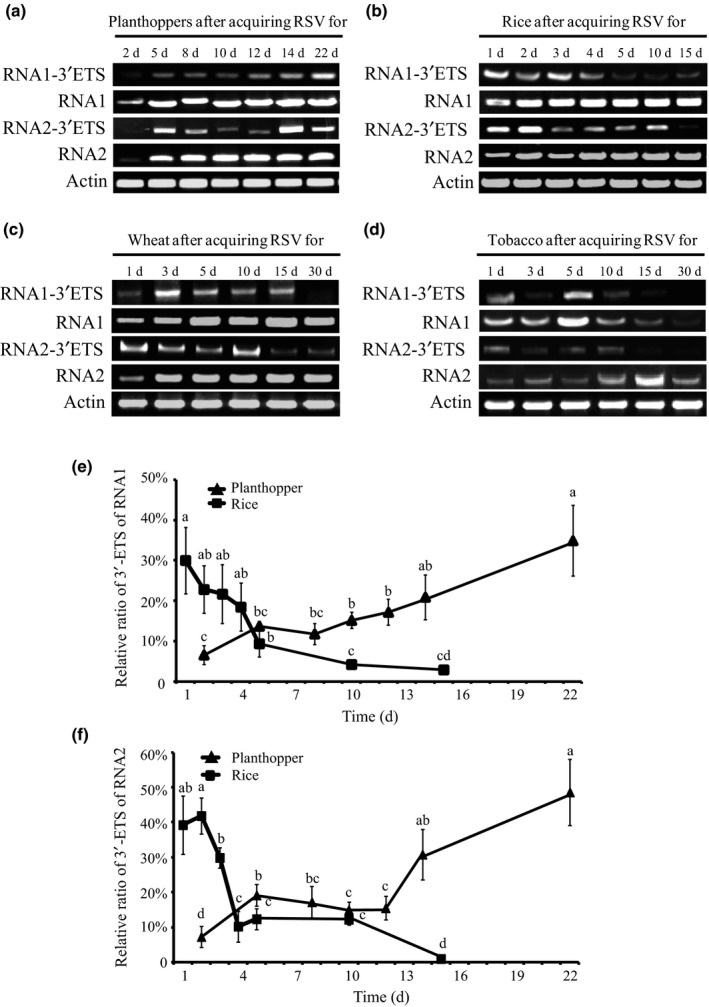
Accumulation of viral extended 3′‐terminal sequences in vector insects and host plants. Accumulations of RNA1 and RNA2 with the extended 3′‐terminal sequences in the small brown planthoppers (*Laodelphax striatellus*) (a), rice (*Oryza sativa* Huangjinqing) (b), wheat (*Triticum aestivum* jingdong 22) (c) and tobacco (*Nicotiana benthamiana*) (d) at different days after RSV infection. (e, f) Charts depicting the changes in the relative ratios of RNA1 and RNA2 to the extended 3′‐terminal sequences in planthoppers and rice plants, respectively. The extended 3′‐terminal sequences (3′‐ETS) were determined by amplifying the 3′‐non‐translated regions containing the extended terminal sequences using touchdown reverse transcription–polymerase chain reaction (RT‐PCR). The total amounts of viral RNA1 and RNA2 (based on amplification of fragments from the inner regions) and the transcripts of *actin* were used as internal controls. The ratios of RNA1 and RNA2 to the extended 3′‐terminal sequences were determined by associating the relative grey value of 3′‐ETS to that of the RNA levels of RNA1 or RNA2 using ImageJ based on Fig. 4a,b. The values are represented as the means ± SEs. The differences were statistically evaluated by one‐way ANOVA for multiple comparisons with spss 17.0 software. Different lowercase letters indicate significant differences at the *P* < 0.05 level.

To explore the dynamic alterations of the 3′‐terminal extensions in the vector insects and host plants, the amounts of RNA1 and RNA2 with the extended 3′‐terminal sequences were measured using touchdown RT‐PCR at different time points after inoculation with the JSHA isolate of RSV. The ratios of RNA1 and RNA2 with the extended 3′‐terminal sequences were determined by dividing the level of 3′‐non‐translated regions containing the extended terminal sequences by the total RNA level of RNA1 or RNA2. In the planthoppers, both RNA1 and RNA2 with the extended 3′‐terminal sequences were detected in low quantities at 2 d after inoculation (DAI) with plant‐derived RSV (Fig. 4a), with relative ratios of 6.6% ± 1.2% and 7.3% ± 1.5%, respectively (Fig. 4e,f). With increased virus incubation time, increasing amounts of RNA1 and RNA2 with the extended 3′‐terminal sequences were produced, and the relative ratios reached 34.9% ± 4.4% for the extended RNA1 and 48.6% ± 9.5% for the extended RNA2 at 22 DAI (Fig. 4a,e,f).

However, in the host plants that included rice, wheat and tobacco, the trends of variation in the RNA1 and RNA2 samples with extended 3′‐terminal sequences were different from those in the planthoppers (Fig. 4b–d). On RSV infection (1 DAI), the relative ratios of RNA1 and RNA2 with the extended 3′‐terminal sequences were > 20% and 30%, respectively, in the three plant species (Figs 4e,f, S5). These ratios gradually decreased with the time of viral incubation. In the late stage of RSV infection in rice (15 DAI), wheat (30 DAI) and tobacco (30 DAI), <3% of the RNA1 and RNA2 had the 3′‐terminal extensions (Figs 4e,f, S5). Therefore, the 16‐ and 15‐nt extensions at the 3′‐termini of RNA1 and RNA2 were enriched in vector insects, but gradually reduced in host plants during the infection process.

### Potential influence of the extended 3′‐terminal sequences on the replication of viral RNA1 and RNA2

**Figure 5:**
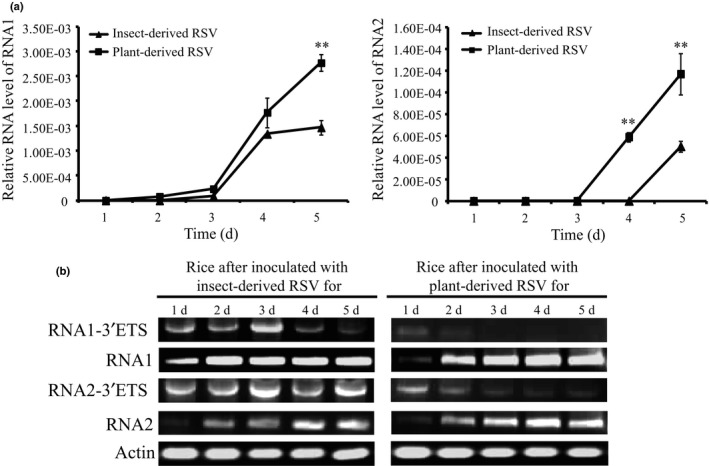
Replication levels of RNA1 and RNA2 in insect‐derived and plant‐derived *Rice stripe virus* (RSV) in rice (*Oryza sativa* Huangjinqing). (a) The RNA levels of viral RNA1 and RNA2 relative to that of rice *ubiquitin* in rice leaves within 5 d after inoculation with insect‐derived or plant‐derived RSV as measured by quantitative real‐time PCR. The values represent the means ± SEs from eight replicates. Student's *t*‐tests were performed to evaluate the differences between two means at the same time point. **, *P* < 0.01. (b) Accumulations of RNA1 and RNA2 with the extended 3′‐terminal sequences (3′‐ETS) in rice leaves after inoculation with insect‐ or plant‐derived RSV as measured with touchdown reverse transcription‐polymerase chain reaction (RT‐PCR). The total amounts of viral RNA1 and RNA2 and the transcripts of rice *actin* were amplified as internal controls.

The panhandle structure formed by the 3′‐ and 5′‐terminal regions is thought to play an important role in RNA replication in viruses, such as the Bunyamwera virus, the prototypic arenavirus lymphocytic choriomeningitis virus, influenza virus and flaviviruses (Fodor *et al*., 1994; Flick & Hobom, 1999; Perez & de la Torre, 2003; Barr & Wertz, 2004). The 3′‐terminal extensions identified in the RSV RNA1 and RNA2 would affect the replication of these two RNAs. To test this hypothesis, the replication levels of RNA1 and RNA2 from insect‐derived or plant‐derived RSVs were quantified and compared in rice plants within 5 d of infection, because the ratios of the 3′‐terminal extensions of insect‐derived RSV declined to < 10% in rice at 5 DAI (Fig. 4b,e,f). One day after microinjection of either insect‐ or plant‐derived RSV, the level of RNA1 was not detectable in plants. After 2 DAI, the amount of RNA1 increased, but the accumulation of RNA1 was higher when the inoculation was performed with plant‐derived RSV than when it was performed with insect‐derived RSV, and the difference became statistically significant at 5 DAI (Fig. 5a). Similarly, the level of RNA2 was not detectable within the first 3 d of infection. After 4 DAI, the accumulation of RNA2 was significantly higher when the plants were inoculated with plant‐derived RSV (Fig. 5a). The accumulated levels of the extended 3′‐terminal sequences of RNA1 and RNA2 from the two types of RSV were also simultaneously measured. The amount of the 3′‐terminal extensions obviously decreased in rice with time. Moreover, fewer 3′‐terminal extensions of RNA1 and RNA2 existed in plant‐derived RSVs than in insect‐derived RSVs (Fig. 5b). The correlation between the amount of 3′‐terminal extensions and the replication level of the viral RNAs suggests that the 16‐ and 15‐nt extensions at the 3′‐termini may be detrimental to the replication of viral RNA1 and RNA2 in the host plant.

## Discussion

As a result of the error‐prone replication characteristics of RdRp, RNA viruses usually comprise heterogeneous intra‐host populations. Different genetic divergences occur in intra‐host populations of persistent‐propagative plant RNA viruses when they adapt to their vector insects and host plants. Here, we successfully assembled the RSV genome as it exists in its vector insect, i.e. the small brown planthopper, and analysed the genomic variations that occurred during the viral transmission between the vector insect and host plant. Although many plant virus genomes from field isolates have been reported, to the best of our knowledge, this is the first report of the determination of a plant virus genome through *de novo* assembly from its vector insect with a comparison of the vector‐derived virus genome and the host‐derived virus genome.

The most outstanding finding of the present study is that the rotation between the vector insect and the host plant imposes a selective pressure on the RSV genome at the 3′‐termini. Why have variations in the genomic 3′‐termini not been observed in previous studies of various RSV field isolates from Japan, Korea and China in the past 20 yr (Zhang *et al*., 2007; Jonson *et al*., 2009a,b; Wei *et al*., 2009; Huang *et al*., 2013, 2015)? The 5′‐ and 3′‐terminal sequences of the four RNA segments were first determined in RSV T isolates from infected maize through two‐dimensional mobility shift analyses of the first 18 bases, followed by the application of chemical modification and enzymatic methods for longer sequences (Takahashi *et al*., 1990). Different lengths of RNAs have different two‐dimensional mobility shifts. Based on the present study, RNA1 and RNA2 with 3′‐extensions accounted for only 3% of the total RNA segments in the late stage of RSV infection in host plants. Therefore, it is likely that only the predominant types of terminal sequences were measured in these previous studies. Since 1990, two main methods have been used for RSV genome re‐sequencing, i.e. cDNA library construction with specific primers designed according to the highly conserved 3′‐terminal regions of the four viral RNA segments, and RT‐PCR with specific primer pairs that target specific regions in the genome (Zhu *et al*., 1991, 1992; Takahashi *et al*., 1993; Toriyama *et al*., 1994; Zhang *et al*., 2007; Jonson *et al*., 2009a,b; Wei *et al*., 2009; Huang *et al*., 2015). Consequently, all registered genome sequences of RSV isolates in GenBank, regardless of whether they are from infected maize or rice plants, are identical in their terminal sequences and do not contain the 3′‐terminal sequences that were discovered in this study.

The extensions at the viral 3′‐termini may affect RSV replication. Without the 16‐ and 15‐nt extensions at the 3′‐termini, the 5′‐ and 3′‐termini of RNA1 and RNA2 are strongly self‐complementary, which enables the formation of a panhandle structure or a so‐called stem‐loop structure. The 3′‐terminal extensions impair the formation of the panhandle structures of RNA1 and RNA2. During the initiation of negative‐ or positive‐strand RNA synthesis, the stem‐loop structures resulting from the 3′‐ and 5′‐terminal regions are thought to play an important role in RNA replication in viruses, such as the Bunyamwera virus, the prototypic arenavirus lymphocytic choriomeningitis virus, influenza virus and flaviviruses (Fodor *et al*., 1994; Flick & Hobom, 1999; Perez & de la Torre, 2003; Barr & Wertz, 2004). The amount of RSV with the 3′‐terminal extensions increased with time in the vector insect and decreased or was eventually nearly eliminated in the host plant. This pattern is probably one of the reasons, from a structural perspective, for the limited replication of RSV in the vector insect and the uncontrolled replication in the host plant.

Our findings with regard to how the genome of a plant virus adapts to its vector insect and host plant share some commonalities with the adaptation of arboviruses to their vector insects and humans. Changes in viral RNAs have been thoroughly investigated in mosquito‐borne human pathogens, such as dengue virus and Zika virus, during their alternation between mosquitoes and humans (Liu *et al*., 2013; Villordo & Gamarnik, 2013; de Borba *et al*., 2015; Sessions *et al*., 2015; Wang *et al*., 2016b). The compositions of viral populations in mosquito and mammalian cells are quite different, and the enrichment and function of specific variants are host‐specific. For example, more variants in the 3′‐untranslated region of dengue virus that contains point mutations or deletions have been detected in mosquito cells than in mammalian cells. The mutations in the 3′‐untranslated region are beneficial for viral replication in mosquito cells, whereas they are detrimental in mammalian cells (Villordo *et al*., 2015). Some specific mutations in the 3′‐untranslated region of dengue virus produce different species of subgenomic flavivirus RNAs that play key roles in immune responses in vertebrate and invertebrate cells after viral infection (Filomatori *et al*., 2017).

Taken together, our present work reveals the 3′‐terminal sequences in the genome of a persistent‐propagative plant virus that have been overlooked for a long time, as well as the genomic variations in the virus populations in the rotation between vector insects and host plants. These variations appear to be present in many multiple‐segment RNA plant viruses. Further investigations are needed to understand whether the 3′‐terminal extensions account for different replication strategies of the virus when facing different immune pressures in the vector insect and host plant.

## Author contributions

W.Z. performed the experiments. Z.X. performed the genomic analysis. X.Z and M.Y. analysed the data. F.C., R.L. and L.K. designed the experiments. W.Z., Z.X., F.C., R.L. and L.K. wrote the manuscript. W.Z. and Z.X. contributed equally to this work.

## Supporting information

Please note: Wiley Blackwell are not responsible for the content or functionality of any Supporting Information supplied by the authors. Any queries (other than missing material) should be directed to the *New Phytologist* Central Office.

**Fig. S1** The 3′‐terminal sequences of *Rice stripe virus* (RSV) RNA1 to RNA4 verified using touchdown RT‐PCR and Sanger sequencing.
**Fig. S2** The 5′‐terminal sequences of *Rice stripe virus* (RSV) RNA1 to RNA4 verified using 5′‐rapid amplification of cDNA ends experiments and Sanger sequencing.
**Fig. S3** Phylogenetic analysis of the *Rice stripe virus* (RSV) isolates based on the *CP* gene.
**Fig. S4** Comparison of the 3′‐terminal 300 nucleotides of RNA segments 4–8 of *Southern rice black‐streaked dwarf virus* (SRBSDV) between the published reference genome and our *de novo* assembled genome.
**Fig. S5** The trends in the changes of the relative ratios of RNA1 (a) and RNA2 (b) to the extended 3′‐terminal sequences in wheat (*Triticum aestivum* jingdong 22) and tobacco (*Nicotiana benthamiana*).
**Table S1** Primers used in this study
**Table S2** Summary statistics of the RNA sequencing data from the *Rice stripe virus* (RSV)‐infected small brown planthoppers (*Laodelphax striatellus*) and rice (*Oryza sativa* Huangjinqing)
**Table S3** Coverages and depths of the newly assembled genome by *Rice stripe virus* (RSV) reads from different samples
