# Supplementary material for: Genomic variations in the 3′‐termini of Rice stripe virus in the rotation between vector insect and host plant
Source: New Phytol. 2018 Jun 8;219(3):1085–96. doi: 10.1111/nph.15246 (PMC6055815; doi:10.1111/nph.15246)
Supplement: Supplementary file 1 — Fig. S1 The 3′‐terminal sequences of Rice stripe virus (RSV) RNA1 to RNA4 verified using touchdown RT‐PCR and Sanger sequencing. Fig. S2 The 5′‐terminal sequences of Rice stripe virus (RSV) RNA1 to RNA4 verified using 5′‐rapid amplification of cDNA ends experiments and Sanger sequencing. Fig. S3 Phylogenetic analysis of the Rice stripe virus (RSV) isolates based on the CP gene. Fig. S4 Comparison of the 3′‐terminal 300 nucleotides of RNA segments 4–8 of Southern rice black‐streaked dwarf virus (SRBSDV) between the published reference genome and our de novo assembled genome. Fig. S5 The trends in the changes of the relative ratios of RNA1 (a) and RNA2 (b) to the extended 3′‐terminal sequences in wheat (Triticum aestivum jingdong 22) and tobacco (Nicotiana benthamiana). Table S1 Primers used in this study Table S2 Summary statistics of the RNA sequencing data from the Rice stripe virus (RSV)‐infected small brown planthoppers (Laodelphax striatellus) and rice (Oryza sativa Huangjinqing) Table S3 Coverages and depths of the newly assembled genome by Rice stripe virus (RSV) reads from different samples [file NPH-219-1085-s001.pdf]

New Phytologist Supporting Information

Article title: **Genomic variations in the 3'-termini of *Rice stripe virus* in the rotation between vector insect and host plant**

Authors: Wan Zhao, Zhongtian Xu, Xiaoming Zhang, Meiling Yang, Le Kang, Renyi Liu, Feng Cui

Article acceptance date: 01 May 2018

The following Supporting Information is available for this article:

Fig. S1 The 3'-terminal sequences of *Rice stripe virus* (RSV) RNA1 to RNA4 verified using touchdown RT-PCR and Sanger sequencing.

Fig. S2 The 5'-terminal sequences of *Rice stripe virus* (RSV) RNA1 to RNA4 verified using 5'-rapid amplification of cDNA ends experiments and Sanger sequencing.

Fig. S3 Phylogenetic analysis of the *Rice stripe virus* (RSV) isolates based on the *CP* gene.

Fig. S4 Comparison of the 3'-terminal 300 nucleotides of RNA segments 4 through 8 of *Southern rice black-streaked dwarf virus* (SRBSDV) between the published reference genome and our *de novo* assembled genome.

Fig. S5 The trends in the changes of the relative ratios of RNA1 (a) and RNA2 (b) to the extended 3'-terminal sequences in (*Triticum aestivum* jingdong 22) and tobacco (*Nicotiana benthamiana*).

Table S1 Primers used in this study

Table S2 Summary statistics of the RNA sequencing data from the *Rice stripe virus* (RSV)-infected small brown planthoppers (*Laodelphax striatellus*) and rice (*Oryza sativa* Huangjinqing)

Table S3 Coverages and depths of the newly assembled genome by *Rice stripe virus* (RSV) reads from different samples

Fig. S1 The 3'-terminal sequences of *Rice stripe virus* (RSV) RNA1 to RNA4 verified using touchdown RT-PCR and Sanger sequencing. Fragments of 784-nt, 521-nt, 793-nt, or 795-nt at the 3'-termini of the four segments were amplified from the RSV-infected rice plants or small brown planthoppers. The arrows indicate the positions of the primers. The sequences of the four clones are shown for each sample.

RNA1

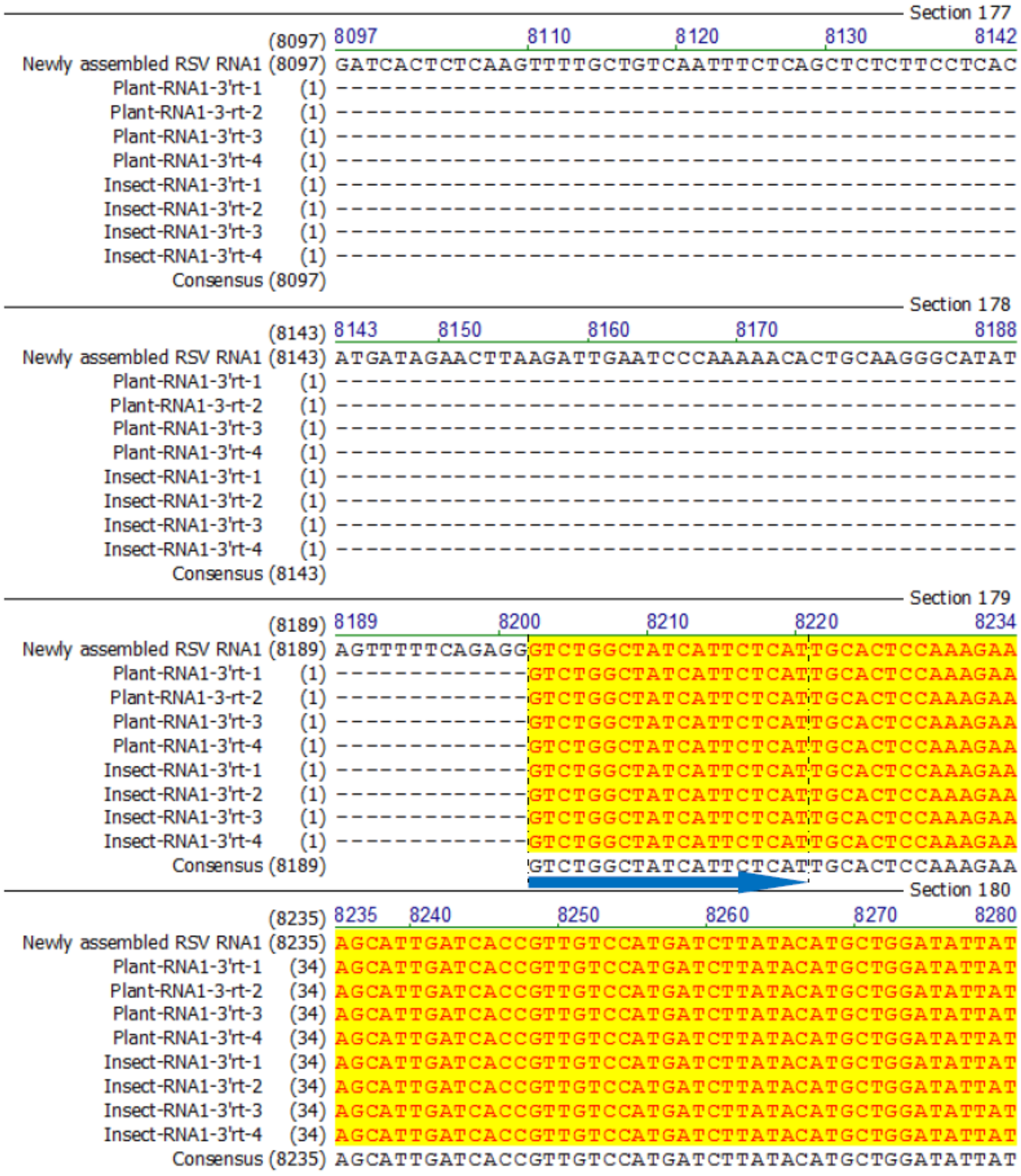

```
                                                                     Section 181
                         (8281) 8281        8290          8300          8310                 8326
Newly assembled RSV RNA1 (8281) GCTACCTATCAATTCATCCACATTACTAAAGCTCTTGTGTCCAGAA
        Plant-RNA1-3'rt-1  (80) GCTACCTATCAATTCATCCACATTACTAAAGCTCTTGTGTCCAGAA
        Plant-RNA1-3-rt-2  (80) GCTACCTATCAATTCATCCACATTACTAAAGCTCTTGTGTCCAGAA
        Plant-RNA1-3'rt-3  (80) GCTACCTATCAATTCATCCACATTACTAAAGCTCTTGTGTCCAGAA
        Plant-RNA1-3'rt-4  (80) GCTACCTATCAATTCATCCACATTACTAAAGCTCTTGTGTCCAGAA
       Insect-RNA1-3'rt-1  (80) GCTACCTATCAATTCATCCACATTACTAAAGCTCTTGTGTCCAGAA
       Insect-RNA1-3'rt-2  (80) GCTACCTATCAATTCATCCACATTACTAAAGCTCTTGTGTCCAGAA
       Insect-RNA1-3'rt-3  (80) GCTACCTATCAATTCATCCACATTACTAAAGCTCTTGTGTCCAGAA
       Insect-RNA1-3'rt-4  (80) GCTACCTATCAATTCATCCACATTACTAAAGCTCTTGTGTCCAGAA
                Consensus (8281) GCTACCTATCAATTCATCCACATTACTAAAGCTCTTGTGTCCAGAA
```

```
                                                                     Section 182
                         (8327) 8327              8340          8350          8360          8372
Newly assembled RSV RNA1 (8327) AGGCTATCAGATACTATAGGCTTGTAAACAGATGCATGCCACAAGG
        Plant-RNA1-3'rt-1 (126) AGGCTATCAGATACTATAGGCTTGTAAACAGATGCATGCCACAAGG
        Plant-RNA1-3-rt-2 (126) AGGCTATCAGATACTATAGGCTTGTAAACAGATGCATGCCACAAGG
        Plant-RNA1-3'rt-3 (126) AGGCTATCAGATACTATAGGCTTGTAAACAGATGCATGCCACAAGG
        Plant-RNA1-3'rt-4 (126) AGGCTATCAGATACTATAGGCTTGTAAACAGATGCATGCCACAAGG
       Insect-RNA1-3'rt-1 (126) AGGCTATCAGATACTATAGGCTTGTAAACAGATGCATGCCACAAGG
       Insect-RNA1-3'rt-2 (126) AGGCTATCAGATACTATAGGCTTGTAAACAGATGCATGCCACAAGG
       Insect-RNA1-3'rt-3 (126) AGGCTATCAGATACTATAGGCTTGTAAACAGATGCATGCCACAAGG
       Insect-RNA1-3'rt-4 (126) AGGCTATCAGATACTATAGGCTTGTAAACAGATGCATGCCACAAGG
                Consensus (8327) AGGCTATCAGATACTATAGGCTTGTAAACAGATGCATGCCACAAGG
```

```
                                                                     Section 183
                         (8373) 8373     8380          8390          8400                 8418
Newly assembled RSV RNA1 (8373) ATATGGATTCACAGTCTTCCACTGGCATTCTCAAGAATTCTTCAAT
        Plant-RNA1-3'rt-1 (172) ATATGGATTCACAGTCTTCCACTGGCATTCTCAAGAATTCTTCAAT
        Plant-RNA1-3-rt-2 (172) ATATGGATTCACAGTCTTCCACTGGCATTCTCAAGAATTCTTCAAT
        Plant-RNA1-3'rt-3 (172) ATATGGATTCACAGTCTTCCACTGGCATTCTCAAGAATTCTTCAAT
        Plant-RNA1-3'rt-4 (172) ATATGGATTCACAGTCTTCCACTGGCATTCTCAAGAATTCTTCAAT
       Insect-RNA1-3'rt-1 (172) ATATGGATTCACAGTCTTCCACTGGCATTCTCAAGAATTCTTCAAT
       Insect-RNA1-3'rt-2 (172) ATATGGATTCACAGTCTTCCACTGGCATTCTCAAGAATTCTTCAAT
       Insect-RNA1-3'rt-3 (172) ATATGGATTCACAGTCTTCCACTGGCATTCTCAAGAATTCTTCAAT
       Insect-RNA1-3'rt-4 (172) ATATGGATTCACAGTCTTCCACTGGCATTCTCAAGAATTCTTCAAT
                Consensus (8373) ATATGGATTCACAGTCTTCCACTGGCATTCTCAAGAATTCTTCAAT
```

```
                                                                     Section 184
                         (8419) 8419           8430          8440          8450              8464
Newly assembled RSV RNA1 (8419) TTTTGATCTGAGCTTTACTTTATGGTCAAAATCTGTCTCAAGTGAC
        Plant-RNA1-3'rt-1 (218) TTTTGATCTGAGCTTTACTTTATGGTCAAAATCTGTCTCAAGTGAC
        Plant-RNA1-3-rt-2 (218) TTTTGATCTGAGCTTTACTTTATGGTCAAAATCTGTCTCAAGTGAC
        Plant-RNA1-3'rt-3 (218) TTTTGATCTGAGCTTTACTTTATGGTCAAAATCTGTCTCAAGTGAC
        Plant-RNA1-3'rt-4 (218) TTTTGATCTGAGCTTTACTTTATGGTCAAAATCTGTCTCAAGTGAC
       Insect-RNA1-3'rt-1 (218) TTTTGATCTGAGCTTTACTTTATGGTCAAAATCTGTCTCAAGTGAC
       Insect-RNA1-3'rt-2 (218) TTTTGATCTGAGCTTTACTTTATGGTCAAAATCTGTCTCAAGTGAC
       Insect-RNA1-3'rt-3 (218) TTTTGATCTGAGCTTTACTTTATGGTCAAAATCTGTCTCAAGTGAC
       Insect-RNA1-3'rt-4 (218) TTTTGATCTGAGCTTTACTTTATGGTCAAAATCTGTCTCAAGTGAC
                Consensus (8419) TTTTGATCTGAGCTTTACTTTATGGTCAAAATCTGTCTCAAGTGAC
```

```
                                                                                   Section 185
                          (8465) 8465 8470      8480      8490      8500      8510
Newly assembled RSV RNA1 (8465) TTGAAGTGGTAATTCCCATAGTTTCTAATTCTAATAGTACTCTCAA
       Plant-RNA1-3'rt-1  (264) TTGAAGTGGTAATTCCCATAGTTTCTAATTCTAATAGTACTCTCAA
       Plant-RNA1-3-rt-2  (264) TTGAAGTGGTAATTCCCATAGTTTCTAATTCTAATAGTACTCTCAA
       Plant-RNA1-3'rt-3  (264) TTGAAGTGGTAATTCCCATAGTTTCTAATTCTAATAGTACTCTCAA
       Plant-RNA1-3'rt-4  (264) TTGAAGTGGTAATTCCCATAGTTTCTAATTCTAATAGTACTCTCAA
      Insect-RNA1-3'rt-1  (264) TTGAAGTGGTAATTCCCATAGTTTCTAATTCTAATAGTACTCTCAA
      Insect-RNA1-3'rt-2  (264) TTGAAGTGGTAATTCCCATAGTTTCTAATTCTAATAGTACTCTCAA
      Insect-RNA1-3'rt-3  (264) TTGAAGTGGTAATTCCCATAGTTTCTAATTCTAATAGTACTCTCAA
      Insect-RNA1-3'rt-4  (264) TTGAAGTGGTAATTCCCATAGTTTCTAATTCTAATAGTACTCTCAA
               Consensus (8465) TTGAAGTGGTAATTCCCATAGTTTCTAATTCTAATAGTACTCTCAA
                                                                                   Section 186
                          (8511) 8511     8520      8530      8540           8556
Newly assembled RSV RNA1 (8511) ACTCAGCACCATAAATAGTCTTAAACAACTTCCCACCAACTGTCTG
       Plant-RNA1-3'rt-1  (310) ACTCAGCACCATAAATAGTCTTAAACAACTTCCCACCAACTGTCTG
       Plant-RNA1-3-rt-2  (310) ACTCAGCACCATAAATAGTCTTAAACAACTTCCCACCAACTGTCTG
       Plant-RNA1-3'rt-3  (310) ACTCAGCACCATAAATAGTCTTAAACAACTTCCCACCAACTGTCTG
       Plant-RNA1-3'rt-4  (310) ACTCAGCACCATAAATAGTCTTAAACAACTTCCCACCAACTGTCTG
      Insect-RNA1-3'rt-1  (310) ACTCAGCACCATAAATAGTCTTAAACAACTTCCCATCAACTGTCTG
      Insect-RNA1-3'rt-2  (310) ACTCAGCACCATAAATAGTCTTAAACAACTTCCCATCAACTGTCTG
      Insect-RNA1-3'rt-3  (310) ACTCAGCACCATAAATAGTCTTAAACAACTTCCCATCAACTGTCTG
      Insect-RNA1-3'rt-4  (310) ACTCAGCACCATAAATAGTCTTAAACAACTTCCCATCAACTGTCTG
               Consensus (8511) ACTCAGCACCATAAATAGTCTTAAACAACTTCCCACCAACTGTCTG
                                                                                   Section 187
                          (8557) 8557         8570      8580      8590      8602
Newly assembled RSV RNA1 (8557) GTAAGCAATTATCTTAATTCTCAATATTATAGCCATAAACCCAATT
       Plant-RNA1-3'rt-1  (356) GTAAGCAATTATCTTAATTCTCAATATTATAGCCATAAACCCAATT
       Plant-RNA1-3-rt-2  (356) GTAAGCAATTATCTTAATTCTCAATATTATAGCCATAAACCCAATT
       Plant-RNA1-3'rt-3  (356) GTAAGCAATTATCTTAATTCTCAATATTATAGCCATAAACCCAATT
       Plant-RNA1-3'rt-4  (356) GTAAGCAATTATCTTAATTCTCAATATTATAGCCATAAACCCAATT
      Insect-RNA1-3'rt-1  (356) GTAAGCAATTATCTTAATTCTCAATATTATAGCCATAAACCCAATT
      Insect-RNA1-3'rt-2  (356) GTAAGCAATTATCTTAATTCTCAATATTATAGCCATAAACCCAATT
      Insect-RNA1-3'rt-3  (356) GTAAGCAATTATCTTAATTCTCAATATTATAGCCATAAACCCAATT
      Insect-RNA1-3'rt-4  (356) GTAAGCAATTATCTTAATTCTCAATATTATAGCCATAAACCCAATT
               Consensus (8557) GTAAGCAATTATCTTAATTCTCAATATTATAGCCATAAACCCAATT
                                                                                   Section 188
                          (8603) 8603   8610      8620      8630               8648
Newly assembled RSV RNA1 (8603) GCTATATCTTCACCCCAGGCTGATGAGTCATTTGGATCAAGCTGCT
       Plant-RNA1-3'rt-1  (402) GCTATATCTTCACCCCAGGCTGATGAGTCATTTGGATCAAGCTGCT
       Plant-RNA1-3-rt-2  (402) GCTATATCTTCACCCCAGGCTGATGAGTCATTTGGATCAAGCTGCT
       Plant-RNA1-3'rt-3  (402) GCTATATCTTCACCCCAGGCTGATGAGTCATTTGGATCAAGCTGCT
       Plant-RNA1-3'rt-4  (402) GCTATATCTTCACCCCAGGCTGATGAGTCATTTGGATCAAGCTGCT
      Insect-RNA1-3'rt-1  (402) GCTATATCTTCACCCCAGGCTGATGAGTCATTTGGATCAAGCTGCT
      Insect-RNA1-3'rt-2  (402) GCTATATCTTCACCCCAGGCTGATGAGTCATTTGGATCAAGCTGCT
      Insect-RNA1-3'rt-3  (402) GCTATATCTTCACCCCAGGCTGATGAGTCATTTGGATCAAGCTGCT
      Insect-RNA1-3'rt-4  (402) GCTATATCTTCACCCCAGGCTGATGAGTCATTTGGATCAAGCTGCT
               Consensus (8603) GCTATATCTTCACCCCAGGCTGATGAGTCATTTGGATCAAGCTGCT
```

Section 189

	(8649)	8649 8660 8670 8680 8694
Newly assembled RSV RNA1	(8649)	TCATAAGGCTCAGTTGCATTTCTTGATCCAGCTGGCAGATAGCTGG
Plant-RNA1-3'rt-1	(448)	TCATAAGGCTCAGTTGCATTTCTTGATCCAGCTGGCAGATAGCTGG
Plant-RNA1-3-rt-2	(448)	TCATAAGGCTCAGTTGCATTTCTTGATCCAGCTGGCAGATAGCTGG
Plant-RNA1-3'rt-3	(448)	TCATAAGGCTCAGTTGCATTTCTTGATCCAGCTGGCAGATAGCTGG
Plant-RNA1-3'rt-4	(448)	TCATAAGGCTCAGTTGCATTTCTTGATCCAGCTGGCAGATAGCTGG
Insect-RNA1-3'rt-1	(448)	TCATAAGGCTCAGTTGCATTTCTTGATCCAGCTGGCAGATAGCTGG
Insect-RNA1-3'rt-2	(448)	TCATAAGGCTCAGTTGCATTTCTTGATCCAGCTGGCAGATAGCTGG
Insect-RNA1-3'rt-3	(448)	TCATAAGGCTCAGTTGCATTTCTTGATCCAGCTGGCAGATAGCTGG
Insect-RNA1-3'rt-4	(448)	TCATAAGGCTCAGTTGCATTTCTTGATCCAGCTGGCAGATAGCTGG
Consensus	(8649)	TCATAAGGCTCAGTTGCATTTCTTGATCCAGCTGGCAGATAGCTGG

Section 190

	(8695)	8695 8700 8710 8720 8730 8740
Newly assembled RSV RNA1	(8695)	ATTTGATCGTAGCTTTATCATAAATTCAGTGGTGCGAGAGTTCTCC
Plant-RNA1-3'rt-1	(494)	ATTTGATCGTAGCTTTATCATAAATTCAGTGGTGCGAGAGTTCTCC
Plant-RNA1-3-rt-2	(494)	ATTTGATCGTAGCTTTATCATAAATTCAGTGGTGCGAGAGTTCTCC
Plant-RNA1-3'rt-3	(494)	ATTTGATCGTAGCTTTATCATAAATTCAGTGGTGCGAGAGTTCTCC
Plant-RNA1-3'rt-4	(494)	ATTTGATCGTAGCTTTATCATAAATTCAGTGGTGCGAGAGTTCTCC
Insect-RNA1-3'rt-1	(494)	ATTTGATCGTAGCTTTATCATAAATTCAGTGGTGCGAGAGTTCTCC
Insect-RNA1-3'rt-2	(494)	ATTTGATCGTAGCTTTATCATAAATTCAGTGGTGCGAGAGTTCTCC
Insect-RNA1-3'rt-3	(494)	ATTTGATCGTAGCTTTATCATAAATTCAGTGGTGCGAGAGTTCTCC
Insect-RNA1-3'rt-4	(494)	ATTTGATCGTAGCTTTATCATAAATTCAGTGGTGCGAGAGTTCTCC
Consensus	(8695)	ATTTGATCGTAGCTTTATCATAAATTCAGTGGTGCGAGAGTTCTCC

Section 191

	(8741)	8741 8750 8760 8770 8786
Newly assembled RSV RNA1	(8741)	TTGCTCAGGCCCATACTATATAGTATGGAATGATAAAGACAAAATC
Plant-RNA1-3'rt-1	(540)	TTGCTCAGGCCCATACTATATAGTATGGAATGATAAAGACAAAATC
Plant-RNA1-3-rt-2	(540)	TTGCTCAGGCCCATACTATATAGTATGGAATGATAAAGACAAAATC
Plant-RNA1-3'rt-3	(540)	TTGCTCAGGCCCATACTATATAGTATGGAATGATAAAGACAAAATC
Plant-RNA1-3'rt-4	(540)	TTGCTCAGGCCCATACTATATAGTATGGAATGATAAAGACAAAATC
Insect-RNA1-3'rt-1	(540)	TTGCTCAGGCCCATACTATATAGTATGGAATGATAAAGACAAAATC
Insect-RNA1-3'rt-2	(540)	TTGCTCAGGCCCATACTATATAGTATGGAATGATAAAGACAAAATC
Insect-RNA1-3'rt-3	(540)	TTGCTCAGGCCCATACTATATAGTATAGAATGATAAAGACAAAATC
Insect-RNA1-3'rt-4	(540)	TTGCTCAGGCCCATACTATATAGTATGGAATGATAAAGACAAAATC
Consensus	(8741)	TTGCTCAGGCCCATACTATATAGTATGGAATGATAAAGACAAAATC

Section 192

	(8787)	8787 8800 8810 8820 8832
Newly assembled RSV RNA1	(8787)	CATCACCTTTGACATCTGTTTCTTCCAACTCTTCTATCTCCTGCCA
Plant-RNA1-3'rt-1	(586)	CATCACCTTTGACATCTGTTTCTTCCAACTCTTCTATCTCCTGCCA
Plant-RNA1-3-rt-2	(586)	CATCACCTTTGACATCTGTTTCTTCCAACTCTTCTATCTCCTGCCA
Plant-RNA1-3'rt-3	(586)	CATCACCTTTGACATCTGTTTCTTCCAACTCTTCTATCTCCTGCCA
Plant-RNA1-3'rt-4	(586)	CATCACCTTTGACATCTGTTTCTTCCAACTCTTCTATCTCCTGCCA
Insect-RNA1-3'rt-1	(586)	CATCACCTTTGACATCTGTTTCTTCCAACTCTTCTATCTCCTGCCA
Insect-RNA1-3'rt-2	(586)	CATCACCTTTGACATCTGTTTCTTCCAACTCTTCTATCTCCTGCCA
Insect-RNA1-3'rt-3	(586)	CATCACCTTTGACATCTGTTTCTTCCAACTCTTCTATCTCCTGCCA
Insect-RNA1-3'rt-4	(586)	CATCACCTTTGACATCTGTTTCTTCCAACTCTTCTATCTCCTGCCA
Consensus	(8787)	CATCACCTTTGACATCTGTTTCTTCCAACTCTTCTATCTCCTGCCA

```
                                                                          Section 193
                        (8833) 8833      8840          8850           8860                8878
Newly assembled RSV RNA1 (8833) ATCAACTTCATGATACCCCGCCAACAGTTCATAAGACCTGCCATGA
      Plant-RNA1-3'rt-1  (632) ATCAACTTCATGATACCCCGCCAACAGTTCATAAGACCTGCCATGA
      Plant-RNA1-3-rt-2  (632) ATCAACTTCATGATACCCCGCCAACAGTTCATAAGACCTGCCATGA
      Plant-RNA1-3'rt-3  (632) ATCAACTTCATGATACCCCGCCAACAGTTCATAAGACCTGCCATGA
      Plant-RNA1-3'rt-4  (632) ATCAACTTCATGATACCCCGCCAACAGTTCATAAGACCTGCCATGA
     Insect-RNA1-3'rt-1  (632) ATCAACTTCATGATACCCCGCCAACAGTTCATAAGACCTGCCATGA
     Insect-RNA1-3'rt-2  (632) ATCAACTTCATGATACCCCGCCAACAGTTCATAAGACCTGCCATGA
     Insect-RNA1-3'rt-3  (632) ATCAACTTCATGATACCCCGCCAACAGTTCATAAGACCTGCCATGA
     Insect-RNA1-3'rt-4  (632) ATCAACTTCATGATACCCCGCCAACAGTTCATAAGACCTGCCATGA
             Consensus (8833) ATCAACTTCATGATACCCCGCCAACAGTTCATAAGACCTGCCATGA
                                                                          Section 194
                        (8879) 8879           8890         8900          8910            8924
Newly assembled RSV RNA1 (8879) ACATGCAAGGGTATAACGAGAGGTGGTGTCGTCATCCTTCAATTCT
      Plant-RNA1-3'rt-1  (678) ACATGCAAGGGTATAACGAGAGGTGGTGTCGTCATCCTTCAATTCT
      Plant-RNA1-3-rt-2  (678) ACATGCAAGGGTATAACGAGAGGTGGTGTCGTCATCCTTCAATTCT
      Plant-RNA1-3'rt-3  (678) ACATGCAAGGGTATAACGAGAGGTGGTGTCGTCATCCTTCAATTCT
      Plant-RNA1-3'rt-4  (678) ACATGCAAGGGTATAACGAGAGGTGGTGTCGTCATCCTTCAATTCT
     Insect-RNA1-3'rt-1  (678) ACATGCAAGGGTATAACGAGAGGTGGTGTCGTCATCCTTCAATTCT
     Insect-RNA1-3'rt-2  (678) ACATGCAAGGGTATAACGAGAGGTGGTGTCGTCATCCTTCAATTCT
     Insect-RNA1-3'rt-3  (678) ACATGCAAGG-TATAACGAGAGGTGGTGTCGTCATCCTTCAATTCT
     Insect-RNA1-3'rt-4  (678) ACATGCAAGGGTATAACGAGAGGTGGTGTCGTCATCCTTCAATTCT
             Consensus (8879) ACATGCAAGGGTATAACGAGAGGTGGTGTCGTCATCCTTCAATTCT
                                                                          Section 195
                        (8925) 8925  8930          8940         8950          8960        8970
Newly assembled RSV RNA1 (8925) TTTGTGGAAAACAAAATCAAAATTATTCTTCCTCTGACTATGTGTC
      Plant-RNA1-3'rt-1  (724) TTTGTGGAAAACAAAATCAAAATTATTCTTCCTCTGACTATGTGTC
      Plant-RNA1-3-rt-2  (724) TTTGTGGAAAACAAAATCAAAATTATTCTTCCTCTGACTATGTGTC
      Plant-RNA1-3'rt-3  (724) TTTGTGGAAAACAAAATCAAAATTATTCTTCCTCTGACTATGTGTC
      Plant-RNA1-3'rt-4  (724) TTTGTGGAAAACAAAATCAAAATTATTCTTCCTCTGACTATGTGTC
     Insect-RNA1-3'rt-1  (724) TTTGTGGAAAACAAAATCAAAATTATTCTTCCTCTGACTATGTGTC
     Insect-RNA1-3'rt-2  (724) TTTGTGGAAAACAAAATCAAAATTATTCTTCCTCTGACTATGTGTC
     Insect-RNA1-3'rt-3  (723) TTTGTG-AAAACAAAATCAAAATTATTCTTCCTCTGACTATGTGTC
     Insect-RNA1-3'rt-4  (724) TTTGTGGAAAACAAAATCAAAATTATTCTTCCTCTGACTATGTGTC
             Consensus (8925) TTTGTGGAAAACAAAATCAAAATTATTCTTCCTCTGACTATGTGTC
                                                                          Section 196
                        (8971) 8971           8985
Newly assembled RSV RNA1 (8971) ATGAACATGCAAGGG
      Plant-RNA1-3'rt-1  (770) ATGAACATGCAAGGG
      Plant-RNA1-3-rt-2  (770) ATGAACATGCAAGGG
      Plant-RNA1-3'rt-3  (770) ATGAACATGCAAGGG
      Plant-RNA1-3'rt-4  (770) ATGAACATGCAAGGG
     Insect-RNA1-3'rt-1  (770) AGCAACATGCAAGGG
     Insect-RNA1-3'rt-2  (770) AGCAACATGCAAGGG
     Insect-RNA1-3'rt-3  (768) ATGAACATGCAAAGG
     Insect-RNA1-3'rt-4  (770) AGCAACATGCAAGGG
             Consensus (8971) ATGAACATGCAAGGG
```

RNA2

```
                                                                  Section 65
                          (2945) 2945 2950      2960      2970      2980      2990
Newly assembled RSV RNA2  (2945) GATTGCAACACCATGATTTATGATTTCTTCTGGTTTCTTAGCTTGG
       Plant-RNA2-3'rt-1     (1) ----------------------------------------------
       Plant-RNA2-3'rt-2     (1) ----------------------------------------------
       Plant-RNA2-3'rt-3     (1) ----------------------------------------------
       Plant-RNA2-3'rt-4     (1) ----------------------------------------------
      Insect-RNA2-3'rt-1     (1) ----------------------------------------------
      Insect-RNA2-3'rt-2     (1) ----------------------------------------------
      Insect-RNA2-3'rt-3     (1) ----------------------------------------------
      Insect-RNA2-3'rt-4     (1) ----------------------------------------------
               Consensus (2945)
                                                                  Section 66
                          (2991) 2991     3000      3010      3020          3036
Newly assembled RSV RNA2  (2991) TCAGGCTGAATGCATGCCCTAGTCTCAACAGCATACACTATTCCCA
       Plant-RNA2-3'rt-1     (1) ------------------CTAGTCTCAACAGCATACACTATTCCCA
       Plant-RNA2-3'rt-2     (1) ------------------CTAGTCTCAACAGCATACACTATTCCCA
       Plant-RNA2-3'rt-3     (1) ------------------CTAGTCTCAACAGCATACACTATTCCCA
       Plant-RNA2-3'rt-4     (1) ------------------CTAGTCTCAACAGCATACACTATTCCCA
      Insect-RNA2-3'rt-1     (1) ------------------CTAGTCTCAACAGCATACACTATTCCCA
      Insect-RNA2-3'rt-2     (1) ------------------CTAGTCTCAACAGCATACACTATTCCCA
      Insect-RNA2-3'rt-3     (1) ------------------CTAGTCTCAACAGCATACACTATTCCCA
      Insect-RNA2-3'rt-4     (1) ------------------CTAGTCTCAACAGCATACACTATTCCCA
               Consensus (2991)                   CTAGTCTCAACAGCATACACTATTCCCA
                                                                  Section 67
                          (3037) 3037         3050      3060      3070      3082
Newly assembled RSV RNA2  (3037) AGAAACCAGTTTTGAGATTTGAGTATGAAATCTTCTCAGAATTGGA
       Plant-RNA2-3'rt-1    (29) AGAAACCAGTTTTGAGATTTGAGTATGAAATCTTCTCAGAATTGGA
       Plant-RNA2-3'rt-2    (29) AGAAACCAGTTTTGAGATTTGAGTATGAAATCTTCTCAGAATTGGA
       Plant-RNA2-3'rt-3    (29) AGAAACCAGTTTTGAGATTTGAGTATGAAATCTTCTCAGAATTGGA
       Plant-RNA2-3'rt-4    (29) AGAAACCAGTTTTGAGATTTGAGTATGAAATCTTCTCAGAATTGGA
      Insect-RNA2-3'rt-1    (29) AGAAACCAGTTTTGAGATTTGAGTATGAAATCTTCTCAGAATTGGA
      Insect-RNA2-3'rt-2    (29) AGAAACCAGTTTTGAGATTTGAGTATGAAATCTTCTCAGAATTGGA
      Insect-RNA2-3'rt-3    (29) AGAAACCAGTTTTGAGATTTGAGTATGAAATCTTCTCAGAATTGGA
      Insect-RNA2-3'rt-4    (29) AGAAACCAGTTTTGAGATTTGAGTATGAAATCTTCTCAGAATTGGA
               Consensus (3037) AGAAACCAGTTTTGAGATTTGAGTATGAAATCTTCTCAGAATTGGA
                                                                  Section 68
                          (3083) 3083   3090      3100      3110            3128
Newly assembled RSV RNA2  (3083) ATTAGTTATCAATAGAGGGCTTGATGTGTATTCTGTTTGCTGTATG
       Plant-RNA2-3'rt-1    (75) ATTAGTTATCAATAGAGGGCTTGATGTGTATTCTGTTTGCTGTATG
       Plant-RNA2-3'rt-2    (75) ATTAGTTATCAATAGAGGGCTTGATGTGTATTCTGTTTGCTGTATG
       Plant-RNA2-3'rt-3    (75) ATTAGTTATCAATAGAGGGCTTGATGTGTATTCTGTTTGCTGTATG
       Plant-RNA2-3'rt-4    (75) ATTAGTTATCAATAGAGGGCTTGATGTGTATTCTGTTTGCTGTATG
      Insect-RNA2-3'rt-1    (75) ATTAGTTATCAATAGAGGGCTTGATGTGTATTCTGTTTGCTGTATG
      Insect-RNA2-3'rt-2    (75) ATTAGTTATCAATAGAGGGCTTGATGTGTATTCTGTTTGCTGTATG
      Insect-RNA2-3'rt-3    (75) ATTAGTTATCAATAGAGGGCTTGATGTGTATTCTGTTTGCTGTATG
      Insect-RNA2-3'rt-4    (75) ATTAGTTATCAATAGAGGGCTTGATGTGTATTCTGTTTGCTGTATG
               Consensus (3083) ATTAGTTATCAATAGAGGGCTTGATGTGTATTCTGTTTGCTGTATG
```

```
                                                                              Section 69
                          (3129) 3129        3140      3150      3160           3174
Newly assembled RSV RNA2 (3129) CCAACCAGAGATACATTCTGCAGAATAGCATCATCGCAACTGAGAT
       Plant-RNA2-3'rt-1  (121) CCAACCAGAGATACATTCTGCAGAATAGCATCATCGCAACTGAGAT
       Plant-RNA2-3'rt-2  (121) CCAACCAGAGATACATTCTGCAGAATAGCATCATCGCAACTGAGAT
       Plant-RNA2-3'rt-3  (121) CCAACCAGAGATACATTCTGCAGAATAGCATCATCGCAACTGAGAT
       Plant-RNA2-3'rt-4  (121) CCAACCAGAGATACATTCTGCAGAATAGCATCATCGCAACTGAGAT
      Insect-RNA2-3'rt-1  (121) CCAACCAGAGATACATTCTGCAGAATAGCATCATCGCAACTGAGAT
      Insect-RNA2-3'rt-2  (121) CCAACCAGAGATACATTCTGCAGAATAGCATCATCGCAACTGAGAT
      Insect-RNA2-3'rt-3  (121) CCAACCAGAGATACATTCTGCAGAATAGCATCATCGCAACTGAGAT
      Insect-RNA2-3'rt-4  (121) CCAACCAGAGATACATTCTGCAGAATAGCATCATCGCAACTGAGAT
               Consensus (3129) CCAACCAGAGATACATTCTGCAGAATAGCATCATCGCAACTGAGAT
                                                                              Section 70
                          (3175) 3175 3180       3190      3200      3210      3220
Newly assembled RSV RNA2 (3175) AGACGTGACCACCGAACTTAGAGAAAGAAGGATGGAGTGGCAGCTT
       Plant-RNA2-3'rt-1  (167) AGACGTGACCACCGAACTTAGAGAAAGAAGGATGGAGTGGCAGCTT
       Plant-RNA2-3'rt-2  (167) AGACGTGACCACCGAACTTAGAGAAAGAAGGATGGAGTGGCAGCTT
       Plant-RNA2-3'rt-3  (167) AGACGTGACCACCGAACTTAGAGAAAGAAGGATGGAGTGGCAGCTT
       Plant-RNA2-3'rt-4  (167) AGACGTGACCACCGAACTTAGAGAAAGAAGGATGGAGTGGCAGCTT
      Insect-RNA2-3'rt-1  (167) AGACGTGACCACCGAACTTAGAGAAAGAAGGATGGAGTGGCAGCTT
      Insect-RNA2-3'rt-2  (167) AGACGTGACCACCGAACTTAGAGAAAGAAGGATGGAGTGGCAGCTT
      Insect-RNA2-3'rt-3  (167) AGACGTGACCACCGAACTTAGAGAAAGAAGGATGGAGTGGCAGCTT
      Insect-RNA2-3'rt-4  (167) AGACGTGACCACCGAACTTAGAGAAAGAAGGATGGAGTGGCAGCTT
               Consensus (3175) AGACGTGACCACCGAACTTAGAGAAAGAAGGATGGAGTGGCAGCTT
                                                                              Section 71
                          (3221) 3221      3230      3240      3250            3266
Newly assembled RSV RNA2 (3221) GTAATCGTAGTTTCCAGTGTAGCATGTCTCAAAGTAATTCACACTA
       Plant-RNA2-3'rt-1  (213) GTAATCGTAGTTTCCAGTGTAGCATGTCTCAAAGTAATTCACACTA
       Plant-RNA2-3'rt-2  (213) GTAATCGTAGTTTCCAGTGTAGCATGTCTCAAAGTAATTCACACTA
       Plant-RNA2-3'rt-3  (213) GTAATCGTAGTTTCCAGTGTAGCATGTCTCAAAGTAATTCACACTA
       Plant-RNA2-3'rt-4  (213) GTAATCGTAGTTTCTTGTGTAGCATGTCTCAAAGTAATTCACACTA
      Insect-RNA2-3'rt-1  (213) GTAATCGTAGTTTCCAGTGTAGCATGTCTCAAAGTAATTCACACTA
      Insect-RNA2-3'rt-2  (213) GTAATCGTAGTTTCCAGTGTAGCATGTCTCAAAGTAATTCACACTA
      Insect-RNA2-3'rt-3  (213) GTAATCGTAGTTTCCAGTGTAGCATGTCTCAAAGTAATTCACACTA
      Insect-RNA2-3'rt-4  (213) GTAATCGTAGTTTCCAGTGTAGCATGTCTCAAAGTAATTCACACTA
               Consensus (3221) GTAATCGTAGTTTCCAGTGTAGCATGTCTCAAAGTAATTCACACTA
                                                                              Section 72
                          (3267) 3267         3280      3290      3300         3312
Newly assembled RSV RNA2 (3267) GCCCTGTTGTAACAAATCAGACCATTTTCAATGAAATATCCATTCA
       Plant-RNA2-3'rt-1  (259) GCCCTGTTGTAACAAATCAGACCATTTTCAATGAAATATCCATTCA
       Plant-RNA2-3'rt-2  (259) GCCCTGTTGTAACAAATCAGACCATTTTCAATGAAATATCCATTCA
       Plant-RNA2-3'rt-3  (259) GCCCTGTTGTAACAAATCAGACCATTTTCAATGAAATATCCATTCA
       Plant-RNA2-3'rt-4  (259) GCCCTGTTGTAACAAATCAGACCATTTTCAATGAAATATCCATTCA
      Insect-RNA2-3'rt-1  (259) GCCCTGTTGTAACAAATCAGACCATTTTCAATGAAATATCCATTCA
      Insect-RNA2-3'rt-2  (259) GCCCTGTTGTAACAAATCAGACCATTTTCAATGAAATATCCATTCA
      Insect-RNA2-3'rt-3  (259) GCCCTGTTGTAACAAATCAGACCATTTTCAATGAAATATCCATTCA
      Insect-RNA2-3'rt-4  (259) GCCCTGTTGTAACAAATCAGACCATTTTCAATGAAATATCCATTCA
               Consensus (3267) GCCCTGTTGTAACAAATCAGACCATTTTCAATGAAATATCCATTCA
```

```
                                                                    Section 73
                      (3313) 3313   3320          3330          3340          3358
Newly assembled RSV RNA2 (3313) GCTCATAAGTCAATTTGCAGGGAGGGGCTCTGGCACTGCAAGGGAC
         Plant-RNA2-3'rt-1  (305) GCTCATAAGTCAATTTGCAGGGAGGGGCTCTGGCACTGCAAGGGAC
         Plant-RNA2-3'rt-2  (305) GCTCATAAGTCAATTTGCAGGGAGGGGCTCTGGCACTGCAAGGGAC
         Plant-RNA2-3'rt-3  (305) GCTCATAAGTCAATTTGCAGGGAGGGGCTCTGGCACTGCAAGGGAC
         Plant-RNA2-3'rt-4  (305) GCTCATAAGTCAATTTGCAGGGAGGGGCTCTGGCACTGCAAGGGAC
        Insect-RNA2-3'rt-1  (305) GCTCATAAGTCAATTTGCAGGGAGGGGCTCTGGCACTGCAAGGGAC
        Insect-RNA2-3'rt-2  (305) GCTCATAAGTCAATTTGCAGGGAGGGGCTCTGGCACTGCAAGGGAC
        Insect-RNA2-3'rt-3  (305) GCTCATAAGTCAATTTGCAGGGAGGGGCTCTGGCACTGCAAGGGAC
        Insect-RNA2-3'rt-4  (305) GCTCATAAGTCAATTTGCAGGGAGGGGCTCTGGCACTGCAAGGGAC
                Consensus (3313) GCTCATAAGTCAATTTGCAGGGAGGGGCTCTGGCACTGCAAGGGAC

                                                                    Section 74
                      (3359) 3359          3370          3380          3390      3404
Newly assembled RSV RNA2 (3359) CTTGACAATCTCAGATGGCTCCCTCTCCCTGTTCCTCATCCAAGAA
         Plant-RNA2-3'rt-1  (351) CTTGACAATCTCAGATGGCTCCCTCTCCCTGTTCCTCATCCAAGAA
         Plant-RNA2-3'rt-2  (351) CTTGACAATCTCAGATGGCTCCCTCTCCCTGTTCCTCATCCAAGAA
         Plant-RNA2-3'rt-3  (351) CTTGACAATCTCAGATGGCTCCCTCTCCCTGTTCCTCATCCAAGAA
         Plant-RNA2-3'rt-4  (351) CTTGACAATCTCAGATGGCTCCCTCTCCCTGTTCCTCATCCAAGAA
        Insect-RNA2-3'rt-1  (351) CTTGACAATCTCAGATGGCTCCCTCTCCCTGTTCCTCATCCAAGAA
        Insect-RNA2-3'rt-2  (351) CTTGACAATCTCAGATGGCTCCCTCTCCCTGTTCCTCATCCAAGAA
        Insect-RNA2-3'rt-3  (351) CTTGACAATCTCAGATGGCTCCCTCTCCCTGTTCCTCATCCAAGAA
        Insect-RNA2-3'rt-4  (351) CTTGACAATCTCAGATGGCTCCCTCTCCCTGTTCCTCATCCAAGAA
                Consensus (3359) CTTGACAATCTCAGATGGCTCCCTCTCCCTGTTCCTCATCCAAGAA

                                                                    Section 75
                      (3405) 3405 3410          3420          3430          3440  3450
Newly assembled RSV RNA2 (3405) TGGGTGTCAGGGAAAGGTATTGGTGCACCCCACGCCATATTAAAGA
         Plant-RNA2-3'rt-1  (397) TGGGTGTCAGGGAAAGGTATTGGTGCACCCCACGCCATATTAAAGA
         Plant-RNA2-3'rt-2  (397) TGGGTGTCAGGGAAAGGTATTGGTGCACCCCACGCCATATTAAAGA
         Plant-RNA2-3'rt-3  (397) TGGGTGTCAGGGAAAGGTATTGGTGCACCCCACGCCATATTAAAGA
         Plant-RNA2-3'rt-4  (397) TGGGTGTCAGGGAAAGGTATTGGTGCACCCCACGCCATATTAAAGA
        Insect-RNA2-3'rt-1  (397) TGGGTGTCAGGGAAAGGTATTGGTGCACCCCACGCCATATTAAAGA
        Insect-RNA2-3'rt-2  (397) TGGGTGTCAGGGAAAGGTATTGGTGCACCCCACGCCATATTAAAGA
        Insect-RNA2-3'rt-3  (397) TGGGTGTCAGGGAAAGGTATTGGTGCACCCCACGCCATATTAAAGA
        Insect-RNA2-3'rt-4  (397) TGGGTGTCAGGGAAAGGTATTGGTGCACCCCACGCCATATTAAAGA
                Consensus (3405) TGGGTGTCAGGGAAAGGTATTGGTGCACCCCACGCCATATTAAAGA

                                                                    Section 76
                      (3451) 3451     3460          3470          3480          3496
Newly assembled RSV RNA2 (3451) TTGTGGTGTAGATGAAATATGATTTAAAATGCATCTTCGAAGAAGT
         Plant-RNA2-3'rt-1  (443) TTGTGGTGTAGATGAAATATGATTTAAAATGCATCTTCGAAGAAGT
         Plant-RNA2-3'rt-2  (443) TTGTGGTGTAGATGAAATATGATTTAAAATGCATCTTCGAAGAAGT
         Plant-RNA2-3'rt-3  (443) TTGTGGTGTAGATGAAATATGATTTAAAATGCATCTTCGAAGAAGT
         Plant-RNA2-3'rt-4  (443) TTGTGGTGTAGATGAAATATGATTTAAAATGCATCTTCGAAGAAGT
        Insect-RNA2-3'rt-1  (443) TTGTGGTGTAGATGAAATATGATTTAAAATGCATCTTCGAAGAAGT
        Insect-RNA2-3'rt-2  (443) TTGTGGTGTAGATGAAATATGATTTAAAATGCATCTTCGAAGAAGT
        Insect-RNA2-3'rt-3  (443) TTGTGGTGTAGATGAAATATGATTTAAAATGCATCTTCGAAGAAGT
        Insect-RNA2-3'rt-4  (443) TTGTGGTGTAGATGAAATATGATTTAAAATGCATCTTCGAAGAAGT
                Consensus (3451) TTGTGGTGTAGATGAAATATGATTTAAAATGCATCTTCGAAGAAGT

                                                                    Section 77
                      (3497) 3497          3510                3529
Newly assembled RSV RNA2 (3497) TATACCCAGACTTTGTGTAGCATGTCTCAAAGT
         Plant-RNA2-3'rt-1  (489) TATACCCAGACTTTGTGTAGCATGTCTCAAAGT
         Plant-RNA2-3'rt-2  (489) TATACCCAGACTTTGTGTAGCATGTCTCAAAGT
         Plant-RNA2-3'rt-3  (489) TATACCCAGACTTTGTGTAGCATGTCTCAAAGT
         Plant-RNA2-3'rt-4  (489) TATACCCAGACTTTGTGTAGCATGTCTCAAAGT
        Insect-RNA2-3'rt-1  (489) TATACCCAGACTTTGTGTAGCATGTCTCAAAGT
        Insect-RNA2-3'rt-2  (489) TATACCCAGACTTTGTGTAGCATGTCTCAAAGT
        Insect-RNA2-3'rt-3  (489) TATACCCAGACTTTGTGTAGCATGTCTCAAAGT
        Insect-RNA2-3'rt-4  (489) TATACCCAGACTTTGTGTAGCATGTCTCAAAGT
                Consensus (3497) TATACCCAGACTTTGTGTAGCATGTCTCAAAGT
```

RNA3

```
                                                                                 Section 37
                          (1657) 1657         1670      1680      1690          1702
Newly assembled RSV RNA3  (1657) TGCCATCTTCCTTCTTCTTGTCAGCCAGGTTCTTTGACTTGCATGT
        Plant-RNA3-3'rt-1    (1) -----------------------------TTCTTTGACTTGCATGT
        Plant-RNA3-3'rt-2    (1) -----------------------------TTCTTTGACTTGCATGT
        Plant-RNA3-3'rt-3    (1) -----------------------------TTCTTTGACTTGCATGT
        Plant-RNA3-3'rt-4    (1) -----------------------------TTCTTTGACTTGCATGT
       Insect-RNA3-3'rt-1    (1) -----------------------------TTCTTTGACTTGCATGT
       Insect-RNA3-3'rt-2    (1) -----------------------------TTCTTTGACTTGCATGT
       Insect-RNA3-3'rt-3    (1) -----------------------------TTCTTTGACTTGCATGT
       Insect-RNA3-3'rt-4    (1) -----------------------------TTCTTTGACTTGCATGT
                Consensus (1657)                              TTCTTTGACTTGCATGT
                                                                                 Section 38
                          (1703) 1703   1710      1720      1730                1748
Newly assembled RSV RNA3  (1703) GATTACCAGGAGACCAGTTGTGTACTTCATCATGTCTATGTCTTTT
        Plant-RNA3-3'rt-1   (18) GATTACCAGGAGACCAGTTGTGTACTTCATCATGTCTATGTCTTTT
        Plant-RNA3-3'rt-2   (18) GATTACCAGGAGACCAGTTGTGTACTTCATCATGTCTATGTCTTTT
        Plant-RNA3-3'rt-3   (18) GATTACCAGGAGACCAGTTGTGTACTTCATCATGTCTATGTCTTTT
        Plant-RNA3-3'rt-4   (18) GATTACCAGGAGACCAGTTGTGTACTTCATCATGTCTATGTCTTTT
       Insect-RNA3-3'rt-1   (18) GATTACCAGGAGACCAGTTGTGTACTTCATCATGTCTATGTCTTTT
       Insect-RNA3-3'rt-2   (18) GATTACCAGGAGACCAGTTGTGTACTTCATCATGTCTATGTCTTTT
       Insect-RNA3-3'rt-3   (18) GATTACCAGGAGACCAGTTGTGTACTTCATCATGTCTATGTCTTTT
       Insect-RNA3-3'rt-4   (18) GATTACCAGGAGACCAGTTGTGTACTTCATCATGTCTATGTCTTTT
                Consensus (1703) GATTACCAGGAGACCAGTTGTGTACTTCATCATGTCTATGTCTTTT
                                                                                 Section 39
                          (1749) 1749       1760      1770      1780            1794
Newly assembled RSV RNA3  (1749) GTCAGACCACGCTCCTTCTTCTTACCCTCTGGCATGGTCTTTTTGG
        Plant-RNA3-3'rt-1   (64) GTCAGACCACGCTCCTTCTTCTTACCCTCTGGCATGGTCTTTTTGG
        Plant-RNA3-3'rt-2   (64) GTCAGACCACGCTCCTTCTTCTTACCCTCTGGCATGGTCTTTTTGG
        Plant-RNA3-3'rt-3   (64) GTCAGACCACGCTCCTTCTTCTTACCCTCTGGCATGGTCTTTTTGG
        Plant-RNA3-3'rt-4   (64) GTCAGACCACGCTCCTTCTTCTTACCCTCTGGCATGGTCTTTTTGG
       Insect-RNA3-3'rt-1   (64) GTCAGACCACGCTCCTTCTTCTTACCCTCTGGCATGGTCTTTTTGG
       Insect-RNA3-3'rt-2   (64) GTCAGACCACGCTCCTTCTTCTTACCCTCTGGCATGGTCTTTTTGG
       Insect-RNA3-3'rt-3   (64) GTCAGACCACGCTCCTTCTTCTTACCCTCTGGCATGGTCTTTTTGG
       Insect-RNA3-3'rt-4   (64) GTCAGACCACGCTCCTTCTTCTTACCCTCTGGCATGGTCTTTTTGG
                Consensus (1749) GTCAGACCACGCTCCTTCTTCTTACCCTCTGGCATGGTCTTTTTGG
                                                                                 Section 40
                          (1795) 1795 1800       1810      1820      1830       1840
Newly assembled RSV RNA3  (1795) TTATTTGGAAGGAGTGTATGATGTGCACAACAAACAAAATGTGACT
        Plant-RNA3-3'rt-1  (110) TTATTTGGAAGGAGTGTATGATGTGCACAACAAACAAAATGTGACT
        Plant-RNA3-3'rt-2  (110) TTATTTGGAAGGAGTGTATGATGTGCACAACAAACAAAATGTGACT
        Plant-RNA3-3'rt-3  (110) TTATTTGGAAGGAGTGTATGATGTGCACAACAAACAAAATGTGACT
        Plant-RNA3-3'rt-4  (110) TTATTTGGAAGGAGTGTATGATGTGCACAACAAACAAAATGTGACT
       Insect-RNA3-3'rt-1  (110) TTATTTGGAAGGAGTGTATGATGTGCACAACAAACAAAATGTGACT
       Insect-RNA3-3'rt-2  (110) TTATTTGGAAGGAGTGTATGATGTGCACAACAAACAAAATGTGACT
       Insect-RNA3-3'rt-3  (110) TTATTTGGAAGGAGTGTATGATGTGCACAACAAACAAAATGTGACT
       Insect-RNA3-3'rt-4  (110) TTATTTGGAAGGAGTGTATGATGTGCACAACAAACAAAATGTGACT
                Consensus (1795) TTATTTGGAAGGAGTGTATGATGTGCACAACAAACAAAATGTGACT
```

Section 41

	(1841)	1841 1850 1860 1870 1886
Newly assembled RSV RNA3	(1841)	TAGGGAGTGAGTTGTGCAGTAAGGAGCTGTTTCACTCTCCAGTTTG
Plant-RNA3-3'rt-1	(156)	TAGGGAGTGAGTTGTGCAGTAAGGAGCTGTTTCACTCTCCAGTTTG
Plant-RNA3-3'rt-2	(156)	TAGGGAGTGAGTTGTGCAGTAAGGAGCTGTTTCACTCTCCAGTTTG
Plant-RNA3-3'rt-3	(156)	TAGGGAGTGAGTTGTGCAGTAAGGAGCTGTTTCACTCTCCAGTTTG
Plant-RNA3-3'rt-4	(156)	TAGGGAGTGAGTTGTGCAGTAAGGAGCTGTTTCACTCTCCAGTTTG
Insect-RNA3-3'rt-1	(156)	TAGGGAGTGAGTTGTGCAGTAAGGAGCTGTTTCACTCTCCAGTTTG
Insect-RNA3-3'rt-2	(156)	TAGGGAGTGAGTTGTGCAGTAAGGAGCTGTTTCACTCTCCAGTTTG
Insect-RNA3-3'rt-3	(156)	TAGGGAGTGAGTTGTGCAGTAAGGAGCTGTTTCACTCTCCAGTTTG
Insect-RNA3-3'rt-4	(156)	TAGGGAGTGAGTTGTGCAGTAAGGAGCTGTTTCACTCTCCAGTTTG
Consensus	(1841)	TAGGGAGTGAGTTGTGCAGTAAGGAGCTGTTTCACTCTCCAGTTTG

Section 42

	(1887)	1887 1900 1910 1920 1932
Newly assembled RSV RNA3	(1887)	ATATATTGTGGAACATAGTCCCACAGTAAGTTGTCACATCCTGTTA
Plant-RNA3-3'rt-1	(202)	ATATATTGTGGAACATAGTCCCACAGTAAGTTGTCACATCCTGTTA
Plant-RNA3-3'rt-2	(202)	ATATATTGTGGAACATAGTCCCACAGTAAGTTGTCACATCCTGTTA
Plant-RNA3-3'rt-3	(202)	ATATATTGTGGAACATAGTCCCACAGTAAGTTGTCACATCCTGTTA
Plant-RNA3-3'rt-4	(202)	ATATATTGTGGAACATAGTCCCACAGTAAGTTGTCACATCCTGTTA
Insect-RNA3-3'rt-1	(202)	ATATATTGTGGAACATAGTCCCACAGTAAGTTGTCACATCCTGTTA
Insect-RNA3-3'rt-2	(202)	ATATATTGTGGAACATAGTCCCACAGTAAGTTGTCACATCCTGTTA
Insect-RNA3-3'rt-3	(202)	ATATATTGTGGAACATAGTCCCACAGTAAGTTGTCACATCCTGTTA
Insect-RNA3-3'rt-4	(202)	ATATATTGTGGAACATAGTCCCACAGTAAGTTGTCACATCCTGTTA
Consensus	(1887)	ATATATTGTGGAACATAGTCCCACAGTAAGTTGTCACATCCTGTTA

Section 43

	(1933)	1933 1940 1950 1960 1978
Newly assembled RSV RNA3	(1933)	GTCCCAGGTCAGAAGAGTCCACAGCCATCTTAACACCAGAAATTTG
Plant-RNA3-3'rt-1	(248)	GTCCCAGGTCAGAAGAGTCCACAGCCATCTTAACACCAGAAATTTG
Plant-RNA3-3'rt-2	(248)	GTCCCAGGTCAGAAGAGTCCACAGCCATCTTAACACCAGAAATTTG
Plant-RNA3-3'rt-3	(248)	GTCCCAGGTCAGAAGAGTCCACAGCCATCTTAACACCAGAAATTTG
Plant-RNA3-3'rt-4	(248)	GTCCCAGGTCAGAAGAGTCCACAGCCATCTTAACACCAGAAATTTG
Insect-RNA3-3'rt-1	(248)	GTCCCAGGTCAGAAGAGTCCACAGCCATCTTAACACCAGAAATTTG
Insect-RNA3-3'rt-2	(248)	GTCCCAGGTCAGAAGAGTCCACAGCCATCTTAACACCAGAAATTTG
Insect-RNA3-3'rt-3	(248)	GTCCCAGGTCAGAAGAGTCCACAGCCATCTTAACACCAGAAATTTG
Insect-RNA3-3'rt-4	(248)	GTCCCAGGTCAGAAGAGTCCACAGCCATCTTAACACCAGAAATTTG
Consensus	(1933)	GTCCCAGGTCAGAAGAGTCCACAGCCATCTTAACACCAGAAATTTG

Section 44

	(1979)	1979 1990 2000 2010 2024
Newly assembled RSV RNA3	(1979)	TCTCACAACTTCATGTGAGACATTTGGGAATAGCTGAGCCAGCCTT
Plant-RNA3-3'rt-1	(294)	TCTCACAACTTCATGTGAGACATTTGGGAATAGCTGAGCCAGCCTT
Plant-RNA3-3'rt-2	(294)	TCTCACAACTTCATGTGAGACATTTGGGAATAGCTGAGCCAGCCTT
Plant-RNA3-3'rt-3	(294)	TCTCACAACTTCATGTGAGACATTTGGGAATAGCTGAGCCAGCCTT
Plant-RNA3-3'rt-4	(294)	TCTCACAACTTCATGTGAGACATTTGGGAATAGCTGAGCCAGCCTT
Insect-RNA3-3'rt-1	(294)	TCTCACAACTTCATGTGAGACATTTGGGAATAGCTGAGCCAGCCTT
Insect-RNA3-3'rt-2	(294)	TCTCACAACTTCATGTGAGACATTTGGGAATAGCTGAGCCAGCCTT
Insect-RNA3-3'rt-3	(294)	TCTCACAACTTCATGTGAGACATTTGGGAATAGCTGAGCCAGCCTT
Insect-RNA3-3'rt-4	(294)	TCTCACAACTTCATGTGAGACATTTGGGAATAGCTGAGCCAGCCTT
Consensus	(1979)	TCTCACAACTTCATGTGAGACATTTGGGAATAGCTGAGCCAGCCTT

```
                                                                      Section 45
                          (2025) 2025 2030      2040      2050      2060      2070
Newly assembled RSV RNA3 (2025) CCAAGTGTGATAGCATTGGCATTTGTTCCCACCGAGGACACTATCC
      Plant-RNA3-3'rt-1   (340) CCAAGTGTGATAGCATTGGCATTTGTTCCCACCGAGGACACTATCC
      Plant-RNA3-3'rt-2   (340) CCAAGTGTGATAGCATTGGCATTTGTTCCCACCGAGGACACTATCC
      Plant-RNA3-3'rt-3   (340) CCAAGTGTGATAGCATTGGCATTTGTTCCCACCGAGGACACTATCC
      Plant-RNA3-3'rt-4   (340) CCAAGTGTGATAGCATTGGCATTTGTTCCCACCGAGGACACTATCC
     Insect-RNA3-3'rt-1   (340) CCAAGTGTGATAGCATTGGCATTTGTTCCCACCGAGGACACTATCC
     Insect-RNA3-3'rt-2   (340) CCAAGTGTGATAGCATTGGCATTTGTTCCCACCGAGGACACTATCC
     Insect-RNA3-3'rt-3   (340) CCAAGTGTGATAGCATTGGCATTTGTTCCCACCGAGGACACTATCC
     Insect-RNA3-3'rt-4   (340) CCAAGTGTGATAGCATTGGCATTTGTTCCCACCGAGGACACTATCC
              Consensus  (2025) CCAAGTGTGATAGCATTGGCATTTGTTCCCACCGAGGACACTATCC
                                                                      Section 46
                          (2071) 2071     2080      2090      2100          2116
Newly assembled RSV RNA3 (2071) CATACCTCGACACCAAGGTCGAAGCCTCTGTGCTTCCAGCCGCCAC
      Plant-RNA3-3'rt-1   (386) CATACCTCGACACCAAGGTCGAAGCCTCTGTGCTTCCAGCCGCCAC
      Plant-RNA3-3'rt-2   (386) CATACCTCGACACCAAGGTCGAAGCCTCTGTGCTTCCAGCCGCCAC
      Plant-RNA3-3'rt-3   (386) CATACCTCGACACCAAGGTCGAAGCCTCTGTGCTTCCAGCCGCCAC
      Plant-RNA3-3'rt-4   (386) CATACCTCGACACCAAGGTCGAAGCCTCTGTGCTTCCAGCCGCCAC
     Insect-RNA3-3'rt-1   (386) CATACCTCGACACCAAGGTCGAAGCCTCTGTGCTTCCAGCCGCCAC
     Insect-RNA3-3'rt-2   (386) CATACCTCGACACCAAGGTCGAAGCCTCTGTGCTTCCAGCCGCCAC
     Insect-RNA3-3'rt-3   (386) CATACCTCGACACCAAGGTCGAAGCCTCTGTGCTTCCAGCCGCCAC
     Insect-RNA3-3'rt-4   (386) CATACCTCGACACCAAGGTCGAAGCCTCTGTGCTTCCAGCCGCCAC
              Consensus  (2071) CATACCTCGACACCAAGGTCGAAGCCTCTGTGCTTCCAGCCGCCAC
                                                                      Section 47
                          (2117) 2117         2130      2140      2150        2162
Newly assembled RSV RNA3 (2117) TTTCACTTTCTTAGTGACATCTCTCACAAAGCCAGTGCCTCTCACA
      Plant-RNA3-3'rt-1   (432) TTTCACTTTCTTAGTGACATCTCTCACAAAGCCAGTGCCTCTCACA
      Plant-RNA3-3'rt-2   (432) TTTCACTTTCTTAGTGACATCTCTCACAAAGCCAGTGCCTCTCACA
      Plant-RNA3-3'rt-3   (432) TTTCACTTTCTTAGTGACATCTCTCACAAAGCCAGTGCCTCTCACA
      Plant-RNA3-3'rt-4   (432) TTTCACTTTCTTAGTGACATCTCTCACAAAGCCAGTGCCTCTCACA
     Insect-RNA3-3'rt-1   (432) TTTCACTTTCTTAGTGACATCTCTCACAAAGCCAGTGCCTCTCACA
     Insect-RNA3-3'rt-2   (432) TTTCACTTTCTTAGTGACATCTCTCACAAAGCCAGTGCCTCTCACA
     Insect-RNA3-3'rt-3   (432) TTTCACTTTCTTAGTGACATCTCTCACAAAGCCAGTGCCTCTCACA
     Insect-RNA3-3'rt-4   (432) TTTCACTTTCTTAGTGACATCTCTCACAAAGCCAGTGCCTCTCACA
              Consensus  (2117) TTTCACTTTCTTAGTGACATCTCTCACAAAGCCAGTGCCTCTCACA
                                                                      Section 48
                          (2163) 2163    2170      2180      2190            2208
Newly assembled RSV RNA3 (2163) TATCTCATGGTGATGCACATAGTCATGTCCTTGGCCAGTGTGTCAC
      Plant-RNA3-3'rt-1   (478) TATCTCATGGTGATGCACATAGTCATGTCCTTGGCCAGTGTGTCAC
      Plant-RNA3-3'rt-2   (478) TATCTCATGGTGATGCACATAGTCATGTCCTTGGCCAGTGTGTCAC
      Plant-RNA3-3'rt-3   (478) TATCTCATGGTGATGCACATAGTCATGTCCTTGGCCAGTGTGTCAC
      Plant-RNA3-3'rt-4   (478) TATCTCATGGTGATGCACATAGTCATGTCCTTGGCCAGTGTGTCAC
     Insect-RNA3-3'rt-1   (478) TATCTCATGGTGATGCACATAGTCATATCCTTGGCCAGTGTGTCAC
     Insect-RNA3-3'rt-2   (478) TATCTCATGGTGATGCACATAGTCATGTCCTTGGCCAGTGTGTCAC
     Insect-RNA3-3'rt-3   (478) TATCTCATGGTGATGCACATAGTCATATCCTTGGCCAGTGTGTCAC
     Insect-RNA3-3'rt-4   (478) TATCTCATGGTGATGCACATAGTCATATCCTTGGCCAGTGTGTCAC
              Consensus  (2163) TATCTCATGGTGATGCACATAGTCATGTCCTTGGCCAGTGTGTCAC
```

Section 49

		2209 … 2220 … 2230 … 2240 … 2254
	(2209)	
Newly assembled RSV RNA3	(2209)	CACCTTTGTCCTTCAATATGCCAATCAGAGTTGCAGCATCATAGCC
Plant-RNA3-3'rt-1	(524)	CACCTTTGTCCTTCAATATGCCAATCAGAGTTGCAGCATCATAGCC
Plant-RNA3-3'rt-2	(524)	CACCTTTGTCCTTCAATATGCCAATCAGAGTTGCAGCATCATAGCC
Plant-RNA3-3'rt-3	(524)	CACCTTTGTCCTTCAATATGCCAATCAGAGTTGCAGCATCATAGCC
Plant-RNA3-3'rt-4	(524)	CACCTTTGTCCTTCAATATGCCAATCAGAGTTGCAGCATCATAGCC
Insect-RNA3-3'rt-1	(524)	CACCTTTGTCCTTCAATATGCCAATCAGAGTTGCAGCATCATAGCC
Insect-RNA3-3'rt-2	(524)	CACCTTTGTCCTTCAATATGCCAATCAGAGTTGCAGCATCATAGCC
Insect-RNA3-3'rt-3	(524)	CACCTTTGTCCTTCAATATGCCAATCAGAGTTGCAGCATCATAGCC
Insect-RNA3-3'rt-4	(524)	CACCTTTGTCCTTCAATATGCCAATCAGAGTTGCAGCATCATAGCC
Consensus	(2209)	CACCTTTGTCCTTCAATATGCCAATCAGAGTTGCAGCATCATAGCC

Section 50

		2255 2260 … 2270 … 2280 … 2290 … 2300
	(2255)	
Newly assembled RSV RNA3	(2255)	TGCATACTCTATCTGACCAGCAAAGGTCACAACATCAGCTTTATGA
Plant-RNA3-3'rt-1	(570)	TGCATACTCTATCTGACCAGCAAAGGTCACAACATCAGCTTTATGA
Plant-RNA3-3'rt-2	(570)	TGCATACTCTATCTGACCAGCAAAGGTCACAACATCAGCTTTATGA
Plant-RNA3-3'rt-3	(570)	TGCATACTCTATCTGACCAGCAAAGGTCACAACATCAGCTTTATGA
Plant-RNA3-3'rt-4	(570)	TGCATACTCTATCTGACCAGCAAAGGTCACAACATCAGCTTTATGA
Insect-RNA3-3'rt-1	(570)	TGCATACTCTATCTGACCAGCAAAGGTCACAACATCAGCTTTATGA
Insect-RNA3-3'rt-2	(570)	TGCATACTCTATCTGACCAGCAAAGGTCACAACATCAGCTTTATGA
Insect-RNA3-3'rt-3	(570)	TGCATACTCTATCTGACCAGCAAAGGTCACAACATCAGCTTTATGA
Insect-RNA3-3'rt-4	(570)	TGCATACTCTATCTGACCAGCAAAGGTCACAACATCAGCTTTATGA
Consensus	(2255)	TGCATACTCTATCTGACCAGCAAAGGTCACAACATCAGCTTTATGA

Section 51

		2301 … 2310 … 2320 … 2330 … 2346
	(2301)	
Newly assembled RSV RNA3	(2301)	GCAGTCAGGTAAGACAACGCATCTTTGGAGATGTCATTGATTGCCT
Plant-RNA3-3'rt-1	(616)	GCAGTCAGGTAAGACAACGCATCTTTGGAGATGTCATTGATTGCCT
Plant-RNA3-3'rt-2	(616)	GCAGTCAGGTAAGACAACGCATCTTTGGAGATGTCATTGATTGCCT
Plant-RNA3-3'rt-3	(616)	GCAGTCAGGTAAGACAACGCATCTTTGGAGATGTCATTGATTGCCT
Plant-RNA3-3'rt-4	(616)	GCAGTCAGGTAAGACAACGCATCTTTGGAGATGTCATTGATTGCCT
Insect-RNA3-3'rt-1	(616)	GCAGTCAGGTAAGACAACGCATCTTTGGAGATGTCATTGATTGCCT
Insect-RNA3-3'rt-2	(616)	GCAGTCAGGTAAGACAACGCATCTTTGGAGATGTCATTGATTGCCT
Insect-RNA3-3'rt-3	(616)	GCAGTCAGGTAAGACAACGCATCTTTGGAGATGTCATTGATTGCCT
Insect-RNA3-3'rt-4	(616)	GCAGTCAGGTAAGACAACGCATCTTTGGAGATGTCATTGATTGCCT
Consensus	(2301)	GCAGTCAGGTAAGACAACGCATCTTTGGAGATGTCATTGATTGCCT

Section 52

		2347 … 2360 … 2370 … 2380 … 2392
	(2347)	
Newly assembled RSV RNA3	(2347)	TCTGCAAATCAGCTAGAGTGGCTGGCTTGTTGGTGCCCATTGTAGC
Plant-RNA3-3'rt-1	(662)	TCTGCAAATCAGCTAGAGTGGCTGGCTTGTTGGTGCCCATTGTAGC
Plant-RNA3-3'rt-2	(662)	TCTGCAAATCAGCTAGAGTGGCTGGCTTGTTGGTGCCCATTGTAGC
Plant-RNA3-3'rt-3	(662)	TCTGCAAATCAGCTAGAGTGGCTGGCTTGTTGGTGCCCATTGTAGC
Plant-RNA3-3'rt-4	(662)	TCTGCAAATCAGCTAGAGTGGCTGGCTTGTTGGTGCCCATTGTAGC
Insect-RNA3-3'rt-1	(662)	TCTGCAAATCAGCTAGAGTGGCTGGCTTGTTGGTGCCCATTGTAGC
Insect-RNA3-3'rt-2	(662)	TCTGCAAATCAGCTAGAGTGGCTGGCTTGTTGGTGCCCATTGTAGC
Insect-RNA3-3'rt-3	(662)	TCTGCAAATCAGCTAGAGTGGCTGGCTTGTTGGTGCCCATTGTAGC
Insect-RNA3-3'rt-4	(662)	TCTGCAAATCAGCTAGAGTGGCTGGCTTGTTGGTGCCCATTGTAGC
Consensus	(2347)	TCTGCAAATCAGCTAGAGTGGCTGGCTTGTTGGTGCCCATTGTAGC

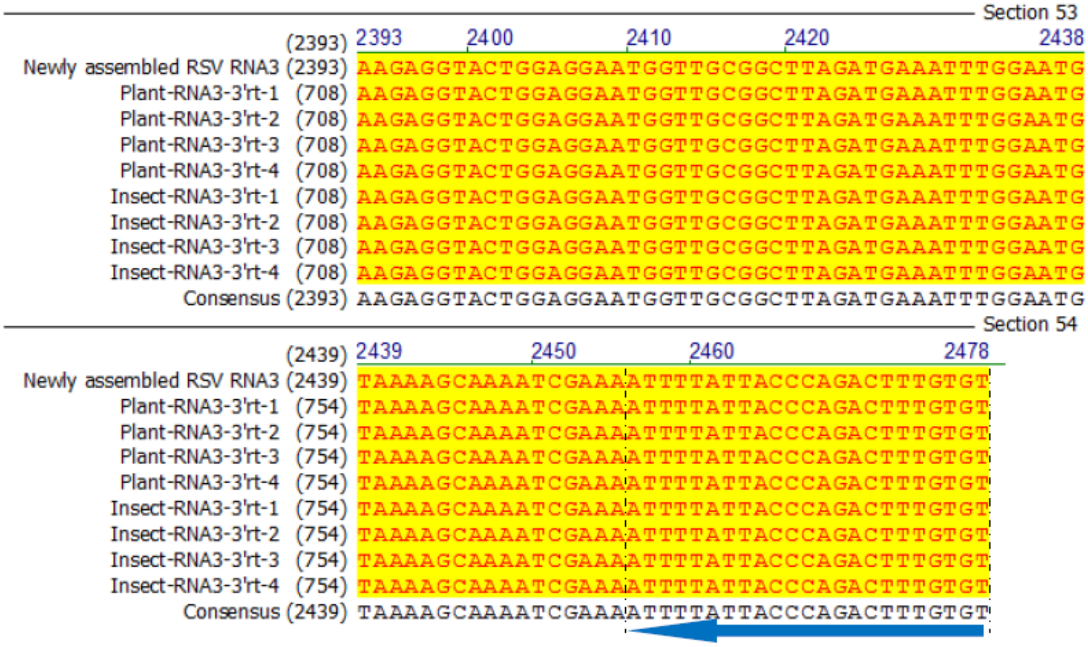
Section 53
(2393) 2393 2400 2410 2420 2438
Newly assembled RSV RNA3 (2393) AAGAGGTACTGGAGGAATGGTTGCGGCTTAGATGAAATTTGGAATG
Plant-RNA3-3'rt-1 (708) AAGAGGTACTGGAGGAATGGTTGCGGCTTAGATGAAATTTGGAATG
Plant-RNA3-3'rt-2 (708) AAGAGGTACTGGAGGAATGGTTGCGGCTTAGATGAAATTTGGAATG
Plant-RNA3-3'rt-3 (708) AAGAGGTACTGGAGGAATGGTTGCGGCTTAGATGAAATTTGGAATG
Plant-RNA3-3'rt-4 (708) AAGAGGTACTGGAGGAATGGTTGCGGCTTAGATGAAATTTGGAATG
Insect-RNA3-3'rt-1 (708) AAGAGGTACTGGAGGAATGGTTGCGGCTTAGATGAAATTTGGAATG
Insect-RNA3-3'rt-2 (708) AAGAGGTACTGGAGGAATGGTTGCGGCTTAGATGAAATTTGGAATG
Insect-RNA3-3'rt-3 (708) AAGAGGTACTGGAGGAATGGTTGCGGCTTAGATGAAATTTGGAATG
Insect-RNA3-3'rt-4 (708) AAGAGGTACTGGAGGAATGGTTGCGGCTTAGATGAAATTTGGAATG
Consensus (2393) AAGAGGTACTGGAGGAATGGTTGCGGCTTAGATGAAATTTGGAATG
Section 54
(2439) 2439 2450 2460 2478
Newly assembled RSV RNA3 (2439) TAAAAGCAAAATCGAAAATTTTATTACCCAGACTTTGTGT
Plant-RNA3-3'rt-1 (754) TAAAAGCAAAATCGAAAATTTTATTACCCAGACTTTGTGT
Plant-RNA3-3'rt-2 (754) TAAAAGCAAAATCGAAAATTTTATTACCCAGACTTTGTGT
Plant-RNA3-3'rt-3 (754) TAAAAGCAAAATCGAAAATTTTATTACCCAGACTTTGTGT
Plant-RNA3-3'rt-4 (754) TAAAAGCAAAATCGAAAATTTTATTACCCAGACTTTGTGT
Insect-RNA3-3'rt-1 (754) TAAAAGCAAAATCGAAAATTTTATTACCCAGACTTTGTGT
Insect-RNA3-3'rt-2 (754) TAAAAGCAAAATCGAAAATTTTATTACCCAGACTTTGTGT
Insect-RNA3-3'rt-3 (754) TAAAAGCAAAATCGAAAATTTTATTACCCAGACTTTGTGT
Insect-RNA3-3'rt-4 (754) TAAAAGCAAAATCGAAAATTTTATTACCCAGACTTTGTGT
Consensus (2439) TAAAAGCAAAATCGAAAATTTTATTACCCAGACTTTGTGT

RNA4

```
                                                                   Section 29
                      (1289) 1289       1300       1310       1320      1334
Newly assembled RSV RNA4 (1289) TTCACCACACCGAACTCCTTCTCAGTGTCCAGCTCAGGGAAGCTCT
        Plant-RNA4-3'rt-1   (1) ----------------------------------------------
        Plant-RNA4-3'rt-2   (1) ----------------------------------------------
        Plant-RNA4-3'rt-3   (1) ----------------------------------------------
        Plant-RNA4-3'rt-4   (1) ----------------------------------------------
       Insect-RNA4-3'rt-1   (1) ----------------------------------------------
       Insect-RNA4-3'rt-2   (1) ----------------------------------------------
       Insect-RNA4-3'rt-3   (1) ----------------------------------------------
       Insect-RNA4-3'rt-4   (1) ----------------------------------------------
           Consensus (1289)

                                                                   Section 30
                      (1335) 1335  1340      1350      1360      1370     1380
Newly assembled RSV RNA4 (1335) TGCTGGTGAAAGCACCTCCAACAGCTTTCTGAACAACATTAGTCGT
        Plant-RNA4-3'rt-1   (1) ----------AGCACCTCCAACAGCTTTCTGAACAACATTAGTCGT
        Plant-RNA4-3'rt-2   (1) ----------AGCACCTCCAACAGCTTTCTGAACAACATTAGTCGT
        Plant-RNA4-3'rt-3   (1) ----------AGCACCTCCAACAGCTTTCTGAACAACATTAGTCGT
        Plant-RNA4-3'rt-4   (1) ----------AGCACCTCCAACAGCTTTCTGAACAACATTAGTCGT
       Insect-RNA4-3'rt-1   (1) ----------AGCACCTCCAACAGCTTTCTGAACAACATTAGTCGT
       Insect-RNA4-3'rt-2   (1) ----------AGCACCTCCAACAGCTTTCTGAACAACATTAGTCGT
       Insect-RNA4-3'rt-3   (1) ----------AGCACCTCCAACAGCTTTCTGAACAACATTAGTCGT
       Insect-RNA4-3'rt-4   (1) ----------AGCACCTCCAACAGCTTTCTGAACAACATTAGTCGT
           Consensus (1335)           AGCACCTCCAACAGCTTTCTGAACAACATTAGTCGT

                                                                   Section 31
                      (1381) 1381     1390      1400      1410            1426
Newly assembled RSV RNA4 (1381) GAAATTACTGAAGCTGGACAAATGGTTTATTGCCTTATCTTCTAGT
        Plant-RNA4-3'rt-1  (37) GAAATTACTGAAGCTGGACAAATGGTTTATTGCCTTATCTTCTAGT
        Plant-RNA4-3'rt-2  (37) AAAATTACTAAAGCTGGACAAATGGTTTATAGCCTTATCTTCTAGT
        Plant-RNA4-3'rt-3  (37) AAAATTACTAAAGCTGGACAAATGGTTTATAGCCTTATCTTCTAGT
        Plant-RNA4-3'rt-4  (37) GAAATTACTGAAGCTGGACAAATGGTTTATTGCCTTATCTTCTAGT
       Insect-RNA4-3'rt-1  (37) GAAATTACTGAAGCTGGACAAATGGTTTATTGCCTTATCTTCTAGT
       Insect-RNA4-3'rt-2  (37) GAAATTACTGAAGCTGGACAAATGGTTTATTGCCTTATCTTCTAGT
       Insect-RNA4-3'rt-3  (37) GAAATTACTGAAGCTGGACAAATGGTTTATTGCCTTATCTTCTAGT
       Insect-RNA4-3'rt-4  (37) GAAATTACTGAAGCTGGACAAATGGTTTATTGCCTTATCTTCTAGT
           Consensus (1381) GAAATTACTGAAGCTGGACAAATGGTTTATTGCCTTATCTTCTAGT

                                                                   Section 32
                      (1427) 1427          1440      1450      1460        1472
Newly assembled RSV RNA4 (1427) GGTTCAAATTCAAACATCACAGTGTCATTGGTCTTCATGGACACTG
        Plant-RNA4-3'rt-1  (83) GGTTCAAATTCAAACATCACAGTGTCATTGGTCTTCATGGACACTG
        Plant-RNA4-3'rt-2  (83) GGTTCAAATTCAAACATCACAGTGTCATTGGTCTTCATGGACACTG
        Plant-RNA4-3'rt-3  (83) GGTTCAAATTCAAACATCACAGTGTCATTGGTCTTCATGGACACTG
        Plant-RNA4-3'rt-4  (83) GGTTCAAATTCAAACATCACAGTGTCATTGGTCTTCATGGACACTG
       Insect-RNA4-3'rt-1  (83) GGTTCAAATTCAAACATCACAGTGTCATTGGTCTTCATGGACACTG
       Insect-RNA4-3'rt-2  (83) GGTTCAAATTCAAACATCACAGTGTCATTGGTCTTCATGGACACTG
       Insect-RNA4-3'rt-3  (83) GGTTCAAATTCAAACATCACAGTGTCATTGGTCTTCATGGACACTG
       Insect-RNA4-3'rt-4  (83) GGTTCAAATTCAAACATCACAGTGTCATTGGTCTTCATGGACACTG
           Consensus (1427) GGTTCAAATTCAAACATCACAGTGTCATTGGTCTTCATGGACACTG
```

```
                                                                  Section 33
                           (1473) 1473  1480         1490          1500                1518
Newly assembled RSV RNA4   (1473) GCAGATCAGTCCTTTCAATCCCCCAGAACCAAAGAGTTCTAGAGAT
Plant-RNA4-3'rt-1          (129)  GCAGATCAGTCCTTTCAATCCCCCAGAACCAAAGAGTTCTAGAGAT
Plant-RNA4-3'rt-2          (129)  GCAGATCAGTCCTCTCAATCCCCCAGAACCAAAGAGTTCTAGAGAT
Plant-RNA4-3'rt-3          (129)  GCAGATCAGTCCTCTCAATCCCCCAGAACCAAAGAGTTCTAGAGAT
Plant-RNA4-3'rt-4          (129)  GCAGATCAGTCCTTTCAATCCCCCAGAACCAAAGAGTTCTAGAGAT
Insect-RNA4-3'rt-1         (129)  GCAGATCAGTCCTTTCAATCCCCCAGAACCAAAGAGTTCTAGAGAT
Insect-RNA4-3'rt-2         (129)  GCAGATCAGTCCTTTCAATCCCCCAGAACCAAAGAGTTCTAGAGAT
Insect-RNA4-3'rt-3         (129)  GCAGATCAGTCCTTTCAATCCCCCAGAACCAAAGAGTTCTAGAGAT
Insect-RNA4-3'rt-4         (129)  GCAGATCAGTCCTTTCAATCCCCCAGAACCAAAGAGTTCTAGAGAT
Consensus                  (1473) GCAGATCAGTCCTTTCAATCCCCCAGAACCAAAGAGTTCTAGAGAT
                                                                  Section 34
                           (1519) 1519         1530       1540       1550             1564
Newly assembled RSV RNA4   (1519) GACACAGTTCTGCACTGAGCTGTCATCAACAGATACGGACACTTGA
Plant-RNA4-3'rt-1          (175)  GACACAGTTCTGCACTGAGCTGTCATCAACAGATACGGACACTTGA
Plant-RNA4-3'rt-2          (175)  GACACAGTTCTGCACTGAGCTGTCATCAACAGATACGGACACTTGA
Plant-RNA4-3'rt-3          (175)  GACACAGTTCTGCACTGAGCTGTCATCAACAGATACGGACACTTGA
Plant-RNA4-3'rt-4          (175)  GACACAGTTCTGCACTGAGCTGTCATCAACAGATACGGACACTTGA
Insect-RNA4-3'rt-1         (175)  GACACAGTTCTGCACTGAGCTGTCATCAACAGATACGGACACTTGA
Insect-RNA4-3'rt-2         (175)  GACACAGTTCTGCACTGAGCTGTCATCAACAGATACGGACACTTGA
Insect-RNA4-3'rt-3         (175)  GACACAGTTCTGCACTGAGCTGTCATCAACAGATACGGACACTTGA
Insect-RNA4-3'rt-4         (175)  GACACAGTTCTGCACTGAGCTGTCATCAACAGATACGGACACTTGA
Consensus                  (1519) GACACAGTTCTGCACTGAGCTGTCATCAACAGATACGGACACTTGA
                                                                  Section 35
                           (1565) 1565  1570       1580       1590       1600     1610
Newly assembled RSV RNA4   (1565) AGATTATGCTTATCCTCCAAAGCCAGAAAATTCGCTAGAGAACCCA
Plant-RNA4-3'rt-1          (221)  AGATTATGCTTATCCTCCAAAGCCAGAAAATTCGCTAGAGAACCCA
Plant-RNA4-3'rt-2          (221)  AGATTATGCTTATCCTCCAAAGCCAGAAAATTCGCCAGAGAACCCA
Plant-RNA4-3'rt-3          (221)  AGATTATGCTTATCCTCCAAAGCCAGAAAATTCGCCAGAGAACCCA
Plant-RNA4-3'rt-4          (221)  AGATTATGCTTATCCTCCAAAGCCAGAAAATTCGCTAGAGAACCCA
Insect-RNA4-3'rt-1         (221)  AGATTATGCTTATCCTCCAAAGCCAGAAAATTCGCTAGAGAACCCA
Insect-RNA4-3'rt-2         (221)  AGATTATGCTTATCCTCCAAAGCCAGAAAATTCGCTAGAGAACCCA
Insect-RNA4-3'rt-3         (221)  AGATTATGCTTATCCTCCAAAGCCAGAAAATTCGCTAGAGAACCCA
Insect-RNA4-3'rt-4         (221)  AGATTATGCTTATCCTCCAAAGCCAGAAAATTCGCTAGAGAACCCA
Consensus                  (1565) AGATTATGCTTATCCTCCAAAGCCAGAAAATTCGCTAGAGAACCCA
                                                                  Section 36
                           (1611) 1611        1620       1630       1640             1656
Newly assembled RSV RNA4   (1611) AGACAGCAAAATTCTTGCTAATTGGGTAGCGAACCTCTACCTCAAC
Plant-RNA4-3'rt-1          (267)  AGACAGCAAAATTCTTGCTAATTGGGTAGCGAACCTCTACCTCAAC
Plant-RNA4-3'rt-2          (267)  AGACAGCAAAATTCTTGCTAATTGGGTAGCGAACCTCTACCTCAAC
Plant-RNA4-3'rt-3          (267)  AGACAGCAAAATTCTTGCTAATTGGGTAGCGAACCTCTACCTCAAC
Plant-RNA4-3'rt-4          (267)  AGACAGCAAAATTCTTGCTAATTGGGTAGCGAACCTCTACCTCAAC
Insect-RNA4-3'rt-1         (267)  AGACAGCAAAATTCTTGCTAATTGGGTAGCGAACCTCTACCTCAAC
Insect-RNA4-3'rt-2         (267)  AGACAGCAAAATTCTTGCTAATTGGGTAGCGAACCTCTACCTCAAC
Insect-RNA4-3'rt-3         (267)  AGACAGCAAAATTCTTGCTAATTGGGTAGCGAACCTCTACCTCAAC
Insect-RNA4-3'rt-4         (267)  AGACAGCAAAATTCTTGCTAATTGGGTAGCGAACCTCTACCTCAAC
Consensus                  (1611) AGACAGCAAAATTCTTGCTAATTGGGTAGCGAACCTCTACCTCAAC
```

Section 37

		1657 1670 1680 1690 1702
	(1657)	
Newly assembled RSV RNA4	(1657)	TTGATCTGAGGAGTTCACATAGGACTTGTCTATTATGCGGAGGGTA
Plant-RNA4-3'rt-1	(313)	TTGATCTGAGGAGTTCACATAGGACTTGTCTATTATGCGGAGGGTA
Plant-RNA4-3'rt-2	(313)	TTGATCTGAGGGGTTCACATAGGACTTGTCTATTATGCGGAGGGTA
Plant-RNA4-3'rt-3	(313)	TTGATCTGAGGGGTTCACATAGGACTTGTCTATTATGCGGAGGGTA
Plant-RNA4-3'rt-4	(313)	TTGATCTGAGGAGTTCACATAGGACTTGTCTATTATGCGGAGGGTA
Insect-RNA4-3'rt-1	(313)	TTGATCTGAGGAGTTCACATAGGACTTGTCTATTATGCGGAGGGTA
Insect-RNA4-3'rt-2	(313)	TTGATCTGAGGAGTTCACATAGGACTTGTCTATTATGCGGAGGGTA
Insect-RNA4-3'rt-3	(313)	TTGATCTGAGGAGTTCACATAGGACTTGTCTATTATGCGGAGGGTA
Insect-RNA4-3'rt-4	(313)	TTGATCTGAGGAGTTCACATAGGACTTGTCTATTATGCGGAGGGTA
Consensus	(1657)	TTGATCTGAGGAGTTCACATAGGACTTGTCTATTATGCGGAGGGTA

Section 38

		1703 1710 1720 1730 1748
	(1703)	
Newly assembled RSV RNA4	(1703)	GTTATTCCACTAGCTCTACCCTTGATTCCAATCCAGACCATAGCAA
Plant-RNA4-3'rt-1	(359)	GTTATTCCACTAGCTCTACCCTTGATTCCAATCCAGACCATAGCAA
Plant-RNA4-3'rt-2	(359)	GTTATTCCACTAGCTCTACCCTTAATTCCAATCCAGACCATAGCAA
Plant-RNA4-3'rt-3	(359)	GTTATTCCACTAGCTCTACCCTTAATTCCAATCCAGACCATAGCAA
Plant-RNA4-3'rt-4	(359)	GTTATTCCACTAGCTCTACCCTTGATTCCAATCCAGACCATAGCAA
Insect-RNA4-3'rt-1	(359)	GTTATTCCACTAGCTCTACCCTTGATTCCAATCCAGACCATAGCAA
Insect-RNA4-3'rt-2	(359)	GTTATTCCACTAGCTCTACCCTTGATTCCAATCCAGACCATAGCAA
Insect-RNA4-3'rt-3	(359)	GTTATTCCACTAGCTCTACCCTTGATTCCAATCCAGACCATAGCAA
Insect-RNA4-3'rt-4	(359)	GTTATTCCACTAGCTCTACCCTTGATTCCAATCCAGACCATAGCAA
Consensus	(1703)	GTTATTCCACTAGCTCTACCCTTGATTCCAATCCAGACCATAGCAA

Section 39

		1749 1760 1770 1780 1794
	(1749)	
Newly assembled RSV RNA4	(1749)	AAGTTGCCACTCTAAAGAATGGATAATGGGTGAGAGGTTGATGAAA
Plant-RNA4-3'rt-1	(405)	AAGTTGCCACTCTAAAGAATGGATAATGGGTGAGAGGTTGATGAAA
Plant-RNA4-3'rt-2	(405)	AAGTTTCCACTCTAAAGAATGGGTAATGGGTGAGAGGTTGATGAAA
Plant-RNA4-3'rt-3	(405)	AAGTTTCCACTCTAAAGAATGGGTAATGGGTGAGAGGTTGATGAAA
Plant-RNA4-3'rt-4	(405)	AAGTTGCCACTCTAAAGAATGGATAATGGGTGAGAGGTTGATGAAA
Insect-RNA4-3'rt-1	(405)	AAGTTGCCACTCTAAAGAATGGATAATGGGTGAGAGGTTGATGAAA
Insect-RNA4-3'rt-2	(405)	AAGTTGCCACTCTAAAGAATGGATAATGGGTGAGAGGTTGATGAAA
Insect-RNA4-3'rt-3	(405)	AAGTTGCCACTCTAAAGAATGGATAATGGGTGAGAGGTTGATGAAA
Insect-RNA4-3'rt-4	(405)	AAGTTGCCACTCTAAAGAATGGATAATGGGTGAGAGGTTGATGAAA
Consensus	(1749)	AAGTTGCCACTCTAAAGAATGGATAATGGGTGAGAGGTTGATGAAA

Section 40

		1795 1800 1810 1820 1830 1840
	(1795)	
Newly assembled RSV RNA4	(1795)	CCAATATGGATCAACAAGTATGTTGAAAGTTGCTCTCCTATGGGCC
Plant-RNA4-3'rt-1	(451)	CCAATATGGATCAACAAGTATGTTGAAAGTTGCTCTCCTATGGGCC
Plant-RNA4-3'rt-2	(451)	CCAATATGGATCAACAAGTATGTTGAAAGTTGCTTTCCTATGGGCC
Plant-RNA4-3'rt-3	(451)	CCAATATGGATCAACAAGTATGTTGAAAGTTGCTTTCCTATGGGCC
Plant-RNA4-3'rt-4	(451)	CCAATATGGATCAACAAGTATGTTGAAAGTTGCTCTCCTATGGGCC
Insect-RNA4-3'rt-1	(451)	CCAATATGGATCAACAAGTATGTTGAAAGTTGCTCTCCTATGGGCC
Insect-RNA4-3'rt-2	(451)	CCAATATGGATCAACAAGTATGTTGAAAGTTGCTCTCCTATGGGCC
Insect-RNA4-3'rt-3	(451)	CCAATATGGATCAACAAGTATGTTGAAAGTTGCTCTCCTATGGGCC
Insect-RNA4-3'rt-4	(451)	CCAATATGGATCAACAAGTATGTTGAAAGTTGCTCTCCTATGGGCC
Consensus	(1795)	CCAATATGGATCAACAAGTATGTTGAAAGTTGCTCTCCTATGGGCC

```
                                                                          Section 41
                           (1841) 1841     1850      1860      1870          1886
Newly assembled RSV RNA4   (1841) TTCACAGAATAGTCCTGTTTGGCTATGAACATATCATACTTGTTCA
       Plant-RNA4-3'rt-1    (497) TTCACAGAATAGTCCTGTTTGGCTATGAACATATCATACTTGTTCA
       Plant-RNA4-3'rt-2    (497) TTCACAGAATAGTCCTGTTTGGCTATGAACATATCATACTTGTTCA
       Plant-RNA4-3'rt-3    (497) TTCACAGAATAGTCCTGTTTGGCTATGAACATATCATACTTGTTCA
       Plant-RNA4-3'rt-4    (497) TTCACAGAATAGTCCTGTTTGGCTATGAACATATCATACTTGTTCA
      Insect-RNA4-3'rt-1    (497) TTCACAGAATAGTCCTGTTTGGCTATGAACATATCATACTTGTTCA
      Insect-RNA4-3'rt-2    (497) TTCACAGAATAGTCCTGTTTGGCTATGAACATATCATACTTGTTCA
      Insect-RNA4-3'rt-3    (497) TTCACAGAATAGTCCTGTTTGGCTATGAACATATCATACTTGTTCA
      Insect-RNA4-3'rt-4    (497) TTCACAGAATAGTCCTGTTTGGCTATGAACATATCATACTTGTTCA
               Consensus   (1841) TTCACAGAATAGTCCTGTTTGGCTATGAACATATCATACTTGTTCA
                                                                          Section 42
                           (1887) 1887        1900      1910      1920       1932
Newly assembled RSV RNA4   (1887) CCTTGACATCTGAGAAGCTGAAGGGTTCCAATCCAAGCATTGTGGC
       Plant-RNA4-3'rt-1    (543) CCTTGACATCTGAGAAGCTGAAGGGTTCCAATCCAAGCATTGTGGC
       Plant-RNA4-3'rt-2    (543) CCTTGACATCTGAGAAGCTGAAGGGTTCCAATCCAAGCATTGTGGC
       Plant-RNA4-3'rt-3    (543) CCTTGACATCTGAGAAGCTGAAGGGTTCCAATCCAAGCATTGTGGC
       Plant-RNA4-3'rt-4    (543) CCTTGACATCTGAGAAGCTGAAGGGTTCCAATCCAAGCATTGTGGC
      Insect-RNA4-3'rt-1    (543) CCTTGACATCTGAGAAGCTGAAGGGTTCCAATCCAAGCATTGTGGC
      Insect-RNA4-3'rt-2    (543) CCTTGACATCTGAGAAGCTGAAGGGTTCCAATCCAAGCATTGTGGC
      Insect-RNA4-3'rt-3    (543) CCTTGACATCTGAGAAGCTGAAGGGTTCCAATCCAAGCATTGTGGC
      Insect-RNA4-3'rt-4    (543) CCTTGACATCTGAGAAGCTGAAGGGTTCCAATCCAAGCATTGTGGC
               Consensus   (1887) CCTTGACATCTGAGAAGCTGAAGGGTTCCAATCCAAGCATTGTGGC
                                                                          Section 43
                           (1933) 1933   1940      1950      1960            1978
Newly assembled RSV RNA4   (1933) AGCTTGGTCAATCGTAACCCTACCCTGGTTCAGAGGCCTCTTGGAG
       Plant-RNA4-3'rt-1    (589) AGCTTGGTCAATCGTAACCCTACCCTGGTTCAGAGGCCTCTTGGAG
       Plant-RNA4-3'rt-2    (589) AGCTTGGTCAATTGTAACCCTACCCTGGTTCAGAGGCCTTTTGGAG
       Plant-RNA4-3'rt-3    (589) AGCTTGGTCAATTGTAACCCTACCCTGGTTCAGAGGCCTTTTGGAG
       Plant-RNA4-3'rt-4    (589) AGCTTGGTCAATTGTAACCCTACCCTGGTTCAGAGGCCTTTTGGAG
      Insect-RNA4-3'rt-1    (589) AGCTTGGTCAATCGTAACCCTACCCTGGTTCAGAGGCCTCTTGGAG
      Insect-RNA4-3'rt-2    (589) AGCTTGGTCAATCGTAACCCTACCCTGGTTCAGAGGCCTCTTGGAG
      Insect-RNA4-3'rt-3    (589) AGCTTGGTCAATCGTAACCCTACCCTGGTTCAGAGGCCTCTTGGAG
      Insect-RNA4-3'rt-4    (589) AGCTTGGTCAATCGTAACCCTACCCTGGTTCAGAGGCCTCTTGGAG
               Consensus   (1933) AGCTTGGTCAATCGTAACCCTACCCTGGTTCAGAGGCCTCTTGGAG
                                                                          Section 44
                           (1979) 1979       1990      2000      2010        2024
Newly assembled RSV RNA4   (1979) AGAGCTAAAGATTTCCTAGTCTTATTATCAACTCTCTTTTGGGACT
       Plant-RNA4-3'rt-1    (635) AGAGCTAAAGATTTCCTAGTCTTATTATCAACTCTCTTTTGGGACT
       Plant-RNA4-3'rt-2    (635) AGAGCTAGAGATTTCCTATTCTTATTATCAACTCTCTTTTGGGACT
       Plant-RNA4-3'rt-3    (635) AGAGCTAGAGATTTCCTATTCTTATTATCAACTCTCTTTTGGGACT
       Plant-RNA4-3'rt-4    (635) AGAGCTAGAGATTTCCTATTCTTATTATCAACTCTCTTTTGGGACT
      Insect-RNA4-3'rt-1    (635) AGAGCTAAAGATTTCCTAGTCTTATTATCAACTCTCTTTTGGGACT
      Insect-RNA4-3'rt-2    (635) AGAGCTAAAGATTTCCTAGTCTTATTATCAACTCTCTTTTGGGACT
      Insect-RNA4-3'rt-3    (635) AGAGCTAAAGATTTCCTAGTCTTATTATCAACTCTCTTTTGGGACT
      Insect-RNA4-3'rt-4    (635) AGAGCTAAAGATTTCCTAGTCTTATTATCAACTCTCTTTTGGGACT
               Consensus   (1979) AGAGCTAAAGATTTCCTAGTCTTATTATCAACTCTCTTTTGGGACT
```

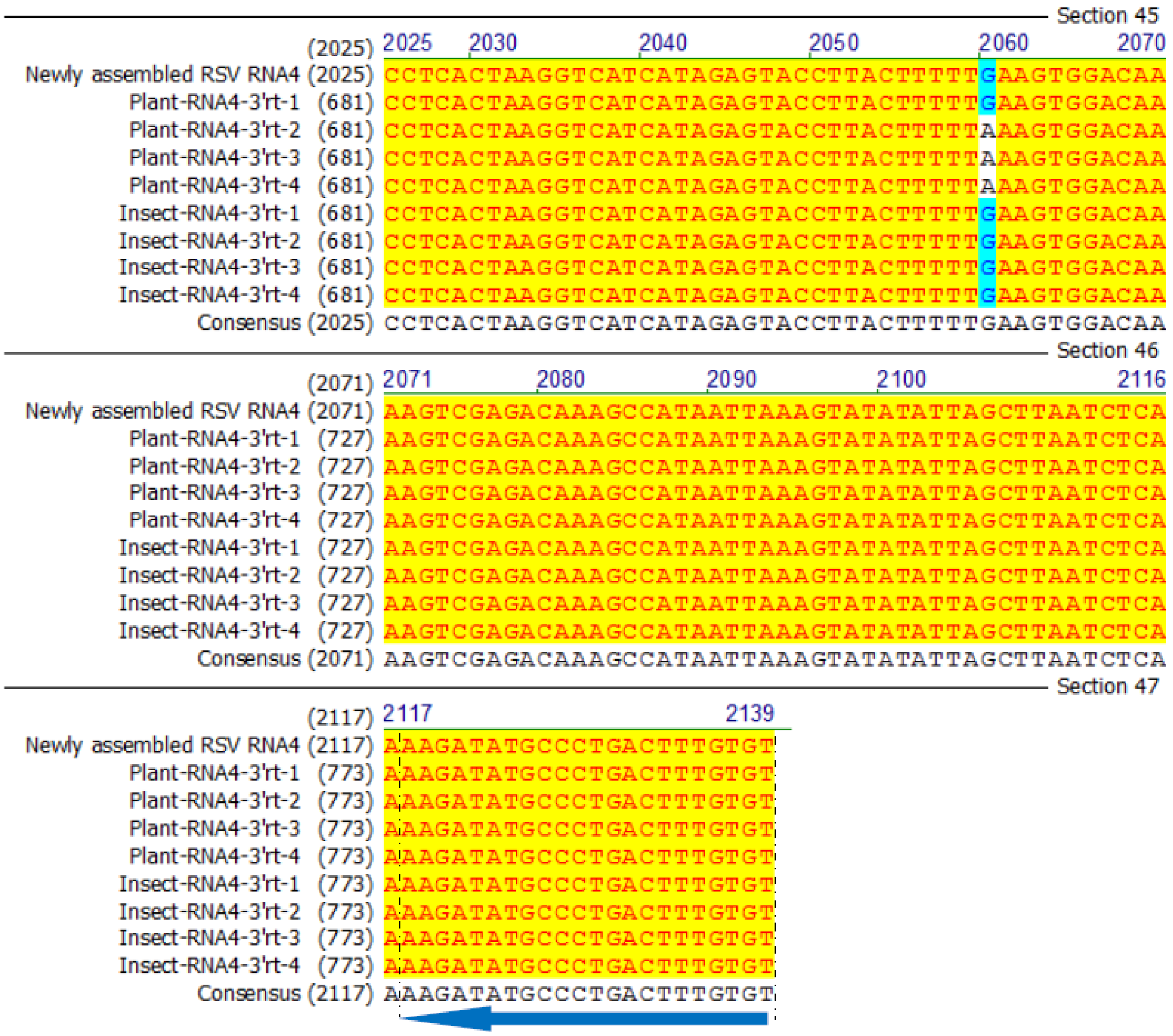
Section 45
(2025) 2025 2030 2040 2050 2060 2070
Newly assembled RSV RNA4 (2025) CCTCACTAAGGTCATCATAGAGTACCTTACTTTTTGAAGTGGACAA
Plant-RNA4-3'rt-1 (681) CCTCACTAAGGTCATCATAGAGTACCTTACTTTTTGAAGTGGACAA
Plant-RNA4-3'rt-2 (681) CCTCACTAAGGTCATCATAGAGTACCTTACTTTTTAAAGTGGACAA
Plant-RNA4-3'rt-3 (681) CCTCACTAAGGTCATCATAGAGTACCTTACTTTTTAAAGTGGACAA
Plant-RNA4-3'rt-4 (681) CCTCACTAAGGTCATCATAGAGTACCTTACTTTTTAAAGTGGACAA
Insect-RNA4-3'rt-1 (681) CCTCACTAAGGTCATCATAGAGTACCTTACTTTTTGAAGTGGACAA
Insect-RNA4-3'rt-2 (681) CCTCACTAAGGTCATCATAGAGTACCTTACTTTTTGAAGTGGACAA
Insect-RNA4-3'rt-3 (681) CCTCACTAAGGTCATCATAGAGTACCTTACTTTTTGAAGTGGACAA
Insect-RNA4-3'rt-4 (681) CCTCACTAAGGTCATCATAGAGTACCTTACTTTTTGAAGTGGACAA
Consensus (2025) CCTCACTAAGGTCATCATAGAGTACCTTACTTTTTGAAGTGGACAA
Section 46
(2071) 2071 2080 2090 2100 2116
Newly assembled RSV RNA4 (2071) AAGTCGAGACAAAGCCATAATTAAAGTATATATTAGCTTAATCTCA
Plant-RNA4-3'rt-1 (727) AAGTCGAGACAAAGCCATAATTAAAGTATATATTAGCTTAATCTCA
Plant-RNA4-3'rt-2 (727) AAGTCGAGACAAAGCCATAATTAAAGTATATATTAGCTTAATCTCA
Plant-RNA4-3'rt-3 (727) AAGTCGAGACAAAGCCATAATTAAAGTATATATTAGCTTAATCTCA
Plant-RNA4-3'rt-4 (727) AAGTCGAGACAAAGCCATAATTAAAGTATATATTAGCTTAATCTCA
Insect-RNA4-3'rt-1 (727) AAGTCGAGACAAAGCCATAATTAAAGTATATATTAGCTTAATCTCA
Insect-RNA4-3'rt-2 (727) AAGTCGAGACAAAGCCATAATTAAAGTATATATTAGCTTAATCTCA
Insect-RNA4-3'rt-3 (727) AAGTCGAGACAAAGCCATAATTAAAGTATATATTAGCTTAATCTCA
Insect-RNA4-3'rt-4 (727) AAGTCGAGACAAAGCCATAATTAAAGTATATATTAGCTTAATCTCA
Consensus (2071) AAGTCGAGACAAAGCCATAATTAAAGTATATATTAGCTTAATCTCA
Section 47
(2117) 2117 2139
Newly assembled RSV RNA4 (2117) AAAGATATGCCCTGACTTTGTGT
Plant-RNA4-3'rt-1 (773) AAAGATATGCCCTGACTTTGTGT
Plant-RNA4-3'rt-2 (773) AAAGATATGCCCTGACTTTGTGT
Plant-RNA4-3'rt-3 (773) AAAGATATGCCCTGACTTTGTGT
Plant-RNA4-3'rt-4 (773) AAAGATATGCCCTGACTTTGTGT
Insect-RNA4-3'rt-1 (773) AAAGATATGCCCTGACTTTGTGT
Insect-RNA4-3'rt-2 (773) AAAGATATGCCCTGACTTTGTGT
Insect-RNA4-3'rt-3 (773) AAAGATATGCCCTGACTTTGTGT
Insect-RNA4-3'rt-4 (773) AAAGATATGCCCTGACTTTGTGT
Consensus (2117) AAAGATATGCCCTGACTTTGTGT

Fig. S2 The 5'-terminal sequences of *Rice stripe virus* (RSV) RNA1 to RNA4 verified using 5'-rapid amplification of cDNA ends experiments and Sanger sequencing. RSV samples residing in rice plants or small brown planthoppers were sequenced. The sequences of four clones are presented for each sample.

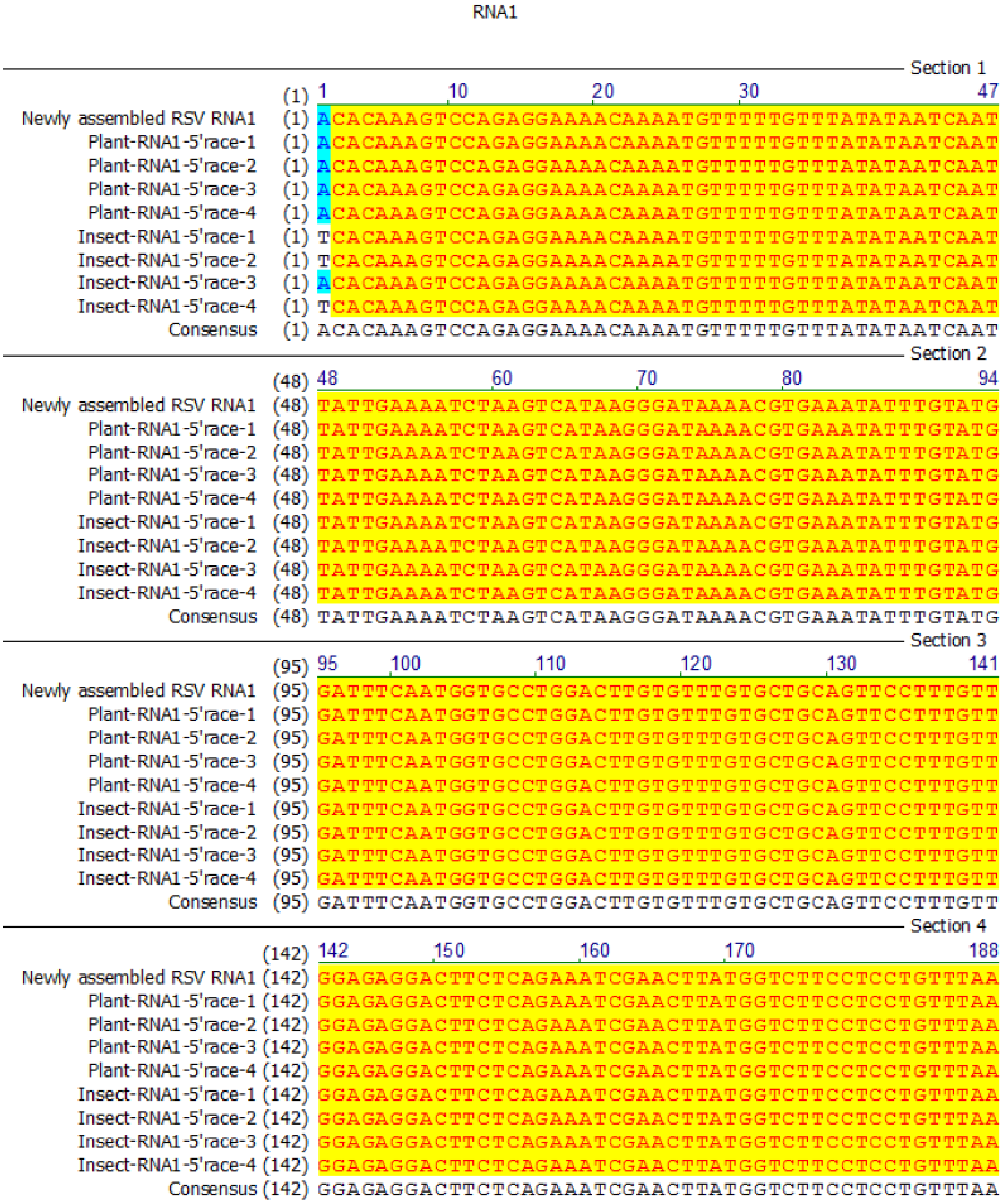

```
                                                                      Section 5
                      (189) 189              200           210           220                235
Newly assembled RSV RNA1 (189) CAAAATAGGCATCATTTATGTCCTGGTACACAAACTTGAACACTTCT
    Plant-RNA1-5'race-1 (189) CAAAATAGGCATCATTTATGTCCTGGTACACAAACTTGAACACTTCT
    Plant-RNA1-5'race-2 (189) CAAAATAGGCATCATTTATGTCCTGGTACACAAACTTGAACACTTCT
    Plant-RNA1-5'race-3 (189) CAAAATAGGCATCATTTATGTCCTGGTACACAAACTTGAACACTTCT
    Plant-RNA1-5'race-4 (189) CAAAATAGGCATCATTTATGTCCTGGTACACAAACTTGAACACTTCT
   Insect-RNA1-5'race-1 (189) CAAAATAGGCATCATTTATGTCCTGGTACACAAACTTGAACACTTCT
   Insect-RNA1-5'race-2 (189) CAAAATAGGCATCATTTATGTCCTGGTACACAAACTTGAACACTTCT
   Insect-RNA1-5'race-3 (189) CAAAATAGGCATCATTTATGTCCTGGTACACAAACTTGAACACTTCT
   Insect-RNA1-5'race-4 (189) CAAAATAGGCATCATTTATGTCCTGGTACACAAACTTGAACACTTCT
              Consensus (189) CAAAATAGGCATCATTTATGTCCTGGTACACAAACTTGAACACTTCT
                                                                      Section 6
                      (236) 236                 250           260           270          282
Newly assembled RSV RNA1 (236) TTCAAGTCTATGATGCTTAGAGACACACTTTTCAAGGGATCTTTCTG
    Plant-RNA1-5'race-1 (236) TTCAAGTCTATGATGCTTAGAGACACACTTTTCAAGGGATCTTTCTG
    Plant-RNA1-5'race-2 (236) TTCAAGTCTATGATGCTTAGAGACACACTTTTCAAGGGATCTTTCTG
    Plant-RNA1-5'race-3 (236) TTCAAGTCTATGATGCTTAGAGACACACTTTTCAAGGGATCTTTCTG
    Plant-RNA1-5'race-4 (236) TTCAAGTCTATGATGCTTAGAGACACACTTTTCAAGGGATCTTTCTG
   Insect-RNA1-5'race-1 (236) TTCAAGTCTATGATGCTTAGAGACACACTTTTCAAGGGATCTTTCTG
   Insect-RNA1-5'race-2 (236) TTCAAGTCTATGATGCTTAGAGACACACTTTTCAAGGGATCTTTCTG
   Insect-RNA1-5'race-3 (236) TTCAAGTCTATGATGCTTAGAGACACACTTTTCAAGGGATCTTTCTG
   Insect-RNA1-5'race-4 (236) TTCAAGTCTATGATGCTTAGAGACACACTTTTCAAGGGATCTTTCTG
              Consensus (236) TTCAAGTCTATGATGCTTAGAGACACACTTTTCAAGGGATCTTTCTG
                                                                      Section 7
                      (283) 283      290           300           310                        329
Newly assembled RSV RNA1 (283) ATTAAAGCTATCAATCAATGATCTGAAGTTGGTCACACCTATCTCTT
    Plant-RNA1-5'race-1 (283) ATTAAAGCTATCAATCAATGATCTGAAGTTGGTCACACCTATCTCTT
    Plant-RNA1-5'race-2 (283) ATTAAAGCTATCAATCAATGATCTGAAGTTGGTCACACCTATCTCTT
    Plant-RNA1-5'race-3 (283) ATTAAAGCTATCAATCAATGATCTGAAGTTGGTCACACCTATCTCTT
    Plant-RNA1-5'race-4 (283) ATTAAAGCTATCAATCAATGATCTGAAGTTGGTCACACCTATCTCTT
   Insect-RNA1-5'race-1 (283) ATTAAAGCTATCAATCAATGATCTGAAGTTGGTCACACCTATCTCTT
   Insect-RNA1-5'race-2 (283) ATTAAAGCTATCAATCAATGATCTGAAGTTGGTCACACCTATCTCTT
   Insect-RNA1-5'race-3 (283) ATTAAAGCTATCAATCAATGATCTGAAGTTGGTCACACCTATCTCTT
   Insect-RNA1-5'race-4 (283) ATTAAAGCTATCAATCAATGATCTGAAGTTGGTCACACCTATCTCTT
              Consensus (283) ATTAAAGCTATCAATCAATGATCTGAAGTTGGTCACACCTATCTCTT
                                                                      Section 8
                      (330) 330           340           350           360                376
Newly assembled RSV RNA1 (330) GAATCATGTACCTAACATAATTTAGTAGGAGAGGGTGGCTCTTGGCT
    Plant-RNA1-5'race-1 (330) GAATCATGTACCTAACATAATTTAGTAGGAGAGGGTGGCTCTTGGCT
    Plant-RNA1-5'race-2 (330) GAATCATGTACCTAACATAATTTAGTAGGAGAGGGTGGCTCTTGGCT
    Plant-RNA1-5'race-3 (330) GAATCATGTACCTAACATAATTTAGTAGGAGAGGGTGGCTCTTGGCT
    Plant-RNA1-5'race-4 (330) GAATCATGTACCTAACATAATTTAGTAGGAGAGGGTGGCTCTTGGCT
   Insect-RNA1-5'race-1 (330) GAATCATGTACCTAACATAATTTAGTAGGAGAGGGTGGCTCTTGGCT
   Insect-RNA1-5'race-2 (330) GAATCATGTACCTAACATAATTTAGTAGGAGAGGGTGGCTCTTGGCT
   Insect-RNA1-5'race-3 (330) GAATCATGTACCTAACATAATTTAGTAGGAGAGGGTGGCTCTTGGCT
   Insect-RNA1-5'race-4 (330) GAATCATGTACCTAACATAATTTAGTAGGAGAGGGTGGCTCTTGGCT
              Consensus (330) GAATCATGTACCTAACATAATTTAGTAGGAGAGGGTGGCTCTTGGCT
```

Section 9

		377 … 390 … 400 … 410 … 423
	(377)	
Newly assembled RSV RNA1	(377)	AAGCTACTCTTGAACACCTCCATTTCTTCAGCCAGGAAGCTCTCTAT
Plant-RNA1-5'race-1	(377)	AAGCTACTCTTGAACACCTCCATTTCTTCAGCCAGGAAGCTCTCTAT
Plant-RNA1-5'race-2	(377)	AAGCTACTCTTGAACACCTCCATTTCTTCAGCCAGGAAGCTCTCTAT
Plant-RNA1-5'race-3	(377)	AAGCTACTCTTGAACACCTCCATTTCTTCAGCCAGGAAGCTCTCTAT
Plant-RNA1-5'race-4	(377)	AAGCTACTCTTGAACACCTCCATTTCTTCAGCCAGGAAGCTCTCTAT
Insect-RNA1-5'race-1	(377)	AAGCTACTCTTGAACACCTCCATTTCTTCAGCCAGGAAGCTCTCTAT
Insect-RNA1-5'race-2	(377)	AAGCTACTCTTGAACACCTCCATTTCTTCAGCCAGGAAGCTCTCTAT
Insect-RNA1-5'race-3	(377)	AAGCTACTCTTGAACACCTCCATTTCTTCAGCCAGGAAGCTCTCTAT
Insect-RNA1-5'race-4	(377)	AAGCTACTCTTGAACACCTCCATTTCTTCAGCCAGGAAGCTCTCTAT
Consensus	(377)	AAGCTACTCTTGAACACCTCCATTTCTTCAGCCAGGAAGCTCTCTAT

Section 10

		424 … 433
	(424)	
Newly assembled RSV RNA1	(424)	AGCAGGGATA
Plant-RNA1-5'race-1	(424)	AGCAGGGATA
Plant-RNA1-5'race-2	(424)	AGCAGGGATA
Plant-RNA1-5'race-3	(424)	AGCAGGGATA
Plant-RNA1-5'race-4	(424)	AGCAGGGATA
Insect-RNA1-5'race-1	(424)	AGCAGGGATA
Insect-RNA1-5'race-2	(424)	AGCAGGGATA
Insect-RNA1-5'race-3	(424)	AGCAGGGATA
Insect-RNA1-5'race-4	(424)	AGCAGGGATA
Consensus	(424)	AGCAGGGATA

RNA2

```
                                                                          Section 1
                          (1) 1         10        20        30               47
Newly assembled RSV RNA2  (1) ACACAAAGTCCTGGGTATATAAGCCACATATATCATATAAATCTAAC
      Plant-RNA2-5'race-1 (1) ACACAAAGTCCTGGGTATATAAGCCACATATATCATATAAATCTAAC
      Plant-RNA2-5'race-2 (1) ACACAAAGTCCTGGGTATATAAGCCACATATATCATATAAATCTAAC
      Plant-RNA2-5'race-3 (1) ACACAAAGTCCTGGGTATATAAGCCACATATATCATATAAATCTAAC
      Plant-RNA2-5'race-4 (1) ACACAAAGTCCTGGGTATATAAGCCACATATATCATATAAATCTAAC
     Insect-RNA2-5'race-1 (1) ACACAAAGTCCTGGGTATATAAGCCACATATATCATATAAATCTAAC
     Insect-RNA2-5'race-2 (1) ACACAAAGTCCTGGGTATATAAGCCACATATATCATATAAATCTAAC
     Insect-RNA2-5'race-3 (1) ACACAAAGTCCTGGGTATATAAGCCACATATATCATATAAATCTAAC
     Insect-RNA2-5'race-4 (1) ACACAAAGTCCTGGGTATATAAGCCACATATATCATATAAATCTAAC
                Consensus (1) ACACAAAGTCCTGGGTATATAAGCCACATATATCATATAAATCTAAC
                                                                          Section 2
                         (48) 48          60        70        80             94
Newly assembled RSV RNA2 (48) AGAAATCGTCCACGGAGAATCTCGTCTTCAGCAATGGCATTACTCCT
      Plant-RNA2-5'race-1 (48) AGAAATCGTCCACGGAGAATCTCGTCTTCAGCAATGGCATTACTCCT
      Plant-RNA2-5'race-2 (48) AGAAATCGTCCACGGAGAATCTCGTCTTCAGCAATGGCATTACTCCT
      Plant-RNA2-5'race-3 (48) AGAAATCGTCCACGGAGAATCTCGTCTTCAGCAATGGCATTACTCCT
      Plant-RNA2-5'race-4 (48) AGAAATCGTCCACGGAGAATCTCGTCTTCAGCAATGGCATTACTCCT
     Insect-RNA2-5'race-1 (48) AGAAATCGTCCACGGAGAATCTCGTCTTCAGCAATGGCATTACTCCT
     Insect-RNA2-5'race-2 (48) AGAAATCGTCCACGGAGAATCTCGTCTTCAGCAATGGCATTACTCCT
     Insect-RNA2-5'race-3 (48) AGAAATCGTCCACGGAGAATCTCGTCTTCAGCAATGGCATTACTCCT
     Insect-RNA2-5'race-4 (48) AGAAATCGTCCACGGAGAATCTCGTCTTCAGCAATGGCATTACTCCT
                Consensus (48) AGAAATCGTCCACGGAGAATCTCGTCTTCAGCAATGGCATTACTCCT
                                                                          Section 3
                         (95) 95  100       110       120       130        141
Newly assembled RSV RNA2 (95) CTTCAATGATCACTACTATGGTTTTCTCCATAAGTATAAAAGACATA
      Plant-RNA2-5'race-1 (95) CTTCAATGATCACTACTATGGTTTTCTCCATAAGTATAAAAGACATA
      Plant-RNA2-5'race-2 (95) CTTCAATGATCACTACTATGGTTTTCTCCATAAGTATAAAAGACATA
      Plant-RNA2-5'race-3 (95) CTTCAATGATCACTACTATGGTTTTCTCCATAAGTATAAAAGACATA
      Plant-RNA2-5'race-4 (95) CTTCAATGATCACTACTATGGTTTTCTCCATAAGTATAAAAGACATA
     Insect-RNA2-5'race-1 (95) CTTCAATGATCACTACTATGGTTTTCTCCATAAGTATAAAAGACATA
     Insect-RNA2-5'race-2 (95) CTTCAATGATCACTACTATGGTTTTCTCCATAAGTATAAAAGACATA
     Insect-RNA2-5'race-3 (95) CTTCAATGATCACTACTATGGTTTTCTCCATAAGTATAAAAGACATA
     Insect-RNA2-5'race-4 (95) CTTCAATGATCACTACTATGGTTTTCTCCATAAGTATAAAAGACATA
                Consensus (95) CTTCAATGATCACTACTATGGTTTTCTCCATAAGTATAAAAGACATA
                                                                          Section 4
                        (142) 142     150       160       170            188
Newly assembled RSV RNA2 (142) CTGGATCATATGATAATCTCTTCAATTTGAGGTGCTCTAAAGAAGAC
      Plant-RNA2-5'race-1 (142) CTGGATCATATGATAATCTCTTCAATTTGAGGTGCTCTAAAGAAGAC
      Plant-RNA2-5'race-2 (142) CTGGATCATATGATAATCTCTTCAATTTGAGGTGCTCTAAAGAAGAC
      Plant-RNA2-5'race-3 (142) CTGGATCATATGATAATCTCTTCAATTTGAGGTGCTCTAAAGAAGAC
      Plant-RNA2-5'race-4 (142) CTGGATCATATGATAATCTCTTCAATTTGAGGTGCTCTAAAGAAGAC
     Insect-RNA2-5'race-1 (142) CTGGATCATATGATAATCTCTTCAATTTGAGGTGCTCTAAAGAAGAC
     Insect-RNA2-5'race-2 (142) CTGGATCATATGATAATCTCTTCAATTTGAGGTGCTCTAAAGAAGAC
     Insect-RNA2-5'race-3 (142) CTGGATCATATGATAATCTCTTCAATTTGAGGTGCTCTAAAGAAGAC
     Insect-RNA2-5'race-4 (142) CTGGATCATATGATAATCTCTTCAATTTGAGGTGCTCTAAAGAAGAC
               Consensus (142) CTGGATCATATGATAATCTCTTCAATTTGAGGTGCTCTAAAGAAGAC
```

```
                                                                    Section 5
                          (189) 189        200       210       220           235
Newly assembled RSV RNA2 (189) CACTATTTGAACTCTCTTGATGCCATCTGGCTCATGGGATGCTGTGA
      Plant-RNA2-5'race-1 (189) CACTATTTGAACTCTCTTGATGCCATCTGGCTCATGGGATGCTGTGA
      Plant-RNA2-5'race-2 (189) CACTATTTGAACTCTCTTGATGCCATCTGGCTCATGGGATGCTGTGA
      Plant-RNA2-5'race-3 (189) CACTATTTGAACTCTCTTGATGCCATCTGGCTCATGGGATGCTGTGA
      Plant-RNA2-5'race-4 (189) CACTATTTGAACTCTCTTGATGCCATCTGGCTCATGGGATGCTGTGA
     Insect-RNA2-5'race-1 (189) CACTATTTGAACTCTCTTGATGCCATCTGGCTCATGGGATGCTGTGA
     Insect-RNA2-5'race-2 (189) CACTATTTGAACTCTCTTGATGCCATCTGGCTCATGGGATGCTGTGA
     Insect-RNA2-5'race-3 (189) CACTATCTGAACTCTCTTGATGCCATCTGGCTCATGGGATGCTGTGA
     Insect-RNA2-5'race-4 (189) CACTATCTGAACTCTCTTGATGCCATCTGGCTCATGGGATGCTGTGA
                Consensus (189) CACTATTTGAACTCTCTTGATGCCATCTGGCTCATGGGATGCTGTGA
                                                                    Section 6
                          (236) 236           250       260       270        282
Newly assembled RSV RNA2 (236) GGAGTTCACAGATCCAGCTCTTAGAGCACATGCTCTCGCCATAGCAA
      Plant-RNA2-5'race-1 (236) GGAGTTCACAGATCCAGCTCTTAGAGCACATGCTCTCGCCATAGCAA
      Plant-RNA2-5'race-2 (236) GGAGTTCACAGATCCAGCTCTTAGAGCACATGCTCTCGCCATAGCAA
      Plant-RNA2-5'race-3 (236) GGAGTTCACAGATCCAGCTCTTAGAGCACATGCTCTCGCCATAGCAA
      Plant-RNA2-5'race-4 (236) GGAGTTCACAGATCCAGCTCTTAGAGCACATGCTCTCGCCATAGCAA
     Insect-RNA2-5'race-1 (236) GGAGTTCACAGATCCAGCTCTTAGAGCACATGCTCTCGCCATAGCAA
     Insect-RNA2-5'race-2 (236) GGAGTTCACAGATCCAGCTCTTAGAGCACATGCTCTCGCCATAGCAA
     Insect-RNA2-5'race-3 (236) GGAGTTCACAGATCCAGCTCTTAGAGCACATGCTCTCGCCATAGCAA
     Insect-RNA2-5'race-4 (236) GGAGTTCACAGATCCAGCTCTTAGAGCACATGCTCTCGCCATAGCAA
                Consensus (236) GGAGTTCACAGATCCAGCTCTTAGAGCACATGCTCTCGCCATAGCAA
                                                                    Section 7
                          (283) 283 285
Newly assembled RSV RNA2 (283) CTG
      Plant-RNA2-5'race-1 (283) CTG
      Plant-RNA2-5'race-2 (283) CTG
      Plant-RNA2-5'race-3 (283) CTG
      Plant-RNA2-5'race-4 (283) CTG
     Insect-RNA2-5'race-1 (283) CTG
     Insect-RNA2-5'race-2 (283) CTG
     Insect-RNA2-5'race-3 (283) CTG
     Insect-RNA2-5'race-4 (283) CTG
                Consensus (283) CTG
```

RNA3

```
                                                                   Section 1
                        (1) 1        10        20        30             47
Newly assembled RSV RNA3 (1) ACACAAAGTCCTGGGTAAAATAGTTATATTTTTACTATACAATACAA
     Plant-RNA3-5'race-1 (1) ACACAAAGTCCTGGGTAAAATAGTTATATTTTTACTATACAATACAA
     Plant-RNA3-5'race-2 (1) ACACAAAGTCCTGGGTAAAATAGTTATATTTTTACTATACAATACAA
     Plant-RNA3-5'race-3 (1) ACACAAAGTCCTGGGTAAAATAGTTATATTTTTACTATACAATACAA
     Plant-RNA3-5'race-4 (1) ACACAAAGTCCTGGGTAAAATAGTTATATTTTTACTATACAATACAA
    Insect-RNA3-5'race-1 (1) ACACAAAGTCCTGGGTAAAATAGTTATATTTTTACTATACAATACAA
    Insect-RNA3-5'race-2 (1) ACACAAAGTCCTGGGTAAAATAGTTATATTTTTACTATACAATACAA
    Insect-RNA3-5'race-3 (1) ACACAAAGTCCTGGGTAAAATAGTTATATTTTTACTATACAATACAA
    Insect-RNA3-5'race-4 (1) ACACAAAGTCCTGGGTAAAATAGTTATATTTTTACTATACAATACAA
               Consensus (1) ACACAAAGTCCTGGGTAAAATAGTTATATTTTTACTATACAATACAA
                                                                   Section 2
                       (48) 48          60        70        80          94
Newly assembled RSV RNA3 (48) TTCCGACATCATCTAAGTATGAACGTGTTCACATCGTCTGTGGGTTC
     Plant-RNA3-5'race-1 (48) TTCCGACATCATCTAAGTATGAACGTGTTCACATCGTCTGTGGGTTC
     Plant-RNA3-5'race-2 (48) TTCCGACATCATCTAAGTATGAACGTGTTCACATCGTCTGTGGGTTC
     Plant-RNA3-5'race-3 (48) TTCCGACATCATCTAAGTATGAACGTGTTCACATCGTCTGTGGGTTC
     Plant-RNA3-5'race-4 (48) TTCCGACATCATCTAAGTATGAACGTGTTCACATCGTCTGTGGGTTC
    Insect-RNA3-5'race-1 (48) TTCCGACATCATCTAAGTATGAACGTATTCACATCGTCTGTGGGTTC
    Insect-RNA3-5'race-2 (48) TTCCGACATCATCTAAGTATGAACGTGTTCACATCGTCTGTGGGTTC
    Insect-RNA3-5'race-3 (48) TTCCGACATCATCTAAGTATGAACGTGTTCACATCGTCTGTGGGTTC
    Insect-RNA3-5'race-4 (48) TTCCGACATCATCTAAGTATGAACGTGTTCACATCGTCTGTGGGTTC
               Consensus (48) TTCCGACATCATCTAAGTATGAACGTGTTCACATCGTCTGTGGGTTC
                                                                   Section 3
                       (95) 95   100       110       120       130       141
Newly assembled RSV RNA3 (95) TGTGGAATTTGATCATCCTCTGCTTTTGGAGAATGATCTGACCAGTT
     Plant-RNA3-5'race-1 (95) TGTGGAATTTGATCATCCTCTGCTTTTGGAGAATGATCTGACCAGTT
     Plant-RNA3-5'race-2 (95) TGTGGAATTTGATCATCCTCTGCTTTTGGAGAATGATCTGACCAGTT
     Plant-RNA3-5'race-3 (95) TGTGGAATTTGATCATCCTCTGCTTTTGGAGAATGATCTGACCAGTT
     Plant-RNA3-5'race-4 (95) TGTGGAATTTGATCATCCTCTGCTTTTGGAGAATGATCTGACCAGTT
    Insect-RNA3-5'race-1 (95) TGTGGAATTTGATCATCCTCTGCTTTTGGAGAATGATCTGACCAGTT
    Insect-RNA3-5'race-2 (95) TGTGGAATTTGATCATCCTCTGCTTTTGGAGAATGATCTGACCAGTT
    Insect-RNA3-5'race-3 (95) TGTGGAATTTGATCATCCTCTGCTTTTGGAGAATGATCTGACCAGTT
    Insect-RNA3-5'race-4 (95) TGTGGAATTTGATCATCCTCTGCTTTTGGAGAATGATCTGACCAGTT
               Consensus (95) TGTGGAATTTGATCATCCTCTGCTTTTGGAGAATGATCTGACCAGTT
                                                                   Section 4
                      (142) 142     150       160       170            188
Newly assembled RSV RNA3 (142) TGAGCATAAACTGTGATGATGTCCATTGCTCTTCAAGAGCCTTATGT
     Plant-RNA3-5'race-1 (142) TGAGCATAAACTGTGATGATGTCCATTGCTCTTCAAGAGCCTTATGT
     Plant-RNA3-5'race-2 (142) TGAGCATAAACTGTGATGATGTCCATTGCTCTTCAAGAGCCTTATGT
     Plant-RNA3-5'race-3 (142) TGAGCATAAACTGTGATGATGTCCATTGCTCTTCAAGAGCCTTATGT
     Plant-RNA3-5'race-4 (142) TGAGCATAAACTGTGATGATGTCCATTGCTCTTCAAGAGCCTTATGT
    Insect-RNA3-5'race-1 (142) TGAGCATAAACTGTGATGATGTCCATTGCTCTTCAAGAGCCTTATGT
    Insect-RNA3-5'race-2 (142) TGAGCATAAACTGTGATGATGTCCATTGCTCTTCAAGAGCCTTATGT
    Insect-RNA3-5'race-3 (142) TGAGCATAAACTGTGATGATGTCCATTGCTCTTCAAGAGCCTTATGT
    Insect-RNA3-5'race-4 (142) TGAGCATAAACTGTGATGATGTCCATTGCTCTTCAAGAGCCTTATGT
               Consensus (142) TGAGCATAAACTGTGATGATGTCCATTGCTCTTCAAGAGCCTTATGT
```

```
                                                                        Section 5
                          (189) 189       200       210       220            235
Newly assembled RSV RNA3 (189) TATATATATGACATTCACTCATCTAGGCACCCTTCCATTGATGAACA
     Plant-RNA3-5'race-1 (189) TATATATATGACATTCACTCATCTAGGCACCCTTCCATTGATGAACA
     Plant-RNA3-5'race-2 (189) TATATATATGACATTCACTCATCTAGGCACCCTTCCATTGATGAACA
     Plant-RNA3-5'race-3 (189) TATATATATGACATTCACTCATCTAGGCACCCTTCCATTGATGAACA
     Plant-RNA3-5'race-4 (189) TATATATATGACATTCACTCATCTAGGCACCCTTCCATTGATGAACA
    Insect-RNA3-5'race-1 (189) TATATATATGACATTCACTCATCTAGGCACCCTTCCATTGATGAACA
    Insect-RNA3-5'race-2 (189) TATATATATGACATTCACTCATCTAGGCACCCTTCCATTGATGAACA
    Insect-RNA3-5'race-3 (189) TATATATATGACATTCACTCATCTAGGCACCCTTCCATTGATGAACA
    Insect-RNA3-5'race-4 (189) TATATATATGACATTCACTCATCTAGGCACCCTTCCATTGATGAACA
               Consensus (189) TATATATATGACATTCACTCATCTAGGCACCCTTCCATTGATGAACA
                                                                        Section 6
                          (236) 236                  257
Newly assembled RSV RNA3 (236) TCAGTTTCTGAGGCTTCTCCAT
     Plant-RNA3-5'race-1 (236) TCAGTTTCTGAGGCTTCTCCAT
     Plant-RNA3-5'race-2 (236) TCAGTTTCTGAGGCTTCTCCAT
     Plant-RNA3-5'race-3 (236) TCAGTTTCTGAGGCTTCTCCAT
     Plant-RNA3-5'race-4 (236) TCAGTTTCTGAGGCTTCTCCAT
    Insect-RNA3-5'race-1 (236) TCAGTTTCTGAGGCTTCTCCAT
    Insect-RNA3-5'race-2 (236) TCAGTTTCTGAGGCTTCTCCAT
    Insect-RNA3-5'race-3 (236) TCAGTTTCTGAGGCTTCTCCAT
    Insect-RNA3-5'race-4 (236) TCAGTTTCTGAGGCTTCTCCAT
               Consensus (236) TCAGTTTCTGAGGCTTCTCCAT
```

RNA4

```
                                                                              Section 1
                            (1) 1        10        20        30              47
Newly assembled RSV RNA4    (1) ACACAAAGTCCAGGGCATTTGTACAACGATCCAGCAATTTAATCAGA
       Plant-RNA4-5'race-1  (1) ACACAAAGTCCAGGGCATTTGTACAACGATCCAGCAATTTAATCAGA
       Plant-RNA4-5'race-2  (1) ACACAAAGTCCAGGGCATTTGTACAACGATCCAGCAATTTAATCAGA
       Plant-RNA4-5'race-3  (1) ACACAAAGTCCAGGGCATTTGTACAACGATCCAGCAATTTAATCAGA
       Plant-RNA4-5'race-4  (1) ACACAAAGTCCAGGGCATTTGTACAACGATCCAGCAATTTAATCAGA
      Insect-RNA4-5'race-1  (1) ACACAAAGTCCAGGGCATTTGTACAACGATCCAGCAATTTAATCAGA
      Insect-RNA4-5'race-2  (1) ACACAAAGTCCAGGGCATTTGTACAACGATCCAGCAATTTAATCAGA
      Insect-RNA4-5'race-3  (1) ACACAAAGTCCAGGGCATTTGTACAACGATCCAGCAATTTAATCAGA
      Insect-RNA4-5'race-4  (1) ACACAAAGTCCAGGGCATTTGTACAACGATCCAGCAATTTAATCAGA
                 Consensus  (1) ACACAAAGTCCAGGGCATTTGTACAACGATCCAGCAATTTAATCAGA
                                                                              Section 2
                           (48) 48          60        70        80           94
Newly assembled RSV RNA4   (48) ATCGAAGATGCAAGACGTACAAAGGACAGTAGAAGTTTCTGTTGGTC
       Plant-RNA4-5'race-1 (48) ATCGAAGATGCAAGACGTACAAAGGACAGTAGAAGTTTCTGTTGGTC
       Plant-RNA4-5'race-2 (48) ATCGAAGATGCAAGACGTACAAAGGACAGTAGAAGTTTCTGTTGGTC
       Plant-RNA4-5'race-3 (48) ATCGAAGATGCAAGACGTACAAAGGACAGTAGAAGTTTCTGTTGGTC
       Plant-RNA4-5'race-4 (48) ATCGAAGATGCAAGACGTACAAAGGACAGTAGAAGTTTCTGTTGGTC
      Insect-RNA4-5'race-1 (48) ATCGAAGATGCAAGACGTACAAAGGACAGTAGAAGTTTCTGTTGGTC
      Insect-RNA4-5'race-2 (48) ATCGAAGATGCAAGACGTACAAAGGACAGTAGAAGTTTCTGTTGGTC
      Insect-RNA4-5'race-3 (48) ATCGAAGATGCAAGACGTACAAAGGACAGTAGAAGTTTCTGTTGGTC
      Insect-RNA4-5'race-4 (48) ATCGAAGATGCAAGACGTACAAAGGACAGTAGAAGTTTCTGTTGGTC
                 Consensus (48) ATCGAAGATGCAAGACGTACAAAGGACAGTAGAAGTTTCTGTTGGTC
                                                                              Section 3
                           (95) 95  100       110       120       130       141
Newly assembled RSV RNA4   (95) CTATTGTAGGCCTAGATTACACTCTATTGTATGACACTCTGCCCGAG
       Plant-RNA4-5'race-1 (95) CTATTGTAGGCCTAGATTACACTCTATTGTATGACACTCTGCCCGAG
       Plant-RNA4-5'race-2 (95) CTATTGTAGGCCTAGATTACACTCTATTGTATGACACTCTGCCCGAG
       Plant-RNA4-5'race-3 (95) CTATTGTAGGCCTAGATTACACTCTATTGTATGACACTCTGCCCGAG
       Plant-RNA4-5'race-4 (95) CTATTGTAGGCCTAGATTACACTCTATTGTATGACACTCTGCCCGAG
      Insect-RNA4-5'race-1 (95) CTATTGTAGGCCTAGATTACACTCTATTGTATGACACTCTGCCCGAG
      Insect-RNA4-5'race-2 (95) CTATTGTAGGCCTAGATTACACTCTATTGTATGACACTCTGCCCGAG
      Insect-RNA4-5'race-3 (95) CTATTGTAGGCCTAGATTACACTCTATTGTATGACACTCTGCCCGAG
      Insect-RNA4-5'race-4 (95) CTATTGTAGGCCTAGATTACACTCTATTGTATGACACTCTGCCCGAG
                 Consensus (95) CTATTGTAGGCCTAGATTACACTCTATTGTATGACACTCTGCCCGAG
                                                                              Section 4
                          (142) 142     150       160       170             188
Newly assembled RSV RNA4  (142) ACTGTTAGCGATAACATTACTCTACCTGATTTGAAAGATCCAGAGAG
       Plant-RNA4-5'race-1 (142) ACTGTTAGCGATAACATTACTCTACCTGATTTGAAAGATCCAGAGAG
       Plant-RNA4-5'race-2 (142) ACTGTTAGCGATAACATTACTCTACCTGATTTGAAAGATCCAGAGAG
       Plant-RNA4-5'race-3 (142) ACTGTTAGCGATAACATTACTCTACCTGATTTGAAAGATCCAGAGAG
       Plant-RNA4-5'race-4 (142) ACTGTTAGCGATAACATTACTCTACCTGATTTGAAAGATCCAGAGAG
      Insect-RNA4-5'race-1 (142) ACTGTTAGCGATAACATTACTCTACCTGATTTGAAAGATCCAGAGAG
      Insect-RNA4-5'race-2 (142) ACTGTTAGCGATAACATTACTCTACCTGATTTGAAAGATCCAGAGAG
      Insect-RNA4-5'race-3 (142) ACTGTTAGCGATAACATTACTCTACCTGATTTGAAAGATCCAGAGAG
      Insect-RNA4-5'race-4 (142) ACTGTTAGCGATAACATTACTCTACCTGATTTGAAAGATCCAGAGAG
                 Consensus (142) ACTGTTAGCGATAACATTACTCTACCTGATTTGAAAGATCCAGAGAG

                                                                              Section 5
                          (189) 189           203
Newly assembled RSV RNA4  (189) AGTCACGGAAGATAC
       Plant-RNA4-5'race-1 (189) AGTCACGGAAGATAC
       Plant-RNA4-5'race-2 (189) AGTCACGGAAGATAC
       Plant-RNA4-5'race-3 (189) AGTCACGGAAGATAC
       Plant-RNA4-5'race-4 (189) AGTCACGGAAGATAC
```

Fig. S3 Phylogenetic analysis of the *Rice stripe virus* (RSV) isolates based on the *CP* gene. The *CP* sequences from the five isolates of this study (marked with triangles) and from another seventy-five RSV isolates reported by other laboratories (Kakutani *et al.*, 1991) (Zhu *et al.*, 1991; Qu *et al.*, 1997; Wei *et al.*, 2009) were included for the phylogenetic analysis. The RNA3 of *Maize stripe virus* (S40180 in GenBank) was used as the outgroup. The tree was constructed using the Neighbour-Joining algorithm with the Kimura two-parameter model implemented in MEGA 5.0. Branches with ＜50% bootstrap values were collapsed. The bootstrap values of more than 80% are marked in red, and the values in the range from 50% to 80% are marked in dark red. The populations from Eastern China (shaded in pink) belong to subtype I, and the populations from Southwest China (shaded in blue) mostly belong to subtype II (Wei *et al.*, 2009).

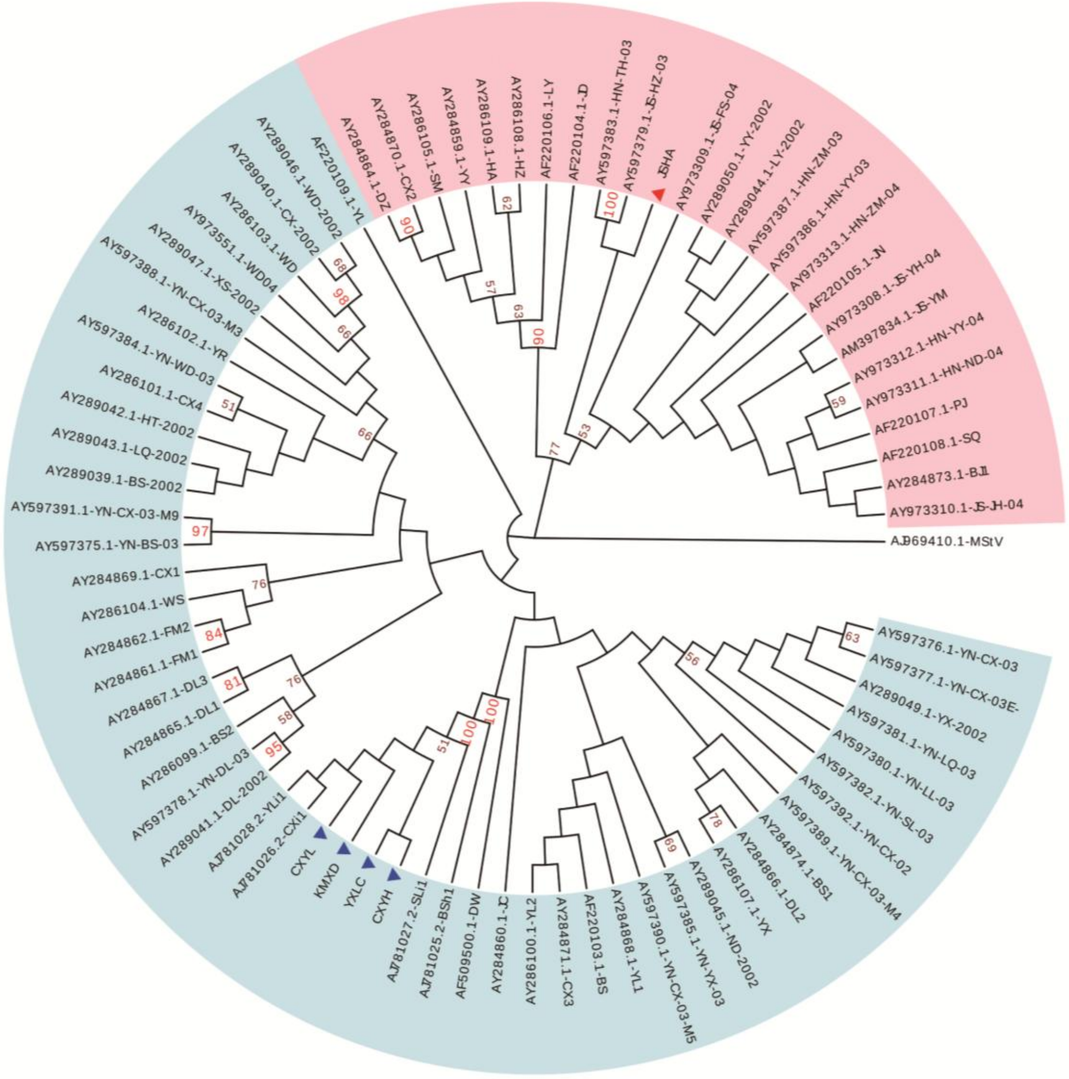

Fig. S4 Comparison of the 3'-terminal 300 nucleotides of RNA segments 4 through 8 of *Southern rice black-streaked dwarf virus* (SRBSDV) between the published reference genome and our *de novo* assembled genome. The SRBSDV genome sequencing data were retrieved from the work of Wang *et al*. 2016 (Wang, L *et al.*, 2016a). Two versions of the genome were assembled with SRBSDV reads from the viruliferous strains with high viral titres (HVT) and with medium viral titres (MVT). The *de novo* assembled RNA segments 4 through 8 contain 3' terminal extension sequences of 13-nt to 17-nt. Each extension sequence is identical or nearly identical to a fragment in the upstream region of the same RNA segment, which is highlighted with a red box.

RNA4

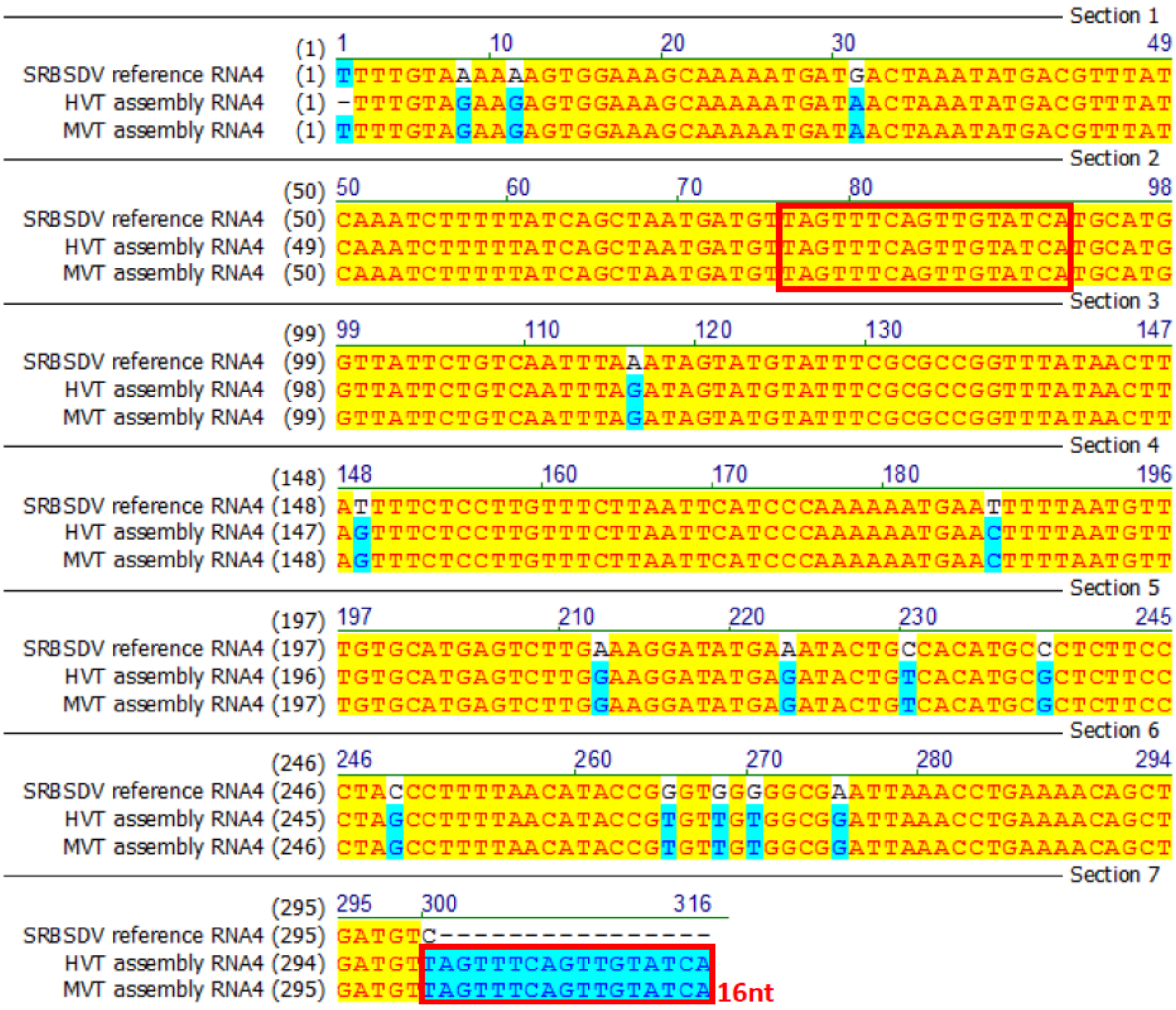

RNA5

```
                                                                          Section 1
                          (1) 1       10        20        30              49
SRBSDV reference RNA5     (1) -TACTTTACACATATTAAACCGTATTTTAAATGTTTCTAAAGACGTAAT
HVT assembly RNA5         (1) CTGCTTTACACATATTAAACCGCATTTTAAATGTTTCTAAAGACGTAAT
MVT assembly RNA5         (1) CTGCTTTACACATATTAAACCGCATTTTAAATGTTTCTAAAGACGTAAT
                                                                          Section 2
                         (50) 50        60        70        80              98
SRBSDV reference RNA5    (49) TCCATTTTTATTTGATCACAATCACAACGCTAACAATAAAACGATGTCT
HVT assembly RNA5        (50) TCCATTTTTATTTGATCACAATCACAACGCTAACAATAAAACGCTGTTT
MVT assembly RNA5        (50) TCCATTTTTATTTGATCACAATCACAACGCTAACAATAAAACGCTGTTT
                                                                          Section 3
                         (99) 99         110       120       130            147
SRBSDV reference RNA5    (98) GACTTATCTTCTACGCTTAACAATTGTCGTGATATGAAAGTTTCTGAGA
HVT assembly RNA5        (99) GACTTATCTTCTACGCTTAACAATTGTCGTGATATGAAAGTTTCTGAGA
MVT assembly RNA5        (99) GACTTATCTTCTACGCTTAACAATTGTCGTGATATGAAAGTTTCTGAGA
                                                                          Section 4
                        (148) 148         160       170       180           196
SRBSDV reference RNA5   (147) GACATCACTTACTTGGAACTTTACTTTTCGGAAGCACGCTAGGAGTTAT
HVT assembly RNA5       (148) GACATAACTTACTTAGAACTTTACTTTTCGGAAGCACGCTAGGAGTTAT
MVT assembly RNA5       (148) GACATAACTTACTTAGAACTTTACTTTTCGGAAGCACGCTAGGAGTTAT
                                                                          Section 5
                        (197) 197          210       220       230          245
SRBSDV reference RNA5   (196) ACTTCATGTTTGAGGGTGTGTTATCCCTGGCTGCAAGCGGGAGGCGCGT
HVT assembly RNA5       (197) ACTTCATGTTTGAGGGTGTGTTATCCCTGGCTGCAAGCGGGAGGCGCGT
MVT assembly RNA5       (197) ACTTCATGTTTGAGGGTGTGTTATCCCTGGCTGCAAGCGGGAGGCGCGT
                                                                          Section 6
                        (246) 246           260       270       280         294
SRBSDV reference RNA5   (245) GAACAGGTGTTCCACGTCTTCTTAGATGCGGGGGGAGTGAATACAGCT
HVT assembly RNA5       (246) GAACAGGTGTTCCACGTCTTCTTAGATGCGGGGGGAGTGAATACAGCT
MVT assembly RNA5       (246) GAACAGGTGTTCCACGTCTTCTTAGATGCGGGGGGAGTGAATACAGCT
                                                                          Section 7
                        (295) 295  300              317
SRBSDV reference RNA5   (294) GATGTC-----------------
HVT assembly RNA5       (295) GATGTCTGACTTATCTTCTACGC 17nt
MVT assembly RNA5       (295) GAT--------------------
```

RNA6

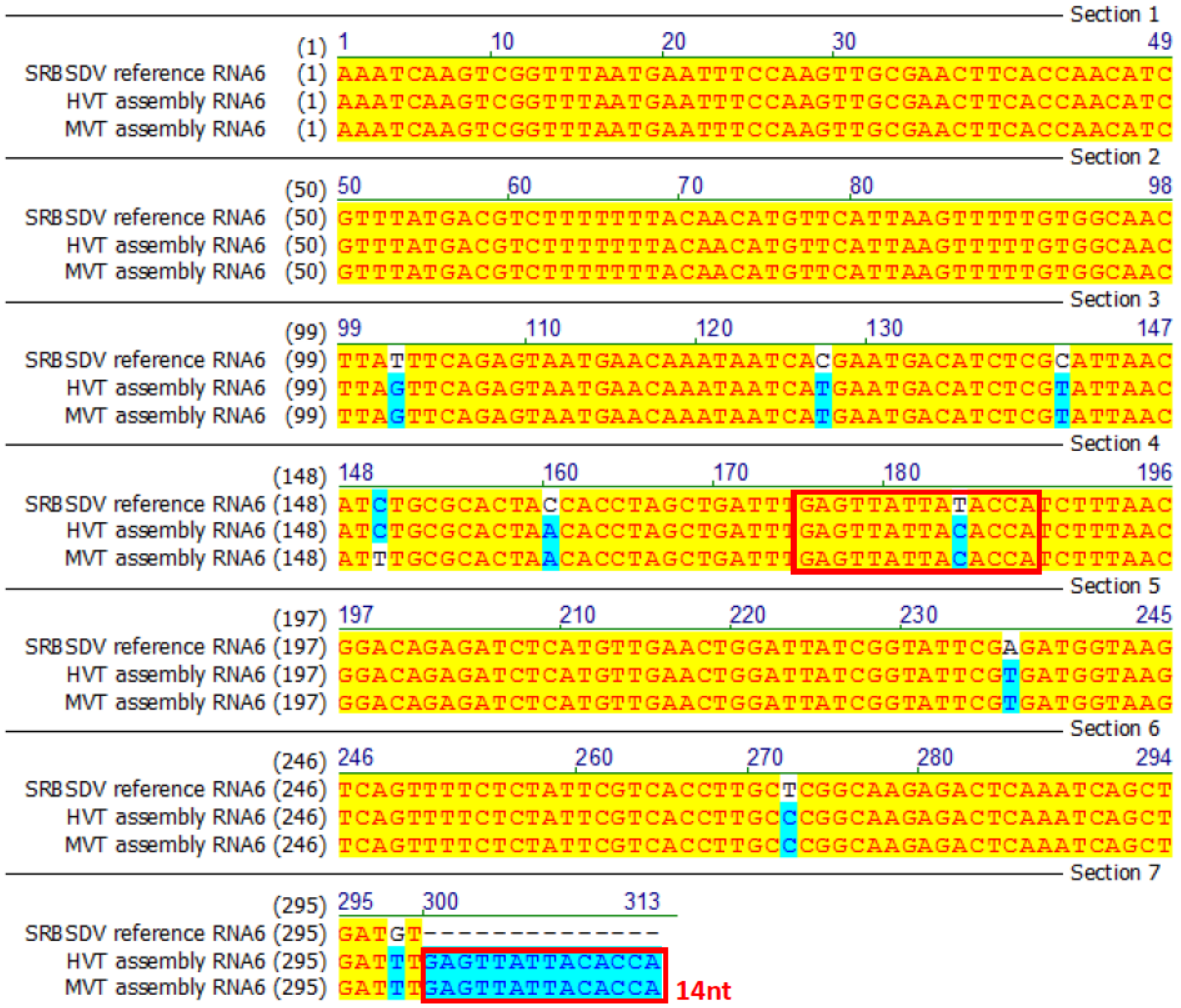

Section 1
(1) 1 10 20 30 49
SRBSDV reference RNA6 (1) AAATCAAGTCGGTTTAATGAATTTCCAAGTTGCGAACTTCACCAACATC
HVT assembly RNA6 (1) AAATCAAGTCGGTTTAATGAATTTCCAAGTTGCGAACTTCACCAACATC
MVT assembly RNA6 (1) AAATCAAGTCGGTTTAATGAATTTCCAAGTTGCGAACTTCACCAACATC
Section 2
(50) 50 60 70 80 98
SRBSDV reference RNA6 (50) GTTTATGACGTCTTTTTTTACAACATGTTCATTAAGTTTTTGTGGCAAC
HVT assembly RNA6 (50) GTTTATGACGTCTTTTTTTACAACATGTTCATTAAGTTTTTGTGGCAAC
MVT assembly RNA6 (50) GTTTATGACGTCTTTTTTTACAACATGTTCATTAAGTTTTTGTGGCAAC
Section 3
(99) 99 110 120 130 147
SRBSDV reference RNA6 (99) TTATTTCAGAGTAATGAACAAATAATCACGAATGACATCTCGCATTAAC
HVT assembly RNA6 (99) TTAGTTCAGAGTAATGAACAAATAATCATGAATGACATCTCGTATTAAC
MVT assembly RNA6 (99) TTAGTTCAGAGTAATGAACAAATAATCATGAATGACATCTCGTATTAAC
Section 4
(148) 148 160 170 180 196
SRBSDV reference RNA6 (148) ATCTGCGCACTACCACCTAGCTGATTTGAGTTATTATACCATCTTTAAC
HVT assembly RNA6 (148) ATCTGCGCACTAACACCTAGCTGATTTGAGTTATTACACCATCTTTAAC
MVT assembly RNA6 (148) ATTTGCGCACTAACACCTAGCTGATTTGAGTTATTACACCATCTTTAAC
Section 5
(197) 197 210 220 230 245
SRBSDV reference RNA6 (197) GGACAGAGATCTCATGTTGAACTGGATTATCGGTATTCGAGATGGTAAG
HVT assembly RNA6 (197) GGACAGAGATCTCATGTTGAACTGGATTATCGGTATTCGTGATGGTAAG
MVT assembly RNA6 (197) GGACAGAGATCTCATGTTGAACTGGATTATCGGTATTCGTGATGGTAAG
Section 6
(246) 246 260 270 280 294
SRBSDV reference RNA6 (246) TCAGTTTTCTCTATTCGTCACCTTGCTCGGCAAGAGACTCAAATCAGCT
HVT assembly RNA6 (246) TCAGTTTTCTCTATTCGTCACCTTGCCCGGCAAGAGACTCAAATCAGCT
MVT assembly RNA6 (246) TCAGTTTTCTCTATTCGTCACCTTGCCCGGCAAGAGACTCAAATCAGCT
Section 7
(295) 295 300 313
SRBSDV reference RNA6 (295) GATGT--------------
HVT assembly RNA6 (295) GATTTGAGTTATTACACCA
MVT assembly RNA6 (295) GATTTGAGTTATTACACCA
14nt

RNA7

```
                                                          Section 1
                       (1) 1        10        20        30                49
SRBSDV reference RNA7  (1) AATTTCATGTCTTTTATTAAATGTCACACGATTACCGTTCCTAAATGGA
    HVT assembly RNA7  (1) AATTTCATGTCTTTTATTAAATGTCACACGATTACCGTTCCTAAATGGA
    MVT assembly RNA7  (1) AATTTCATGTCTTTTATTAAATGTCACACGATTACCGTTCCTAAATGGA
                                                          Section 2
                      (50) 50        60        70        80               98
SRBSDV reference RNA7 (50) AGAAAATTGTCGTTCAATTTTATTTACGGCAAATATTTAGTAGAAGCAG
    HVT assembly RNA7 (50) AGAAAATTGTCGTTCAATTTTATTTACGGCAAATATTTAGTAGAAGCAG
    MVT assembly RNA7 (50) AGAAAATTGTCGTTCAATTTTATTTACGGCAAATATTTAGTAGAAGCAG
                                                          Section 3
                      (99) 99         110       120       130             147
SRBSDV reference RNA7 (99) AACGCAAGTATTAATGGCACATGTGGATATAGATCATTGGTATGATGTT
    HVT assembly RNA7 (99) AACGCAAGTATTAATGGCACATGTGGATATAGATCATTGGTATGATGTT
    MVT assembly RNA7 (99) AACGCAAGTATTAATGGCACATGTGGATATAGATCATTGGTATGATGTT
                                                          Section 4
                     (148) 148         160       170       180            196
SRBSDV reference RNA7 (148) TTTATGAAAACCTTGGTTTTTAAGAGTATGAGAAAAACGAAAAAGATGT
    HVT assembly RNA7 (148) TTTATGAAAACCTTGGTTTTTAAGAGTATGAGAAAAACGAAAAAGATGT
    MVT assembly RNA7 (148) TTTATGAAAACCTTGGTTTTTAAGAGTATGAGAAAAACGAAAAAGATGT
                                                          Section 5
                     (197) 197          210       220       230           245
SRBSDV reference RNA7 (197) TGAAAAACATTCTGAATTTGTAATGTCTCGTCGAGGTGGTATGTCCTGT
    HVT assembly RNA7 (197) TGAAAAACATTCTGAATTTGTAATGTCTCGTCGAGGTGGTATGTCCTGT
    MVT assembly RNA7 (197) TGAAAAACATTCTGAATTTGTAATGTCTCGTCGAGGTGGTATGTCCTGT
                                                          Section 6
                     (246) 246           260       270       280          294
SRBSDV reference RNA7 (246) TTTTAATGTTGGTGGAATCTCAGACATGTGTCAAGGTCGAAATGCAGCT
    HVT assembly RNA7 (246) TTTTAATGTTGGTGGAATCTCAGACATGTGTCAAGGTCGAAATGCAGCT
    MVT assembly RNA7 (246) TTTTAATGTTGGTGGAATCTCAGACATGTGTCAAGGTCGAAATGCAGCT
                                                          Section 7
                     (295) 295  300             316
SRBSDV reference RNA7 (295) GATGTC----------------
    HVT assembly RNA7 (295) GATGTTTTTATGAAAACCTTGG
    MVT assembly RNA7 (295) GATGTTTTTATGAAAACCTTGG 16nt
```

RNA8

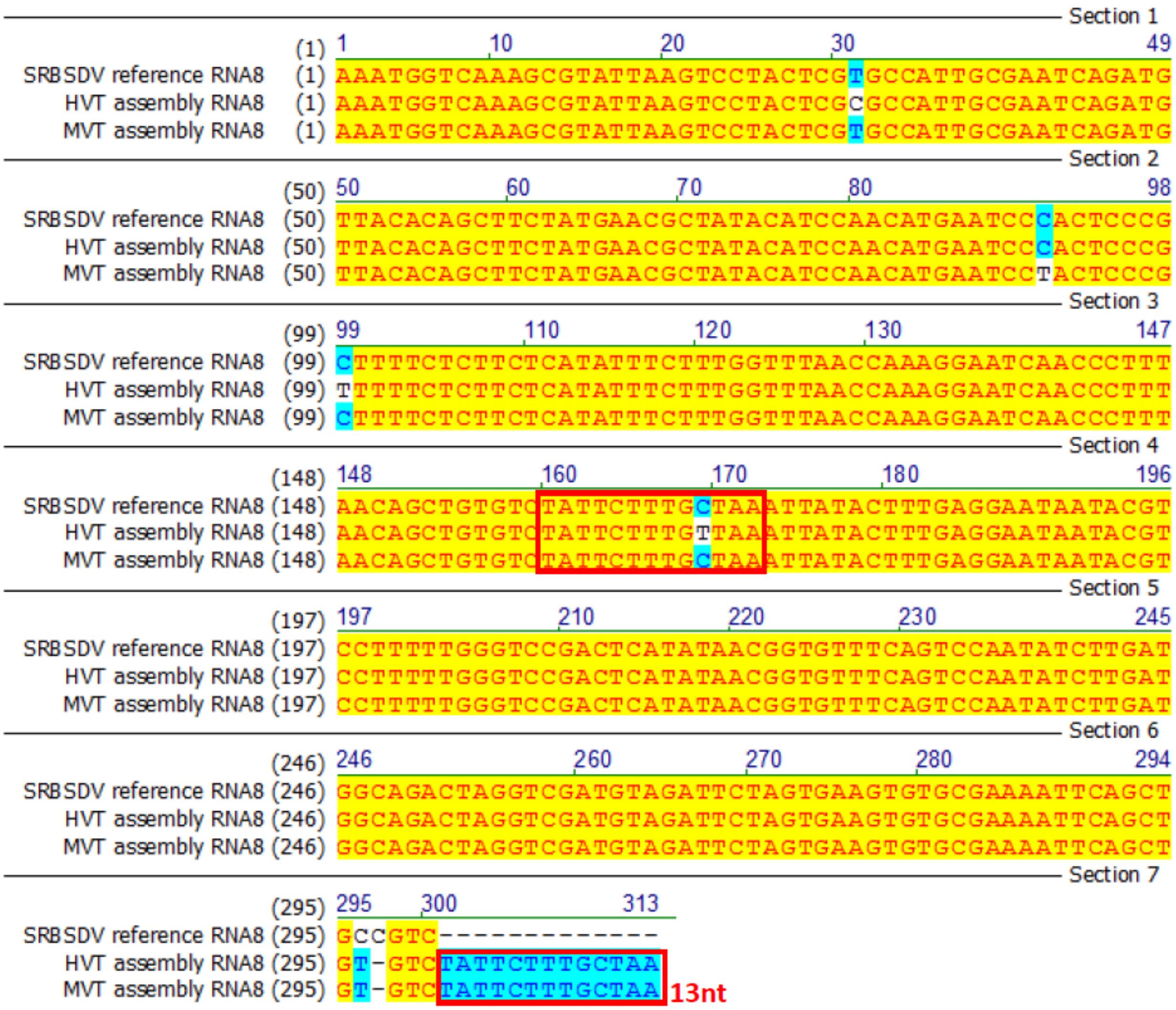

Section 1
(1) 1 10 20 30 49
SRBSDV reference RNA8 (1) AAATGGTCAAAGCGTATTAAGTCCTACTCGTGCCATTGCGAATCAGATG
HVT assembly RNA8 (1) AAATGGTCAAAGCGTATTAAGTCCTACTCGCGCCATTGCGAATCAGATG
MVT assembly RNA8 (1) AAATGGTCAAAGCGTATTAAGTCCTACTCGTGCCATTGCGAATCAGATG
Section 2
(50) 50 60 70 80 98
SRBSDV reference RNA8 (50) TTACACAGCTTCTATGAACGCTATACATCCAACATGAATCCCACTCCCG
HVT assembly RNA8 (50) TTACACAGCTTCTATGAACGCTATACATCCAACATGAATCCCACTCCCG
MVT assembly RNA8 (50) TTACACAGCTTCTATGAACGCTATACATCCAACATGAATCCTACTCCCG
Section 3
(99) 99 110 120 130 147
SRBSDV reference RNA8 (99) CTTTTCTCTTCTCATATTTCTTTGGTTTAACCAAAGGAATCAACCCTTT
HVT assembly RNA8 (99) TTTTTCTCTTCTCATATTTCTTTGGTTTAACCAAAGGAATCAACCCTTT
MVT assembly RNA8 (99) CTTTTCTCTTCTCATATTTCTTTGGTTTAACCAAAGGAATCAACCCTTT
Section 4
(148) 148 160 170 180 196
SRBSDV reference RNA8 (148) AACAGCTGTGTCTATTCTTTGCTAAATTATACTTTGAGGAATAATACGT
HVT assembly RNA8 (148) AACAGCTGTGTCTATTCTTTGTTAAATTATACTTTGAGGAATAATACGT
MVT assembly RNA8 (148) AACAGCTGTGTCTATTCTTTGCTAAATTATACTTTGAGGAATAATACGT
Section 5
(197) 197 210 220 230 245
SRBSDV reference RNA8 (197) CCTTTTTGGGTCCGACTCATATAACGGTGTTTCAGTCCAATATCTTGAT
HVT assembly RNA8 (197) CCTTTTTGGGTCCGACTCATATAACGGTGTTTCAGTCCAATATCTTGAT
MVT assembly RNA8 (197) CCTTTTTGGGTCCGACTCATATAACGGTGTTTCAGTCCAATATCTTGAT
Section 6
(246) 246 260 270 280 294
SRBSDV reference RNA8 (246) GGCAGACTAGGTCGATGTAGATTCTAGTGAAGTGTGCGAAAATTCAGCT
HVT assembly RNA8 (246) GGCAGACTAGGTCGATGTAGATTCTAGTGAAGTGTGCGAAAATTCAGCT
MVT assembly RNA8 (246) GGCAGACTAGGTCGATGTAGATTCTAGTGAAGTGTGCGAAAATTCAGCT
Section 7
(295) 295 300 313
SRBSDV reference RNA8 (295) GCCGTC-------------
HVT assembly RNA8 (295) GT-GTCTATTCTTTGCTAA
MVT assembly RNA8 (295) GT-GTCTATTCTTTGCTAA
13nt

Fig. S5 The trends in the changes of the relative ratios of RNA1 (a) and RNA2 (b) to the extended 3'-terminal sequences in (*Triticum aestivum* jingdong 22) and tobacco (*Nicotiana benthamiana*). The ratios of RNA1 and RNA2 to the extended 3'-terminal sequences were determined by associating a relative grey value of 3'-ETS to those of the RNA levels of RNA1 or RNA2 using ImageJ based on Fig. 4c and 4d. The differences were statistically evaluated by one-way ANOVAs for multiple comparisons with SPSS 17.0 software. The values are represented as the means ± SEs.

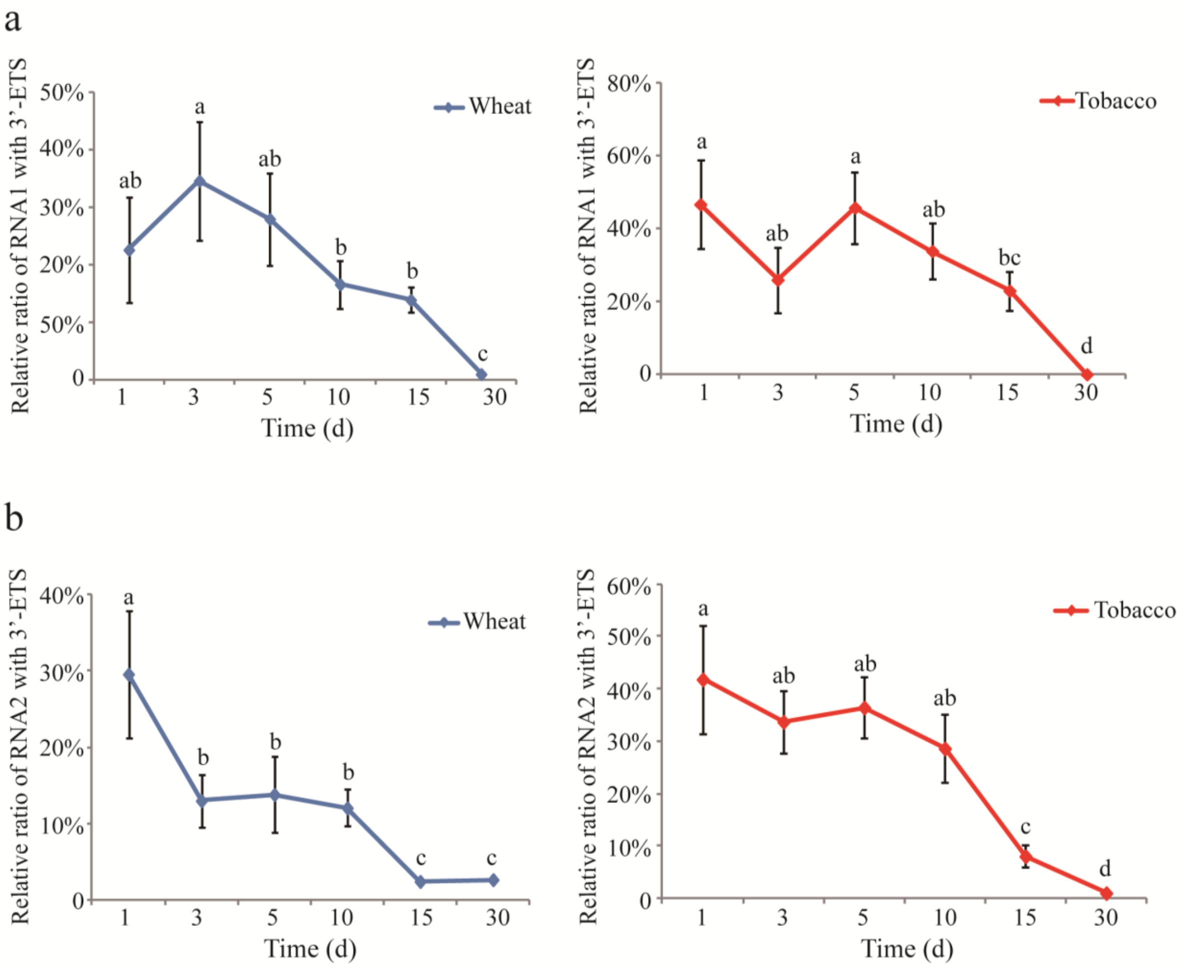

Table S1 Primers used in this study

Primers	Sequences
RNA1-GSP	GTGGCACCTTTAACTGATGAGGCAGTGG
RNA2-GSP	CTGAAACCCGTTGACCAAGTTGGG
RNA3-GSP	GTAGGAAGTCTGTACTCTTCTGGGAG
RNA4-GSP	GGGTGTCAGTCTCCAAGGGGTGATG
RNA1-nested GSP	GGAGGTGTTCAAGAGTAGCTTAGCC
RNA2-nested GSP	CAGTTGCTATGGCGAGAGCATGTGCTC
RNA3-nested GSP	GGAAGGGTGCCTAGATGAGTGAATGTC
RNA5-nested GSP	CTCGGGCAGAGTGTCATACAATAGAGTG
RNA1-3'F	GTCTGGCTATCATTCTCAT
RNA1-3'R	CCCTTGCATGTTGCTGA
RNA2-3'F	CCTAGTCTCAACAGCATAC
RNA2-3'R	ACTTTGAGACATGCTACACAAAGTCA
RNA3-3'F	TTCTTTGACTTGCATGTGATGA
RNA3-3'R	ACACAAAGTCTGGGTAATAAAAT
RNA4-3'F	AGCACCTCCAACAGCTTTCT
RNA4-3'R	ACACAAAGTCAGGGCATATCTT
RNA1-inner-F	TCTTCAGCCAGGAAGCTCTCTATAG
RNA1-inner-R	GAATGGGAGAGTTGTTAATCAGAC
RNA2-inner-F	ACATCAGTAGATGTCTTGAAGCAGA
RNA2-inner-R	AAATCATAAATCATGGTGTTGCA
RNA1-5'NTR-F	ACACAAAGTCCAGAGGAAAACAA
RNA1-5'NTR-R	TCACGTTTTATCCCTTATGACTT
RNA2-IR-F	CCACAGGCACACACACACACTGGCTAC
RNA2-IR-R	TGTAGCCAGTGTGTGTGTGTGCCTGTG
ubq-F	TCACCTACGTCTACAACCAG
ubq-R	AGTGCTGATCGTATTGACAGA
actin-insect-F	CCGCCTCCTCCAGTTCAC
actin-insect-R	TGTCCACGTCGCACTTCAT
actin-rice-F	CAGCCACACTGTCCCCATCTA
actin-rice-R	AGCAAGGTCGAGACGAAGGA
actin-wheat-F	CAAATCATGTTTGAGACCTTCAATG
actin-wheat-R	ACCAGAATCCAACACGATACCTG
actin-tobacco-F	TCCTGATGGGCAAGTGATTAC
actin- tobacco -R	TTGTATGTGGTCTCGTGGATTC
CP-RT-F	GTTCAGTCTAGTCATCTGCAC
CP-RT-R	ACACAAAGTCTGGGT

Table S2 Summary statistics of the RNA sequencing data from the Rice stripe virus (RSV)-infected small brown planthoppers (Laodelphax striatellus) and rice (Oryza sativa Huangjinqing)

Samples*	Raw Data		After quality control			RSV mapped reads			RSV mapped in proper read pair		
	Reads	Bases	Reads	Bases	%	Reads	Bases	%	Reads	Bases	%
In5d	130862672	13086267200	115586652	9888233945	88.33	953253	58415220	0.82	949532	58309165	0.82
InQ-1	129859358	12985935800	110966694	9544228634	85.45	541592	34223730	0.49	540046	34166391	0.49
InQ-2	245831016	24583101600	232335840	21977231395	94.51	291044	24744782	0.13	286593	24508599	0.12
InQ-3	200350334	20035033400	185893096	17520045844	92.78	242951	22001632	0.13	236529	21564136	0.13
InQ-4	227441528	22744152800	213166892	20045841756	93.72	293299	24945524	0.14	289881	24766907	0.14
P-1	129534168	12953416800	118134800	10506453506	91.20	1654356	79358543	1.40	1646909	79254945	1.39
P-2	211513964	21151396400	195207326	18138740102	92.29	34046	3169927	0.02	33892	3156428	0.02
P-3	240763002	24076300200	223555304	20786465161	92.85	55679	5162745	0.02	55434	5140712	0.02
P-4	257171876	25717187600	242810762	22765591526	94.42	36179	3386988	0.01	35991	3369817	0.01

* In5d, small brown planthoppers acquiring RSV of Jiangsu isolate (JSHA) for 5 d; InQ, viruliferous planthoppers, four replicates; P, RSV-infected rice leaves with typical stripe symptoms, four replicates.

Table S3 Coverages and depths of the newly assembled genome by Rice stripe virus (RSV) reads from different samples.

	Length	No. Covered bases	Breadth of coverage	Total base mapped	Average Depth
**In5d**					
genome	17155	17135	0.998834	58309165	3398.960
RNA1	8985	8968	0.998108	8406453	935.6097
RNA2	3532	3532	1	18375364	5202.538
RNA3	2488	2488	1	15291470	6146.089
RNA4	2150	2147	0.998605	16235878	7551.571
**InQ-1**					
genome	17155	17116	0.997727	34166391	1991.629
RNA1	8985	8969	0.998219	3385135	376.7540
RNA2	3532	3517	0.995753	4935487	1397.363
RNA3	2488	2486	0.999196	11412212	4586.902
RNA4	2150	2144	0.997209	14433557	6713.282
**InQ-2**					
genome	17155	17126	0.998309531	24508599	1428.65631
RNA1	8985	8961	0.997328881	1511095	168.179744
RNA2	3532	3531	0.999716874	2956956	837.1902605
RNA3	2488	2486	0.999196141	8725066	3506.859325
RNA4	2150	2148	0.999069767	11315482	5263.014884
**InQ-3**					
genome	17155	17120	0.997959778	21564136	1257.017546
RNA1	8985	8964	0.997662771	1271584	141.5229827
RNA2	3532	3518	0.99603624	2509946	710.6302378
RNA3	2488	2488	1	7184683	2887.734325
RNA4	2150	2150	1	10597923	4929.266512
**InQ-4**					
genome	17155	17115	0.997668318	24766907	1443.713611
RNA1	8985	8961	0.997328881	1546972	172.1727323
RNA2	3532	3518	0.99603624	2995942	848.2281993
RNA3	2488	2488	1	8749746	3516.778939
RNA4	2150	2148	0.999069767	11474247	5336.85907
**P-1**					
genome	17155	17155	1	79254945	4619.933
rice_RNA1	8985	8985	1	19690646	2191.502
rice_RNA2	3532	3532	1	25831892	7313.672
rice_RNA3	2488	2488	1	17335673	6967.714
rice_RNA4	2150	2150	1	16396734	7626.388
**P-2**					
genome	17155	17093	0.996385893	3156428	183.9946371
RNA1	8985	8961	0.997328881	402372	44.78263773
RNA2	3532	3506	0.992638732	728354	206.2157418
RNA3	2488	2479	0.996382637	933783	375.3147106
RNA4	2150	2147	0.998604651	1091919	507.8693023
**P-3**					
genome	17155	17092	0.996327601	5140712	299.6626057
RNA1	8985	8960	0.997217585	635345	70.71174179
RNA2	3532	3511	0.99405436	1207464	341.8640997
RNA3	2488	2481	0.997186495	1507665	605.9746785
RNA4	2150	2140	0.995348837	1790238	832.6688372
**P-4**					
genome	17155	17086	0.995977849	3369817	196.4335179
RNA1	8985	8958	0.996994992	496181	55.22326099
RNA2	3532	3506	0.992638732	782187	221.457248
RNA3	2488	2483	0.997990354	943974	379.4107717
RNA4	2150	2139	0.994883721	1147475	533.7093023

* In5d, small brown planthoppers acquiring RSV of Jiangsu isolate (JSHA) for 5 d; InQ, viruliferous planthoppers, four replicates; P, RSV-infected rice leaves with typical stripe symptom, four replicates. The newly assembled genome with the longest length was used as the reference for mapping.

References:

Kakutani T, Hayano Y, Hayashi T, Minobe Y. 1991. Ambisense segment 3 of rice stripe virus: the first instance of a virus containing two ambisense segments. *Journal of General Virology* **72**: 465-468.

Qu Z, Liang D, Harper G, Hull R. 1997. Comparison of sequences of RNAs 3 and 4 of rice stripe virus from China with those of Japanese isolates. *Virus Genes* **15**: 99-103.

Wang L, Valderramos SG, Wu A, Ouyang S, Li C, Brasil P, Bonaldo M, Coates T, Nielsen-Saines K, Jiang T, et al. 2016b. From mosquitos to humans: genetic evolution of Zika virus. *Cell Host & Microbe* **19**: 561-565.

Wei TY, Yang JG, Liao FR, Gao FL, Lu LM, Zhang XT, Li F, Wu ZJ, Lin QY, Xie LH, et al. 2009. Genetic diversity and population structure of rice stripe virus in China. *Journal of General Virology* **90**: 1025-1034.

Zhu Y, Hayakawa T, Toriyama S, Takahashi M. 1991. Complete nucleotide sequence of RNA 3 of rice stripe virus: an ambisense coding strategy. *Journal of General Virology* **72**: 763-767.
